# Untapped Potential of Marine-Associated *Cladosporium* Species: An Overview on Secondary Metabolites, Biotechnological Relevance, and Biological Activities

**DOI:** 10.3390/md19110645

**Published:** 2021-11-18

**Authors:** Gamal A. Mohamed, Sabrin R. M. Ibrahim

**Affiliations:** 1 Department of Natural Products and Alternative Medicine, Faculty of Pharmacy, King Abdulaziz University, Jeddah 21589, Saudi Arabia; 2 Preparatory Year Program, Batterjee Medical College, Jeddah 21442, Saudi Arabia; sabrin.ibrahim@bmc.edu.sa; 3 Department of Pharmacognosy, Faculty of Pharmacy, Assiut University, Assiut 71526, Egypt

**Keywords:** bioactivity, biotechnology, *Cladosporium*, Cladosporiaceae, marine fungi, metabolite

## Abstract

The marine environment is an underexplored treasure that hosts huge biodiversity of microorganisms. Marine-derived fungi are a rich source of novel metabolites with unique structural features, bioactivities, and biotechnological applications. Marine-associated *Cladosporium* species have attracted considerable interest because of their ability to produce a wide array of metabolites, including alkaloids, macrolides, diketopiperazines, pyrones, tetralones, sterols, phenolics, terpenes, lactones, and tetramic acid derivatives that possess versatile bioactivities. Moreover, they produce diverse enzymes with biotechnological and industrial relevance. This review gives an overview on the *Cladosporium* species derived from marine habitats, including their metabolites and bioactivities, as well as the industrial and biotechnological potential of these species. In the current review, 286 compounds have been listed based on the reported data from 1998 until July 2021. Moreover, more than 175 references have been cited.

## 1. Introduction

The marine environment covers approximately 70% of the Earth’s surface and represents an enormous pool of biodiversity resources [1,2,3]. Marine microorganisms possess the potential for several biotechnological and industrial applications and play an important ecological role [4,5]. The last decades have witnessed numerous studies in the natural metabolites derived from marine creatures or their associated microorganisms [6,7,8]. Marine-derived fungi consist of a wide range of parasites, saprotrophs, symbionts, epiphytes, and endophytes [9,10]. They can be obtained from various marine samples such as algae, seagrasses, corals, sponges, ascidians, crustaceans, bivalves, fishes, and inorganic matter [11,12]. Jones et al. reported 530 marine taxa in 321 genera, which included 12 Basidiomycota (nine genera), 94 asexual morphs (61 genera), and 424 Ascomycota (251 genera) [13]. In 2011, the number of marine fungi was estimated to be 10,000 to 12,500 species based on substrates and geographical locations [14]. Currently, 1901 species have been listed on the marine fungi website, in 769 genera, 88 orders, 226 families, 22 classes, and seven phyla [15]. They are acknowledged as a rich source of novel metabolites with unique structural features, bioactivities, and biotechnological applications that attracted the attention of many biologists and chemists [16]. *Cladosporium* (Cladosporiaceae) is one of the largest genera of dematiaceous hyphomycetes [17]. *Cladosporium* species are frequent airborne molds, which can be isolated from almost every environment and geographic location, because their small conidia are easily dispersed [18,19,20,21]. *C. herbarum*, *C. cladosporioides*, and *C. sphaerospermum* are its three major species [22]. It comprises many important plant pathogens causing stem rots and leaf spots such as *C. fulvum* is the causal agent of tomato leaf mold [23,24]. Some species are also known as common contaminants in clinical laboratories and cause allergic lung diseases [25,26,27,28]. Some species have been reported as endophytes and possessed a positive influence, for example, *C. sphaerospermum* isolated from *Glycine max* roots which can promote its growth [29]. Several species were linked to allergic rhinitis and respiratory arrest in asthmatic patients, and some are described as a cause of opportunistic phaeohyphomycosis, including subcutaneous and deep infections in humans and animals [30,31]. Some species are fungicolous that possess a potential for biological control in agriculture and forestry [32,33]. Moreover, many *Cladosporium* species have the potential to be used in various industrial processes [34,35]. Marine-associated *Cladosporium* species have attracted considerable interest because of their ability to produce a wide array of metabolites, including macrolides, pyrones, phenolics, alkaloids, diketopiperazines, terpenes, sterols, quinones, lactones, and tetramic acid derivatives. These metabolites possess versatile bioactivities such as anticancer, antimicrobial, antiviral, insecticidal, antifouling, anti-malarial, anti-hyperlipidemic, and α-glucosidase and protein tyrosine phosphatase inhibiton [36,37,38,39,40,41,42]. It has been shown that these species have significant impacts on biotechnology, ecosystems, and food production. They are a wealthy source of enzymes such as pectinases, agarases, carrageenases, xylanases, laccases, peroxidases, tannases, invertases, cellulases, and reductases that have wide biotechnological influences in developing eco-friendly technologies in the pulp and paper industry, food and feed industries, biomasses and contaminants bioremediation and biodegradation, and generation chemicals and liquid fuels [11,12,43,44,45,46,47,48,49,50]. The main goal of this review is the focus on the reported research in *Cladosporium* species derived from a marine habitat, including the structures and bioactivities of the reported metabolites, as well as the industrial and biotechnological potential of these species (Table 1 and Table 2). This work covers the studies that have appeared in literature from 1998 until July 2021. The structures and bioactivities of reported metabolites from *Cladosporium* species have been highlighted. Furthermore, the biotechnological and industrial potential of *Cladosporium* species has been summarized. We hope that this work can provide knowledge that can help for the dereplication and bioactivities evaluation of these marine-associated *Cladosporium* species. The present data were collected through the search on the various databases, including Web of Knowledge, ScienceDirect, SCOPUS, Taylor & Francis, Wiley Online Library, PubMed, JACS, Springer, and Google Scholar.

## 2. Importance of Marine Associated *Cladosporium* Species

Recently, cold-active microbial enzymes have attracted a great attention, and they are preferred to the thermophilic and mesophilic enzymes due to the reduction in the energy expenditure and costs of processing accompanied by industrial heating steps [51]. Many marine-associated *Cladosporium* species display noticeable enzyme production capacity. Many of these enzymes are exclusively produced at low temperature and high salt concentrations. Therefore, they play a substantial ecological role in lignin-cellulosic materials decomposition in the marine environment. Besides, these enzymes can be utilized in various biotechnological applications and allow the performance of industrial processes even in harsh conditions. In this review, the biotechnological and industrial relevance of *Cladosporium* species has been highlighted.

The polycyclic aromatic hydrocarbons (PAHs) are volatile pollutants that can cause various environmental pollutions such as oceanic and freshwater contamination, which can take place during storage, use, or transportation of crude oil and its products. PAHs inhalation or ingestion through contaminated food and airborne contaminants leads to serious health disorders such as endocrine disruption, cancer, and reproductive and birth problems [52]. Therefore, introducing marine-adapted microorganisms to increase the PAH-biodegradation rate is an important approach to reduce PAHs concentration in the contaminated regions. Investigation of the PAH biodegradation potential of various marine-derived fungi revealed that *Cladosporium* sp. CBMAI 1237 had a great potential for bioremediation and biodegradation of PAHs (e.g., anthracene, anthrone, anthraquinone, acenaphthene, phenanthrene, fluorene, pyrene fluoranthene, and nitropyrene) even in a non-marine environment [44].

**Table 1:** Secondary metabolites reported from marine associated *Cladosporium* species.

Compound Name	Mol. Wt.	Mol. Formula	Fungal Source	Host (Sample, Family)	Place	Ref.
1. Tetramic acid derivatives						
Cladosin A (**1**)	282	C_14_H_22_N_2_O_4_	*C. sphaerospermum* 2005-01-E3	Deep-sea sludge, Pacific Ocean	Qingdao, China	[42]
Cladosin B (**2**)	268	C_13_H_20_N_2_O_4_	*C. sphaerospermum* 2005-01-E3	Deep-sea sludge, Pacific Ocean	Qingdao, China	[42]
			*C. sphaerospermum* SW67	*Hydractinia echinata* (Marine hydroid, Hydractiniidae)	South Korea	[53]
Cladosin C (**3**)	250	C_13_H_18_N_2_O_3_	*C. sphaerospermum* 2005-01-E3	Deep-sea sludge, Pacific Ocean	Qingdao, China	[42]
			*C. sphaerospermum* SW67	*Hydractinia echinata* (Marine hydroid, Hydractiniidae)	South Korea	[53]
Cladosin D (**4**)	250	C_13_H_18_N_2_O_3_	*C. sphaerospermum* 2005-01-E3	Deep-sea sludge, Pacific Ocean	Qingdao, China	[42]
Cladosin F (**5**)	268	C_13_H_20_N_2_O_4_	*C. sphaerospermum* 2005-01-E3	Deep-sea sludge, Pacific Ocean	Qingdao, China	[54]
			*C. sphaerospermum* SW67	*Hydractinia echinata* (Marine hydroid, Hydractiniidae)	South Korea	[53]
Cladosin G (**6**)	282	C_14_H_22_N_2_O_4_	*C. sphaerospermum* 2005-01-E3	Deep-sea sludge, Pacific Ocean	Qingdao, China	[54]
Cladosin H (**7**)	358	C_20_H_26_N_2_O_4_	*C. sphaerospermum* L3P3	Marine sediment	Mariana Trench, South Pacific Ocean, China	[55]
Cladosin I (**8**)	358	C_20_H_26_N_2_O_4_	*C. sphaerospermum* L3P3	Marine sediment	Mariana Trench, South Pacific Ocean, China	[55]
Cladosin J (**9**)	419	C_25_H_29_N_3_O_3_	*C. sphaerospermum* L3P3	Marine sediment	Mariana Trench, South Pacific Ocean, China	[55]
Cladosin K (**10**)	419	C_25_H_29_N_3_O_3_	*C. sphaerospermum* L3P3	Marine sediment	Mariana Trench, South Pacific Ocean, China	[55]
Cladosin L (**11**)	270	C_13_H_22_N_2_O_4_	*C. sphaerospermum* SW67	*Hydractinia echinata* (Marine hydroid, Hydractiniidae)	South Korea	[53]
Cladosporicin A (**12**)	401	C_21_H_27_N_3_O_5_	*C. sphaerospermum* SW67	*Hydractinia echinata* (Marine hydroid, Hydractiniidae)	South Korea	[38]
Cladodionen (**13**)	233	C_13_H_15_NO_3_	*Cladosporium* sp. OUCMDZ-1635	Unidentified sponge	Xisha Islands, China	[56]
			*C*. *sphaerospermum* EIODSF 008.	Deep sea sediment	East Indian Ocean, China	[57]
			*C. sphaerospermum* L3P3	Marine sediment	Mariana Trench, South Pacific Ocean, China	[55]
Cladosporiumin A (**14**)	349	C_19_H_27_NO_5_	*Cladosporium* sp. SCSIO z0025	Deep sea sediment	Okinawa, Japan	[58]
Cladosporiumin B (**15**)	349	C_19_H_27_NO_5_	*Cladosporium* sp. SCSIO z0025	Deep sea sediment	Okinawa, Japan	[58]
Cladosporiumin C (**16**)	349	C_19_H_27_NO_5_	*Cladosporium* sp. SCSIO z0025	Deep sea sediment	Okinawa, Japan	[58]
Cladosporiumin D (**17**)	253	C_13_H_19_NO_4_	*Cladosporium* sp. SCSIO z0025	Deep sea sediment	Okinawa, Japan	[58]
Cladosporiumin E (**18**)	251	C_13_H_17_NO_4_	*Cladosporium* sp. SCSIO z0025	Deep sea sediment	Okinawa, Japan	[58]
Cladosporiumin F (**19**)	269	C_13_H_19_NO_5_	*Cladosporium* sp. SCSIO z0025	Deep sea sediment	Okinawa, Japan	[58]
Cladosporiumin G (**20**)	253	C_13_H_19_NO_4_	*Cladosporium* sp. SCSIO z0025	Deep sea sediment	Okinawa, Japan	[58]
Cladosporiumin H (**21**)	285	C_14_H_23_NO_5_	*Cladosporium* sp. SCSIO z0025	Deep sea sediment	Okinawa, Japan	[58]
Cladosporiumin I (**22**)	235	C_13_H_17_NO_3_	*C*. *sphaerospermum* EIODSF 008.	Deep sea sediment	East Indian Ocean, China	[57]
Cladosporiumin J (**23**)	251	C_13_H_17_NO_4_	*C*. *sphaerospermum* EIODSF 008.	Deep sea sediment	East Indian Ocean, China	[57]
Cladosporiumin K (**24**)	251	C_13_H_17_NO_4_	*C*. *sphaerospermum* EIODSF 008.	Deep sea sediment	East Indian Ocean. China	[57]
Cladosporiumin L (**25**)	887	C_41_H_65_N_3_O_15_Mg_2_	*C*. *sphaerospermum* EIODSF 008.	Deep sea sediment	East Indian Ocean, China	[57]
Cladosporiumin M (**26**)	233	C_13_H_15_NO_3_	*C*. *sphaerospermum* EIODSF 008.	Deep sea sediment	East Indian Ocean, China	[57]
Cladosporiumin N (**27**)	253	C_13_H_19_NO_4_	*C*. *sphaerospermum* EIODSF 008.	Deep sea sediment	East Indian Ocean. China	[57]
Cladosporiumin O (**28**)	251	C_13_H_17_NO_4_	*C*. *sphaerospermum* EIODSF 008.	Deep sea sediment	East Indian Ocean, China	[57]
Cladosporiumin I (**29**)	349	C_19_H_27_NO_5_	*C. sphaerospermum SW67*	*Hydractinia echinata* (Marine hydroid, Hydractiniidae)	South Korea	[38]
Cladosporiumin J (**30**)	349	C_19_H_27_NO_5_	*C. sphaerospermum SW67*	*Hydractinia echinata* (Marine hydroid, Hydractiniidae)	South Korea	[38]
2. Diketopiperazines						
Cyclo-(Pro, Trp) (**31**)	283	C_16_H_17_N_3_O_2_	*Cladosporium* sp. EF424419	*Porphyra yezoensis* (Red alga, Bangiaceae)	Lianyungang, Jiangsu, China	[59]
Cyclo-(Val-Pro) (**32**)	196	C_10_H_16_N_2_O_2_	*Cladosporium* sp. EF424419	*Porphyra yezoensis* (Red alga, Bangiaceae)	Lianyungang, Jiangsu, China	[59]
Cyclo-(Phe-Pro) (**33**)	244	C_14_H_16_N_2_O_2_	*Cladosporium* sp. F14	Seawater from mangrove stand	Kei Ling Ha Lo Wai, Sai Kung, China	[60]
Cyclo-(Phe-Val) (**34**)	246	C_14_H_18_N_2_O_2_	*Cladosporium* sp. F14	Seawater from mangrove stand	Kei Ling Ha Lo Wai, Sai Kung, China	[60]
Cyclo-(Gly-Leu) (**35**)	170	C_8_H_14_N_2_O_2_	*Cladosporium* sp. SCSIO41007	*Callyspongia* sp. (Sponge, Callyspongiidae)	Xuwen, Guangdong, China	[61]
Cladosporin A (**36**)	460	C_22_H_24_N_2_O_5_S_2_	*Cladosporium* sp.	Marine sediment	Yangshashan Bay, Ningbo, Zhejiang, China	[62]
Cladosporin B (**37**)	442	C_22_H_22_N_2_O_4_S_2_	*Cladosporium* sp.	Marine sediment	Yangshashan Bay, Ningbo, Zhejiang, China	[62]
Haematocin (**38**)	502	C_24_H_26_N_2_O_6_S_2_	*Cladosporium* sp.	Marine sediment	Yangshashan Bay, Ningbo, Zhejiang, China	[62]
3. Alkaloids						
3.1. Indole alkaloids						
3.1.1 Simple indole alkaloids						
N-Acetyltryptamine (**39**)	202	C_12_H_14_N_2_O	*Cladosporium* sp. EF424419	*Porphyra yezoensis* (Red alga, Bangiaceae)	Lianyungang, Jiangsu, China	[59]
N-methyl-1*H*-indole-2-carboxamide (**40**)	174	C_10_H_10_N_2_O	*C. cladosporioides*	*Cliona* sp. (Sponge, Clionaidae)	Los Molles, Chile	[63]
Indole-3-carboxylic acid (**41**)	161	C_9_H_7_NO_2_	*Cladosporium* sp. SCSIO41007	*Callyspongia* sp. (Sponge, Callyspongiidae)	Xuwen, Guangdong, China	[61]
3.1.2 Glyantrypine derivatives						
Glyantrypine (**42**)	344	C_20_H_16_N_4_O_2_	*Cladosporium* sp. PJX-41	Soil around a mangrove	Guangzhou, China	[64]
3-Hydroxyglyantrypine (**43**)	360	C_20_H_16_N_4_O_3_	*Cladosporium* sp. PJX-41	Soil around a mangrove	Guangzhou, China	[64]
14*R*-Oxoglyantrypine (**44**)	358	C_20_H_14_N_4_O_3_	*Cladosporium* sp. PJX-41	Soil around a mangrove	Guangzhou, China	[64]
14*S*-Oxoglyantrypine (**45**)	358	C_20_H_14_N_4_O_3_	*Cladosporium* sp. PJX-41	Soil around a mangrove	Guangzhou, China	[64]
Prelapatin B (**46**)	344	C_20_H_16_N_4_O_2_	*Cladosporium* sp. PJX-41	Soil around a mangrove	Guangzhou, China	[64]
Cladoquinazoline (**47**)	418	C_23_H_22_N_4_O_4_	*Cladosporium* sp. PJX-41	Soil around a mangrove	Guangzhou, China	[64]
*Epi*-Cladoquinazoline (**48**)	418	C_23_H_22_N_4_O_4_	*Cladosporium* sp. PJX-41	Soil around a mangrove	Guangzhou, China	[64]
3.2. Quinazoline alkaloids						
Norquinadoline A (**49**)	471	C_26_H_25_N_5_O_4_	*Cladosporium* sp. PJX-41	Soil around a mangrove	Guangzhou, China	[64]
Quinadoline A (**50**)	485	C_27_H_27_N_5_O_5_	*Cladosporium* sp. PJX-41	Soil around a mangrove	Guangzhou, China	[64]
Deoxynortryptoquivaline (**51**)	516	C_28_H_28_N_4_O_6_	*Cladosporium* sp. PJX-41	Soil around a mangrove	Guangzhou, China	[64]
Deoxytryptoquivaline (**52**)	530	C_29_H_30_N_4_O_6_	*Cladosporium* sp. PJX-41	Soil around a mangrove	Guangzhou, China	[64]
Tryptoquivaline (**53**)	546	C_29_H_30_N_4_O_7_	*Cladosporium* sp. PJX-41	Soil around a mangrove	Guangzhou, China	[64]
CS-C (**54**)	546	C_29_H_30_N_4_O_7_	*Cladosporium* sp. PJX-41	Soil around a mangrove	Guangzhou, China	[64]
Quinadoline B (**55**)	439	C_25_H_21_N_5_O_3_	*Cladosporium* sp. PJX-41	Soil around a mangrove	Guangzhou, China	[64]
Circumdatin A (**56**)	391	C_22_H_21_N_3_O_4_	*Cladosporium* sp. MFC353-b	*Chondria crassicualis* (Red alga, Rhodomelaceae)	Yokji Island, Kyeongnam, Korea	[65]
3.3. Quinolone alkaloids						
Quinolactacin A1 (**57**)	270	C_16_H_18_N_2_O_2_	*C. oxysporum* BRS2A-AR2F	*Conocarpus erectus* (Mangrove plant, Combretaceae) *Laguncularia racemosa* (Mangrove plant, Combretaceae) *Rhizophora racemosa* (Mangrove plant, Rhizophoraceae)	Banks of the River Butre, Western Region of Ghana	[66]
Quinolactacin A2 (**58**)	270	C_16_H_18_N_2_O_2_	*C. oxysporum* BRS2A-AR2F	*Conocarpus erectus* (Mangrove plant, Combretaceae) *Laguncularia racemosa* (Mangrove plant, Combretaceae) *Rhizophora racemosa* (Mangrove plant, Rhizophoraceae)	Banks of the River Butre, Western Region of Ghana	[66]
Quinolactacin B1 (**59**)	256	C_15_H_16_N_2_O_2_	*C. oxysporum* BRS2A-AR2F	*Conocarpus erectus* (Mangrove plant, Combretaceae) *Laguncularia racemosa* (Mangrove plant, Combretaceae) *Rhizophora racemosa* (Mangrove plant, Rhizophoraceae)	Banks of the River Butre, Western Region of Ghana	[66]
Quinolactacin B2 (**60**)	256	C_15_H_16_N_2_O_2_	*C. oxysporum* BRS2A-AR2F	*Conocarpus erectus* (Mangrove plant, Combretaceae) *Laguncularia racemosa* (Mangrove plant, Combretaceae) *Rhizophora racemosa* (Mangrove plant, Rhizophoraceae)	Banks of the River Butre, Western Region of Ghana	[66]
Quinolactacin C1 (**61**)	286	C_16_H_18_N_2_O_3_	*C. oxysporum* BRS2A-AR2F	*Conocarpus erectus* (Mangrove plant, Combretaceae) *Laguncularia racemosa* (Mangrove plant, Combretaceae) *Rhizophora racemosa* (Mangrove plant, Rhizophoraceae)	Banks of the River Butre, Western Region of Ghana	[66]
Quinolactacin C2 (**62**)	286	C_16_H_18_N_2_O_3_	*C. oxysporum* BRS2A-AR2F	*Conocarpus erectus* (Mangrove plant, Combretaceae) *Laguncularia racemosa* (Mangrove plant, Combretaceae) *Rhizophora racemosa* (Mangrove plant, Rhizophoraceae)	Banks of the River Butre, Western Region of Ghana	[66]
Quinolactacin D1 (**63**)	286	C_16_H_18_N_2_O_3_	*C. oxysporum* BRS2A-AR2F	*Conocarpus erectus* (Mangrove plant, Combretaceae) *Laguncularia racemosa* (Mangrove plant, Combretaceae) *Rhizophora racemosa* (Mangrove plant, Rhizophoraceae)	Banks of the River Butre, Western Region of Ghana	[66]
Quinolactacin D2 (**64**)	286	C_16_H_18_N_2_O_3_	*C. oxysporum* BRS2A-AR2F	*Conocarpus erectus* (Mangrove plant, Combretaceae) *Laguncularia racemosa* (Mangrove plant, Combretaceae) *Rhizophora racemosa* (Mangrove plant, Rhizophoraceae)	Banks of the River Butre, Western Region of Ghana	[66]
Quinocitrinine A (**65**)	272	C_16_H_19_N_2_O_2_	*C. oxysporum* BRS2A-AR2F	*Conocarpus erectus* (Mangrove plant, Combretaceae) *Laguncularia racemosa* (Mangrove plant, Combretaceae) *Rhizophora racemosa* (Mangrove plant, Rhizophoraceae)	Banks of the River Butre, Western Region of Ghana	[66]
Quinocitrinine B (**66**)	272	C_16_H_19_N_2_O_2_	*C. oxysporum* BRS2A-AR2F	*Conocarpus erectus* (Mangrove plant, Combretaceae) *Laguncularia racemosa* (Mangrove plant, Combretaceae) *Rhizophora racemosa* (Mangrove plant, Rhizophoraceae)	Banks of the River Butre, Western Region of Ghana	[66]
Quinolactacide (**67**)	236	C_14_H_8_N_2_O_2_	*C. oxysporum* BRS2A-AR2F	*Conocarpus erectus* (Mangrove plant, Combretaceae) *Laguncularia racemosa* (Mangrove plant, Combretaceae) *Rhizophora racemosa* (Mangrove plant, Rhizophoraceae)	Banks of the River Butre, Western Region of Ghana	[66]
3.4. Citrinadin derivatives						
Citrinadin A (**68**)	624	C_35_H_52_N_4_O_6_	*C. oxysporum* BRS2A-AR2F	*Conocarpus erectus* (Mangrove plant, Combretaceae) *Laguncularia racemosa* (Mangrove plant, Combretaceae) *Rhizophora racemosa* (Mangrove plant, Rhizophoraceae)	Banks of the River Butre, Western Region of Ghana	[66]
Citrinadin B (**69**)	481	C_28_H_39_N_3_O_4_	*C. oxysporum* BRS2A-AR2F	*Conocarpus erectus* (Mangrove plant, Combretaceae) *Laguncularia racemosa* (Mangrove plant, Combretaceae) *Rhizophora racemosa* (Mangrove plant, Rhizophoraceae)	Banks of the River Butre, Western Region of Ghana	[66]
Butrecitrinadin (**70**)	682	C_38_H_57_N_4_O_7_	*C. oxysporum* BRS2A-AR2F	*Conocarpus erectus* (Mangrove plant, Combretaceae) *Laguncularia racemosa* (Mangrove plant, Combretaceae) *Rhizophora racemosa* (Mangrove plant, Rhizophoraceae)	Banks of the River Butre, Western Region of Ghana	[66]
PF1270 A (**71**)	566	C_32_H_43_N_3_O_6_	*C. oxysporum* BRS2A-AR2F	*Conocarpus erectus* (Mangrove plant, Combretaceae) *Laguncularia racemosa* (Mangrove plant, Combretaceae) *Rhizophora racemosa* (Mangrove plant, Rhizophoraceae)	Banks of the River Butre, Western Region of Ghana	[66]
PF1270 B (**72**)	552	C_31_H_41_N_3_O_6_	*C. oxysporum* BRS2A-AR2F	*Conocarpus erectus* (Mangrove plant, Combretaceae) *Laguncularia racemosa* (Mangrove plant, Combretaceae) *Rhizophora racemosa* (Mangrove plant, Rhizophoraceae)	Banks of the River Butre, Western Region of Ghana	[66]
PF1270 C (**73**)	538	C_30_H_39_N_3_O_6_	*C. oxysporum* BRS2A-AR2F	*Conocarpus erectus* (Mangrove plant, Combretaceae) *Laguncularia racemosa* (Mangrove plant, Combretaceae) *Rhizophora racemosa* (Mangrove plant, Rhizophoraceae)	Banks of the River Butre, Western Region of Ghana	[66]
3.5. Pyrrolidine derivatives						
Cladosporitin A (**74**)	505	C_32_H_43_NO_4_	*Cladosporium* sp. HNWSW-1	*Ceriops tagal* (Mangrove plant, Rhizophoraceae)	Dong Zhai Gang, Hainan, China	[67]
Cladosporitin B (**75**)	505	C_32_H_43_NO_4_	*Cladosporium* sp. HNWSW-1	*Ceriops tagal* (Mangrove plant, Rhizophoraceae)	Dong Zhai Gang, Hainan, China	[67]
Talaroconvolutin A (**76**)	487	C_32_H_41_NO_3_	*Cladosporium* sp. HNWSW-1	*Ceriops tagal* (Mangrove plant, Rhizophoraceae)	Dong Zhai Gang, Hainan, China	[67]
Cladosporamide A (**77**)	273	C_14_H_11_NO_5_	*Cladosporium* sp. TPU1507	Unidentified marine sponge	Manado, Indonesia	[68]
3.6. Other class of alkaloids						
Cladosporilactam A (**78**)	181	C_10_H_15_NO_2_	*Cladosporium* sp. RA07-1	*Anthogorgia ochracea* (Gorgonian, Acanthogorgiidae)	Weizhou coral reef, South China Sea	[69]
Cladospamide A (**79**)	268	C_13_H_20_N_2_O_4_	*Cladosporium* sp. SCNU-F0001	Mangrove plant	Zhuhai Mangrove Nature, Guangdong, China	[70]
Cladosporin A (**80**)	233	C_13_H_15_NO_3_	*C. cladosporioides* SCSIO z015	Deep sea sediment	Okinawa, Japan	[36]
Cladosporin B (**81**)	233	C_13_H_15_NO_3_	*C. cladosporioides* SCSIO z015	Deep sea sediment	Okinawa, Japan	[36]
2′-Deoxythymidine (**82**)	241	C_11_H_15_NO_5_	*Cladosporium* sp. SCSIO41007	*Callyspongia* sp. (Sponge, Callyspongiidae)	Xuwen, Guangdong, China	[61]
Nicotinic acid (**83**)	123	C_6_H_5_NO_2_	*Cladosporium* sp. EF424419	*Porphyra yezoensis* (Red alga, Bangiaceae)	Lianyungang, Jiangsu, China	[59]
2-Methylacetate-3,5,6-trimethylpyrazine (**84**)	194	C_10_H_14_N_2_O_2_	*Cladosporium* sp. JS1-2	*Ceriops tagal* (Mangrove plant, Rhizophoraceae)	Dongzhaigang, Hainan, China	[71]
Cytochalasin D (**85**)	507	C_30_H_37_NO_6_	*Cladosporium* sp. JS1-2	*Ceriops tagal* (Mangrove plant, Rhizophoraceae)	Dongzhaigang, Hainan, China	[71]
Cladosin E (**86**)	251	C_13_H_17_NO_4_	*C. sphaerospermum* 2005-01-E3	Deep-sea sludge, Pacific Ocean	Qingdao, China	[42]
N-Acetyltyramine (**87**)	179	C_10_H_13_NO_2_	*Cladosporium* sp. EF424419	*Porphyra yezoensis* (Red alga*,* Bangiaceae)	Lianyungang, Jiangsu, China	[59]
4. Macrolides						
Cladospolide A (**88**)	228	C_12_H_20_O_4_	*Cladosporium* sp. FT-0012	Sponge	Pohnpei island, Federated State of Micronesia	[72]
			*Cladosporium* sp. IFB3lp-2	*Rhizophora stylosa* (Mangrove plant, Rhizophoraceae)	Mangrove forest, Hainan, China	[73]
Cladospolide B (**89**)	228	C_12_H_20_O_4_	*Cladosporium* sp. FT-0012	Sponge	Pohnpei island, Federated State of Micronesia	[72]
			*C. herbarum* (Pers.)	*Callyspongia aerizusa* (Sponge, Callyspongiidae)	Bali Bata National Park, Indonesia,	[74]
			*Cladosporium* sp. RA07-1	*Anthogorgia ochracea* (Gorgonian, Acanthogorgiidae)	Weizhou coral reef, South China Sea	[69]
			*Cladosporium* sp. SCNU-F0001	Mangrove plant	Zhuhai Mangrove Nature, Guangdong, China	[70]
Cladospolide C (**90**)	228	C_12_H_20_O_4_	*C. cladosporioides* MCCC 3A00182	Marine sediment	Southwest Pacific Ocean	[75]
Cladospolide D (**91**)	226	C_12_H_18_O_4_	*Cladosporium* sp. FT-0012	Sponge	Pohnpei island, Federated State of Micronesia	[72]
Cladospolide E (**92**)	188	C_8_H_12_O_5_	*Cladosporium* sp. F14.	Seawater nearby mangrove stand	Kei Ling Ha Lo Wai, Sai Kung, Hong Kong, China	[76]
Pandangolide 1 (**93**)	244	C_12_H_20_O_5_	*Cladosporium* sp.	*Niphates rowi* (Sponge, Niphatidae)	Gulf of Aqaba, Israel	[77]
			*Cladosporium* sp. F14	Seawater from mangrove stand	Kei Ling Ha Lo Wai, Sai Kung, China	[60]
			*Cladosporium* sp. IFB3lp-2	*Rhizophora stylosa* (Mangrove plant, Rhizophoraceae)	Mangrove forest, Hainan, China	[73]
			*C. cladosporioides* MA-299	*Bruguiera gymnorrhiza* (Mangrove plant, Rhizophoraceae)	Hainan Island, China	[40]
Pandangolide 1a (**94**)	244	C_12_H_20_O_5_	*Cladosporium* sp.	*Niphates rowi* (Sponge, Niphatidae)	Gulf of Aqaba, Israel	[77]
			*Cladosporium* sp. IFB3lp-2	*Rhizophora stylosa* (Mangrove plant, Rhizophoraceae)	Mangrove forest, Hainan, China	[73]
Pandangolide 2 (**95**)	318	C_14_H_22_O_6_S	*C. herbarum* (Pers.)	*Callyspongia aerizusa* (Sponge, Callyspongiidae)	Bali Bata National Park, Indonesia	[74]
			*Cladosporium* sp. IFB3lp-2	*Rhizophora stylosa* (Mangrove plant, Rhizophoraceae)	Mangrove forest, Hainan, China	[73]
Pandangolide 3 (**96**)	362	C_16_H_26_O_7_S	*C. herbarum* (Pers.)	*Callyspongia aerizusa* (Sponge, Callyspongiidae)	Bali Bata National Park, Indonesia,	[74]
			*Cladosporium* sp. IFB3lp-2	*Rhizophora stylosa* (Mangrove plant, Rhizophoraceae)	Mangrove forest, Hainan, China	[73]
			*C. cladosporioides* MA-299	*Bruguiera gymnorrhiza* (Mangrove plant, Rhizophoraceae)	Hainan Island, China	[39]
			*C. oxysporum* HDN13-314	*Avicennia marina* (Mangrove plant, Acanthaceae)	Hainan, China	[78]
Pandangolide 4 (**97**)	486	C_24_H_38_O_8_S	*C. herbarum* (Pers.)	*Callyspongia aerizusa* (Sponge, Callyspongiidae)	Bali Bata National Park, Indonesia	[74]
5*R*-Hydroxyrecifeiolide (**98**)	212	C_12_H_20_O_3_	*C. cladosporioides* MA-299	*Bruguiera gymnorrhiza* (Mangrove plant, Rhizophoraceae)	Hainan Island, China	[40]
5*S*-Hydroxyrecifeiolide (**99**)	212	C_12_H_20_O_3_	*C. cladosporioides* MA-299	*Bruguiera gymnorrhiza* (Mangrove plant, Rhizophoraceae)	Hainan Island, China	[40]
Methyl 2-(((4R,6R,12R)-6-hydroxy-12-methyl-2,5-dioxooxacyclodo decan-4-yl)thio)acetate (**100**)	332	C_15_H_24_O_6_S	*Cladosporium* sp. IFB3lp-2	*Rhizophora stylosa* (Mangrove plant, Rhizophoraceae)	Mangrove forest, Hainan, China	[73]
Thiocladospolide A (**101**)	346	C_16_H_26_O_6_S	*C. cladosporioides* MA-299	*Bruguiera gymnorrhiza* (Mangrove plant, Rhizophoraceae)	Hainan Island, China	[39]
			*C. oxysporum* HDN13-314	*Avicennia marina* (Mangrove plant, Acanthaceae)	Hainan, China	[78]
Thiocladospolide B (**102**)	360	C_16_H_24_O_7_S	*C. cladosporioides* MA-299	*Bruguiera gymnorrhiza* (Mangrove plant, Rhizophoraceae)	Hainan Island, China	[39]
Thiocladospolide C (**103**)	330	C_15_H_22_O_6_S	*C. cladosporioides* MA-299	*Bruguiera gymnorrhiza* (Mangrove plant, Rhizophoraceae)	Hainan Island, China	[39]
Thiocladospolide D (**104**)	364	C_16_H_28_O_7_S	*C. cladosporioides* MA-299	*Bruguiera gymnorrhiza* (Mangrove plant, Rhizophoraceae)	Hainan Island, China	[39]
Thiocladospolide E (**105**)	306	C_14_H_26_O_5_S	*Cladosporium* sp. SCNU-F0001	Mangrove plant	Zhuhai Mangrove Nature, Guangdong, China	[70]
Thiocladospolide F (**106**)	332	C_16_H_28_O_5_S	*C. cladosporioides* MA-299	*Bruguiera gymnorrhiza* (Mangrove plant, Rhizophoraceae)	Hainan Island, China	[79]
Thiocladospolide F (**107**)	386	C_24_H_38_O_8_S	*C. oxysporum* HDN13-314	*Avicennia marina* (Mangrove plant, Acanthaceae)	Hainan, China	[78]
Thiocladospolide G (**108**)	348	C_16_H_28_O_6_S	*C. cladosporioides* MA-299	*Bruguiera gymnorrhiza* (Mangrove plant, Rhizophoraceae)	Hainan Island, China	[79]
Thiocladospolide G (**109**)	348	C_15_H_24_O_7_S	*C. oxysporum* HDN13-314	*Avicennia marina* (Mangrove plant, Acanthaceae)	Hainan, China	[78]
Thiocladospolide H (**110**)	332	C_15_H_24_O_6_S	*C. oxysporum* HDN13-314	*Avicennia marina* (Mangrove plant, Acanthaceae)	Hainan, China	[78]
Thiocladospolide I (**111**)	560	C_27_H_44_O_10_S	*C. oxysporum* HDN13-314	*Avicennia marina* (Mangrove plant, Acanthaceae)	Hainan, China	[78]
Thiocladospolide J (**112**)	558	C_27_H_42_O_10_S	*C. oxysporum* HDN13-314	*Avicennia marina* (Mangrove plant, Acanthaceae)	Hainan, China	[78]
Sporiolide A (**113**)	348	C_19_H_24_O_6_	*Cladosporium* sp. L037	*Actinotrichia fragilis* (Red alga, Galaxauraceae)	Seragaki Beach, Okinawa Island, Japan	[80]
Sporiolide B (**114**)	258	C_13_H_22_O_5_	*Cladosporium* sp. L037	*Actinotrichia fragilis* (Red alga, Galaxauraceae)	Seragaki Beach, Okinawa Island, Japan	[80]
(6*R*,12*S*)-6-Hydroxy-12-methyl-1-oxacyclododecane-2,5-dione (**115**)	228	C_12_H_20_O_4_	*Cladosporium* sp. F14	Seawater from the mangrove stand	Kei Ling Ha Lo Wai, Sai Kung, China	[60]
(3*R*,6*S*)-6-Hydroxy-12-methyl-2,5-dioxooxacyclododecan-3-yl (E)-4,11-dihydroxydodec-2-enoate (**116**)	456	C_24_H_40_O_8_	*Cladosporium* sp. IFB3lp-2	*Rhizophora stylosa* (Mangrove plant, Rhizophoraceae)	Mangrove forest, Hainan, China	[73]
Dendrodolide A (**117**)	256	C_13_H_20_O_5_	*Cladosporium* sp. RA07-1	*Anthogorgia ochracea* (Gorgonian, Acanthogorgiidae)	Weizhou coral reef, South China Sea	[69]
Dendrodolide C (**118**)	242	C_12_H_18_O_5_	*Cladosporium* sp. RA07-1	*Anthogorgia ochracea* (Gorgonian, Acanthogorgiidae)	Weizhou coral reef, South China Sea	[69]
Dendrodolide L (**119**)	228	C_12_H_20_O_4_	*Cladosporium* sp. RA07-1	*Anthogorgia ochracea* (Gorgonian, Acanthogorgiidae)	Weizhou coral reef, South China Sea	[69]
Dendrodolide M (**120**)	256	C_13_H_20_O_5_	*Cladosporium* sp. RA07-1	*Anthogorgia ochracea* (Gorgonian, Acanthogorgiidae)	Weizhou coral reef, South China Sea	[69]
Cladocladosin A (**121**)	224	C_12_H_16_O_4_	*C. cladosporioides* MA-299	*Bruguiera gymnorrhiza* (Mangrove plant, Rhizophoraceae)	Hainan Island, China	[79]
5. Butenolides and butanolides						
Cladospolide F (**122**)	230	C_12_H_22_O_4_	*Cladosporium* sp. TZP29	Unidentified soft coral	Guangzhou, China	[41]
*Ent*-cladospolide F (**123**)	230	C_14_H_24_O_5_	*C. cladosporioides* MA-299	*Bruguiera gymnorrhiza* (Mangrove plant, Rhizophoraceae)	Hainan Island, China	[40]
Cladospolide G (**124**)	272	C_14_H_24_O_5_	*C. cladosporioides* MA-299	*Bruguiera gymnorrhiza* (Mangrove plant, Rhizophoraceae)	Hainan Island, China	[40]
11-Hydroxy-γ-dodecalactone (**125**)	214	C_12_H_22_O_3_	*Cladosporium* sp. TZP29	Unidentified soft coral	Guangzhou, China	[41]
*Iso*-Cladospolide B (**126**)	228	C_12_H_20_O_4_	*C. herbarum* (Pers.)	*Callyspongia aerizusa* (Sponge, Callyspongiidae)	Bali Bata National Park, Indonesia,	[74]
			*Cladosporium* sp.	*Niphates rowi* (Sponge, Niphatidae)	Gulf of Aqaba, Israel	[77]
			*Cladosporium* sp. F14	Seawater from the mangrove stand	Kei Ling Ha Lo Wai, Sai Kung, China	[60]
			*Cladosporium* sp. IFB3lp-2	*Rhizophora stylosa* (Mangrove plant, Rhizophoraceae)	Mangrove forest, Hainan, China	[73]
			*Cladosporium* sp. RA07-1	*Anthogorgia ochracea* (Gorgonian, Acanthogorgiidae)	Weizhou coral reef, South China Sea	[70]
			*Cladosporium* sp. TZP29	Unidentified soft coral	Guangzhou, China	[41]
			*C. cladosporioides* MA-299	*Bruguiera gymnorrhiza* (Mangrove plant, Rhizophoraceae)	Hainan Island, China	[40]
			*C. oxysporum* HDN13-314	*Avicennia marina* (Mangrove plant, Acanthaceae)	Hainan, China	[78]
Cladospolide H (**127**)	210	C_12_H_18_O_3_	*C. cladosporioides* MA-299	*Bruguiera gymnorrhiza* (Mangrove plant, Rhizophoraceae)	Hainan Island, China	[40]
6. Seco-acids						
Cladospolide A II (**128**)			*Cladosporium* sp. IFB3lp-2	*Rhizophora stylosa* (Mangrove plant, Rhizophoraceae)	Mangrove forest, Hainan, China	[73]
Cladospolide E (**129**)	228	C_12_H_20_O_4_	*Cladosporium* sp. TZP29	Unidentified soft coral	Guangzhou, China	[41]
*Seco*-Patulolide A (**130**)	228	C_12_H_20_O_4_	*Cladosporium* sp. TZP29	Unidentified soft coral	Guangzhou, China	[41]
*Seco*-Patulolide C (**131**)	230	C_12_H_22_O_4_	*Cladosporium* sp. F14	Seawater from the Mangrove stand	Kei Ling Ha Lo Wai, Sai Kung, China	[60]
			*Cladosporium* sp. TZP29	Unidentified soft coral	Guangzhou, China	[41]
			*C. cladosporioides* MA-299	*Bruguiera gymnorrhiza* (Mangrove plant, Rhizophoraceae)	Hainan Island, China	[39]
*Seco*-Secopatulolide C (**132**)	230	C_12_H_22_O_4_	*C. oxysporum* HDN13-314	*Avicennia marina* (Mangrove plant, Acanthaceae)	Hainan, China	[78]
Cladosporester A (**133**)	244	C_13_H_24_O_4_	*C. cladosporioides* OUCMDZ-187	*Rhizophora stylosa* (Mangrove plant, Rhizophoraceae)	Shankou, Guangxi, China	[81]
Cladosporester B (**134**)	244	C_13_H_24_O_4_	*C. cladosporioides* OUCMDZ-187	*Rhizophora stylosa* (Mangrove plant, Rhizophoraceae)	Shankou, Guangxi, China	[81]
Cladosporacid A (**135**)	230	C_12_H_22_O_4_	*C. cladosporioides* OUCMDZ-187	*Rhizophora stylosa* (Mangrove plant, Rhizophoraceae)	Shankou, Guangxi, China	[81]
Cladosporacid B (**136**)	230	C_12_H_22_O_4_	*C. cladosporioides* OUCMDZ-187	*Rhizophora stylosa* (Mangrove plant, Rhizophoraceae)	Shankou, Guangxi, China	[81]
Cladosporacid D (**137**)	228	C_12_H_20_O_4_	*C. cladosporioides* OUCMDZ-187	*Rhizophora stylosa* (Mangrove plant, Rhizophoraceae)	Shankou, Guangxi, China	[81]
Cladosporester C (**138**)	288	C_14_H_24_O_6_	*C. cladosporioides* OUCMDZ-187	*Rhizophora stylosa* (Mangrove plant, Rhizophoraceae)	Shankou, Guangxi, China	[81]
Cladosporacid C (**139**)	230	C_12_H_22_O_4_	*C. cladosporioides* OUCMDZ-187	*Rhizophora stylosa* (Mangrove plant, Rhizophoraceae)	Shankou, Guangxi, China	[81]
Cladosporacid E (**140**)	200	C_10_H_16_O_4_	*C. cladosporioides* OUCMDZ-187	*Rhizophora stylosa* (Mangrove plant, Rhizophoraceae)	Shankou, Guangxi, China	[81]
11-Hydroxy-4,5-dioxododecanoic acid (**141**)	244	C_10_H_16_O_4_	*Cladosporium* sp. IFB3lp-2	*Rhizophora stylosa* (Mangrove plant, Rhizophoraceae)	Mangrove forest, Hainan, China	[73]
7. Tetralones (napthalenones)						
Cladosporol/Cladosporol A (**142**)	352	C_20_H_16_O_6_	*Cladosporium* sp. KcFL6′	*Kandelia candel* (Mangrove plant, Rhizophoraceae)	Daya Bay, Shenzhen city, Guangdong, China	[82]
Cladosporol C (**143**)	338	C_20_H_18_O_5_	*Cladosporium* sp. KcFL6′	*Kandelia candel* (Mangrove plant, Rhizophoraceae)	Daya Bay, Shenzhen city, Guangdong, China	[82]
			*C. cladosporioides* HDN14-342	Marine sediment	Indian Ocean, Qingdao, China	[83]
			*C. cladosporioides* EN-399	*Laurencia okamurai* (Red alga, Rhodomelaceae)	Qingdao, China	[84]
			*Cladosporium* sp. JS1-2	*Ceriops tagal* (Mangrove plant, Rhizophoraceae)	Dongzhaigang, Hainan, China	[71]
			*C. cladosporioides* MCCC 3A00182	Marine sediment	Southwest Pacific Ocean	[75]
Cladosporol D (**144**)	354	C_20_H_18_O_6_	*Cladosporium* sp. KcFL6′	*Kandelia candel* (Mangrove plant, Rhizophoraceae)	Daya Bay, Shenzhen city, Guangdong, China	[82]
Cladosporol E (**145**)	370	C_20_H_18_O_7_	*C. cladosporioides* HDN14-342	Marine sediment	Indian Ocean, Qingdao, China	[83]
			*Cladosporium* sp. JS1-2	*Ceriops tagal* (Mangrove plant, Rhizophoraceae)	Dongzhaigang, Hainan, China	[71]
Cladosporol F (**146**)	352	C_21_H_20_O_5_	*C. cladosporioides* HDN14-342	Marine sediment	Indian Ocean, Qingdao, China	[83]
			*C. cladosporioides* EN-399	*Laurencia okamurai* (Red alga, Rhodomelaceae)	Qingdao, China	[84]
Cladosporol G (**147**)	388	C_20_H_17_ClO_6_	*C. cladosporioides* HDN14-342	Marine sediment	Indian Ocean, Qingdao, China	[83]
Cladosporol G (**148**)	352	C_21_H_20_O_5_	*C. cladosporioides* EN-399	*Laurencia okamurai* (Red alga, Rhodomelaceae)	Qingdao, China	[84]
Cladosporol H (**149**)	336	C_20_H_16_O_5_	*C. cladosporioides* EN-399	*Laurencia okamurai* (Red alga, Rhodomelaceae)	Qingdao, China	[84]
Cladosporol I = Cladosperanol A (**150**)	338	C_20_H_18_O_5_	*C. cladosporioides* EN-399	*Laurencia okamurai* (Rhodomelaceae)	Qingdao, China	[84]
			*Cladosporium* sp. KFD33	Blood cockle (Bivalve mollusk, Cardiidae)	Haikou Bay, China	[85]
	338	C_20_H_18_O_5_	*C. perangustum* FS62	-	China	[86]
Cladosporol J (**151**)	338	C_20_H_18_O_5_	*C. cladosporioides* EN-399	*Laurencia okamurai* (Red alga, Rhodomelaceae)	Qingdao, China	[84]
Cladosporone A (**152**)	352	C_20_H_16_O_6_	*Cladosporium* sp. KcFL6′	*Kandelia candel* (Mangrove plant, Rhizophoraceae)	Daya Bay, Shenzhen city, Guangdong, China	[82]
Altertoxin XII (**153**)	322	C_20_H_18_O_4_	*Cladosporium* sp. KFD33	Blood cockle (Bivalve mollusk, Cardiidae)	Haikou Bay, China	[85]
Clindanone A (**154**)	394	C_22_H_18_O_7_	*C. cladosporioides* HDN14-342	Marine sediment	Indian Ocean, Qingdao, China	[83]
Clindanone B (**155**)	394	C_22_H_18_O_7_	*C. cladosporioides* HDN14-342	Marine sediment	Indian Ocean, Qingdao, China	[83]
Isosclerone = (-)-(4*R*)-Regiolone (**156**)	178	C_10_H_10_O_3_	*C. perangustm* FS62	Marine sediment	South China Sea, china	[87]
	178	C_10_H_10_O_3_	*C. cladosporioides* HDN14-342	Marine sediment	Indian Ocean, Qingdao, China	[83]
	178	C_10_H_10_O_3_	*Cladosporium* sp. JJM22	*Ceriops tagal* (Mangrove plant, Rhizophoraceae)	South China Sea, Dongzhaigang, Hainan, China	[88]
(-)-*trans*-(3*R*,4*R*)-3,4,8-Trihydroxy-6,7-dimethyl-3,4- dihydronaphthalen-1(2*H*)-one (**157**)	222	C_12_H_14_O_4_	*Cladosporium* sp. JJM22	*Ceriops tagal* (Mangrove plant, Rhizophoraceae)	South China Sea, Dongzhaigang, Hainan, China	[88]
(3*S*)-3,8-Dihydroxy-6,7-dimethyl-*α*-tetralone (**158**)	206	C_12_H_14_O_3_	*Cladosporium* sp. JJM22	*Ceriops tagal* (Mangrove plant, Rhizophoraceae)	South China Sea, Dongzhaigang, Hainan, China	[88]
(3*R*,4*R*)-3,4-Dihydro-3,4,8-trihydroxy-1(2*H*)-napthalenone (**159**)	194	C_10_H_10_O_4_	*Cladosporium* sp. JJM22	*Ceriops tagal* (Mangrove plant, Rhizophoraceae)	South China Sea, Dongzhaigang, Hainan, China	[88]
			*Cladosporium* sp. HDN17-58	Deep-sea sediment	Western Pacific Ocean, China	[89]
Aladothalen (**160**)	194	C_10_H_10_O_4_	*Cladosporium* sp. HDN17-58	Deep-sea sediment	Western Pacific Ocean, China	[89]
8. Perylenequinones						
Altertoxin VIII (**161**)	304	C_20_H_16_O_3_	*Cladosporium* sp. KFD33	Blood cockle (Bivalve mollusk, Cardiidae)	Haikou Bay, Hainan, China	[85]
Altertoxin IX (**162**)	290	C_20_H_18_O_2_	*Cladosporium* sp. KFD33	Blood cockle (Bivalve mollusk, Cardiidae)	Haikou Bay, China	[85]
Altertoxin X (**163**)	290	C_20_H_18_O_2_	*Cladosporium* sp. KFD33	Blood cockle (Bivalve mollusk, Cardiidae)	Haikou Bay, China	[85]
Altertoxin XI (**164**)	304	C_21_H_20_O_2_	*Cladosporium* sp. KFD33	Blood cockle (Bivalve mollusk, Cardiidae)	Haikou Bay, China	[85]
9. Naphthalene derivatives						
8-Methoxynaphthalen-1-ol (**165**)	174	C_11_H_10_O_2_	*Cladosporium* sp. JJM22	*Ceriops tagal* (Mangrove plant, Rhizophoraceae)	South China Sea, China	[90]
1,8-Dimethoxynaphthalene (**166**)	188	C_12_H_12_O_2_	*Cladosporium* sp. JJM22	*Ceriops tagal* (Mangrove plant, Rhizophoraceae)	South China Sea, Dongzhaigang, Hainan, China	[88]
			*Cladosporium* sp. JJM22	*Ceriops tagal* (Mangrove plant, Rhizophoraceae)	South China Sea, China	[90]
			*Cladosporium* sp. JJM22	*Ceriops tagal* (Mangrove plant, Rhizophoraceae)	South China Sea, China	[91]
4-Methoxynaphthalene-1,5-diol (**167**)	190	C_11_H_10_O_3_	*Cladosporium* sp. JJM22	*Ceriops tagal* (Mangrove plant, Rhizophoraceae)	South China Sea, China	[91]
8-Methoxynaphthalene-1,7-diol (**168**)	190	C_11_H_10_O_3_	*Cladosporium* sp. JJM22	*Ceriops tagal* (Mangrove plant, Rhizophoraceae)	South China Sea, China	[91]
Cladonaphchrom A (**169**)	350	C_22_H_22_O_4_	*Cladosporium* sp. JJM22	*Ceriops tagal* (Mangrove plant, Rhizophoraceae)	South China Sea, China	[90]
Cladonaphchrom B (**170**)	350	C_22_H_22_O_4_	*Cladosporium* sp. JJM22	*Ceriops tagal* (Mangrove plant, Rhizophoraceae)	South China Sea, China	[90]
10. Xanthones						
8-Hydroxy-6-methylxanthone-1-carboxylic acid (**171**)	270	C_15_H_10_O_5_	*C. halotolerans* GXIMD 02502	*Porites lutea* (Stony coral, Poritidae)	Weizhou Islands coral reef, Guangxi Zhuang autonomous region, China	[92]
Methyl 8-hydroxy-6-methyl-9- oxo-9H-xanthene-1-carboxylate (**172**)	284	C_16_H_12_O_5_	*C. halotolerans* GXIMD 02502	*Porites lutea* (Stony coral, Poritidae)	Weizhou Islands coral reef, Guangxi Zhuang autonomous region, China	[92]
Methyl 8-hydroxy-6- (hydroxymethyl)-9-oxo-9H-xanthene-1-carboxylate (**173**)	300	C_16_H_12_O_6_	*C. halotolerans* GXIMD 02502	*Porites lutea* (Stony coral, Poritidae)	Weizhou Islands coral reef, Guangxi Zhuang autonomous region, China	[92]
Vertixanthone (**174**)	270	C_15_H_10_O_5_	*C. halotolerans* GXIMD 02502	*Porites lutea* (Stony coral, Poritidae)	Weizhou Islands coral reef, Guangxi Zhuang autonomous region, China	[92]
8-(Methoxycarbonyl)-1-hydroxy-9-oxo-9H-xanthene-3-carboxylic acid (**175**)	314	C_16_H_10_O_7_	*C. halotolerans* GXIMD 02502	*Porites lutea* (Stony coral, Poritidae)	Weizhou Islands coral reef, Guangxi Zhuang autonomous region, China	[92]
3,8-Dihydroxy-6-methyl-9-oxo-9H-xanthene-1-Carboxylate (**176**)	300	C_16_H_12_O_6_	*C. halotolerans* GXIMD 02502	*Porites lutea* (Stony coral, Poritidae)	Weizhou Islands coral reef, Guangxi Zhuang autonomous region, China	[92]
Conioxanthone A (**177**)	316	C_16_H_12_O_7_	*C. halotolerans* GXIMD 02502	*Porites lutea* (Stony coral, Poritidae)	Weizhou Islands coral reef, Guangxi Zhuang autonomous region, China	[92]
11. Tropolones						
Malettinin A (**178**)	288	C_16_H_16_O_5_	*Cladosporium* sp. KF501	Water sample	German Wadden Sea	[93]
Malettinin B (**179**)	292	C_16_H_20_O_5_	*Cladosporium* sp. KF501	Water sample	German Wadden Sea	[93]
Malettinin C (**180**)	292	C_16_H_20_O_5_	*Cladosporium* sp. KF501	Water sample	German Wadden Sea	[93]
Malettinin E (**181**)	292	C_16_H_20_O_5_	*Cladosporium* sp. KF501	Water samples	German Wadden Sea	[93]
12. Binaphthopyrones						
Cladosporinone (**182**)	650	C_33_H_30_O_14_	C. *cladosporioides*	Sediment of a hypersaline lake El Hamra	Wadi el Natrun, Egypt	[94]
Viriditoxin (**183**)	662	C_34_H_30_O_14_	C. *cladosporioides*	Sediment of a hypersaline lake El Hamra	Wadi el Natrun, Egypt	[94]
Viriditoxin derivative 1 (**184**)	646	C_34_H_30_O_13_	C. *cladosporioides*	Sediment of a hypersaline lake El Hamra	Wadi el Natrun, Egypt	[94]
Viriditoxin derivative 2 (**185**)	646	C_34_H_30_O_13_	C. *cladosporioides*	Sediment of a hypersaline lake El Hamra	Wadi el Natrun, Egypt	[94]
13. Benzopyranes, benzopyrones, and pyrones						
(2*S*)-5-Hydroxy-2-methyl-chroman-4-one (**186**)	178	C_10_H_10_O_3_	*Cladosporium* sp. JJM22	*Ceriops tagal* (Mangrove plant, Rhizophoraceae)	South China Sea, Dongzhaigang, Hainan, China	[88]
(*R*)-5-Hydroxy-2-methylchroman-4-one (**187**)	178	C_10_H_10_O_3_	*Cladosporium* sp. JJM22	*Ceriops tagal* (Mangrove plant, Rhizophoraceae)	South China Sea, China	[90]
			*Cladosporium* sp. OUCMDZ-302	*Excoecaria agallocha* (Mangrove plant, Euphorbiaceae)	Wenchang, Hainan, China	[95]
(2*R*)-7-*O*-*α*-D-Ribofuranosyl-5-hydroxy-2-methyl chroman-4-one (**188**)	326	C_15_H_18_O_8_	*Cladosporium* sp. OUCMDZ-302	*Excoecaria agallocha* (Mangrove plant, Euphorbiaceae)	Wenchang, Hainan, China	[95]
			*Cladosporium* sp. JJM22	*Ceriops tagal* (Mangrove plant, Rhizophoraceae)	South China Sea, China	[91]
(2*S*)-7-*O*-*α*-D-Ribofuranosyl-5-hydroxy-2-methylchroman-4-one (**189**)	326	C_15_H_108_O_8_	*Cladosporium* sp. OUCMDZ-302	*Excoecaria agallocha* (Mangrove plant, Euphorbiaceae)	Wenchang, Hainan, China	[95]
(±)-5,7-Dihydroxy-2-methyl chroman-4-one (**190**)	194	C_10_H_10_O_4_	*Cladosporium* sp. OUCMDZ-302	*Excoecaria agallocha* (Mangrove plant, Euphorbiaceae)	Wenchang, Hainan, China	[95]
5-Hydroxy-2-methyl-4*H*-chromen-4-one (**191**)	176	C_10_H_8_O_3_	*Cladosporium* sp. JJM22	*Ceriops tagal* (Mangrove plant, Rhizophoraceae)	South China Sea, China	[90]
Clapone (**192**)	216	C_13_H_12_O_3_	*Cladosporium* sp. HNWSW-1	*Ceriops tagal* (Mangrove plant, Rhizophoraceae)	Dong Zhai Gang Mangrove, Hainan, China	[67]
7-*O*-*α*-D-Ribosyl-5-hydroxy-2-propylchromone (**193**)	352	C_17_H_20_O_8_	*Cladosporium* sp. OUCMDZ-302	*Excoecaria agallocha* (Mangrove plant, Euphorbiaceae)	Wenchang, Hainan, China	[95]
Coniochaetone A (**194**)	230	C_13_H_10_O_4_	*C. halotolerans* GXIMD 02502	*Porites lutea* (Stony coral, Poritidae)	Weizhou Islands coral reef, Guangxi Zhuang autonomous region, China	[92]
Coniochaetone B (**195**)	232	C_13_H_12_O_4_	*C. halotolerans* GXIMD 02502	*Porites lutea* (Stony coral, Poritidae)	Weizhou Islands coral reef, Guangxi Zhuang autonomous region, China	[92]
Coniochaetone K (**196**)	262	C_13_H_10_O_6_	*C. halotolerans* GXIMD 02502	*Porites lutea* (Stony coral, Poritidae)	Weizhou Islands coral reef, Guangxi Zhuang autonomous region, China	[92]
*α*-Diversonolic ester (**197**)	320	C_16_H_16_O_7_	*C. halotolerans* GXIMD 02502	*Porites lutea* (Poritidae)	Weizhou Islands coral reef, Guangxi Zhuang autonomous region, China	[92]
*β*-Diversonolic ester (**198**)	320	C_16_H_16_O_7_	*C. halotolerans* GXIMD 02502	*Porites lutea* (Stony coral, Poritidae)	Weizhou Islands coral reef, Guangxi Zhuang autonomous region, China	[92]
Secalonic acid D (**199**)	638	C_32_H_30_O_14_	*Cladosporium* sp. JS1-2	*Ceriops tagal* (Mangrove plant, Rhizophoraceae)	Dongzhaigang, Hainan, China	[71]
(2*S*,3*S*,4*R*)-2-Methylchroman-3,4,5-triol (**200**)	196	C_10_H_12_O_4_	*Cladosporium* sp. OUCMDZ-302	*Excoecaria agallocha* (Mangrove plant, Euphorbiaceae)	Wenchang, Hainan, China	[95]
(2*S*,4*S*)-4-Methoxy-2-methylchroman-5-ol (**201**)	194	C_11_H_14_O_3_	*Cladosporium* sp. OUCMDZ-302	*Excoecaria agallocha* (Mangrove plant, Euphorbiaceae)	Wenchang, Hainan, China	[95]
(2*R*,4*R*)-3,4-Dihydro-4-methoxy-2-methyl-2H-1-benzopyran-5-ol (**202**)	194	C_11_H_14_O_3_	*Cladosporium* sp. JJM22	*Ceriops tagal* (Mangrove plant, Rhizophoraceae)	South China Sea, China	[91]
(2*S*,4*S*)-2-methylchroman-4,5-diol (**203**)	180	C_10_H_12_O_3_	*Cladosporium* sp. OUCMDZ-302	*Excoecaria agallocha* (Mangrove plant, Euphorbiaceae)	Wenchang, Hainan, China	[95]
(2*R*,4*S*)-2,3-Dihydro-2-methyl-benzopyran-4,5-diol (**204**)	180	C_10_H_12_O_3_	*Cladosporium* sp. JJM22	*Ceriops tagal* (Mangrove plant, Rhizophoraceae)	South China Sea, China	[91]
(2R*,4R*)-3,4-Dihydro-5-methoxy-2-methyl-1(2*H*)-benzopyran-4-ol (**205**)	164	C_10_H_12_O_2_	*Cladosporium* sp. JJM22	*Ceriops tagal* (Mangrove plant, Rhizophoraceae)	South China Sea, Dongzhaigang, Hainan, China	[88]
Citrinin H1 (**206**)	428	C_24_H_28_O_7_	*Cladosporium* sp. JS1-2	*Ceriops tagal* (Mangrove plant, Rhizophoraceae)	Dongzhaigang, Hainan, China	[71]
Cladosporin C (**207**)	248	C_14_H_16_O_4_	*C. cladosporioides* SCSIO z015	Deep sea sediment	Okinawa, Japan	[36]
(*S*)-5-Hydroxy-4-methylchroman-2-one (**208**)	178	C_10_H_10_O_3_	*Cladosporium* sp. JJM22	*Ceriops tagal* (Mangrove plant, Rhizophoraceae)	South China Sea, China	[91]
(3*R*)-3-(2-Hydroxypropyl)-6,8-dihydroxy-3,4-dihydroiso-coumarin (**209**)	238	C_12_H_14_O_5_	*Cladosporium* sp. CSIO41007	*Callyspongia* sp. (Sponge, Callyspongiidae)	Xuwen, Guangdong, China	[61]
Phomasatin (**210**)	208	C_10_H_8_O_5_	*C. cladosporioides* MCCC 3A00182	Marine sediment	Southwest Pacific Ocean	[75]
14. Pyrone derivatives						
Herbarin A (**211**)	236	C_12_H_12_O_5_	*C. herbarum* (Pers.)	*Aplysina aerophoba* (Sponge, Aplysinidae)	Bali Bata National Park, Indonesia	[96]
				*Callyspongia aerizusa* (Sponge, Callyspongiidae)	Bali Bata National Park, Indonesia	[96]
Herbarin B (**212**)	210	C_10_H_10_O_5_	*C. herbarum* (Pers.)	*Aplysina aerophoba* (Sponge, Aplysinidae)	Bali Bata National Park, Indonesia	[96]
				*Callyspongia aerizusa* (Sponge, Callyspongiidae)	Bali Bata National Park, Indonesia	[96]
Citreoviridin A (**213**)	402	C_23_H_30_O_6_	*C. herbarum* (Pers.)	*Aplysina aerophoba* (Sponge, Aplysinidae)	Bali Bata National Park, Indonesia	[96]
				*Callyspongia aerizusa* (Sponge, Callyspongiidae)	Bali Bata National Park, Indonesia	[96]
Vermistatin (**214**)	328	C_18_H_16_O_6_	*Cladosporium* sp. JS1-2	*Ceriops tagal* (Mangrove plant, Rhizophoraceae)	Dongzhaigang, Hainan, China	[71]
15. Lactones, cyclohexene, and azaphilone derivatives						
(*R*)-Mevalonolactone (**215**)	130	C_8_H_10_O_3_	*Cladosporium* sp. EF424419	*Porphyra yezoensis* (Red alga, Bangiaceae)	Lianyungang, Jiangsu, China	[59]
Cladosporactone A (**216**)	196	C_10_H_12_O_4_	*C. cladosporioides* MCCC 3A00182	Marine Sediment	Southwest Pacific Ocean	[75]
Helicascolide A (**217**)	212	C_12_H_20_O_3_	*Cladosporium* sp. JJM22	*Ceriops tagal* (Mangrove plant, Rhizophoraceae)	South China Sea, China	[91]
Cladoscyclitol A (**218**)	244	C_12_H_20_O_5_	*Cladosporium* sp. JJM22	*Ceriops tagal* (Mangrove plant, Rhizophoraceae)	Dongzhaigang of Hainan Province, China	[97]
Cladoscyclitol B (**219**)	290	C_13_H_22_O_7_	*Cladosporium* sp. JJM22	*Ceriops tagal* (Mangrove plant, Rhizophoraceae)	Dongzhaigang of Hainan Province, China	[97]
Cladoscyclitol C (**220**)	230	C_12_H_22_O_4_	*Cladosporium* sp. JJM22	*Ceriops tagal* (Mangrove plant, Rhizophoraceae)	Dongzhaigang of Hainan Province, China	[97]
Cladoscyclitol D (**221**)	246	C_12_H_22_O_5_	*Cladosporium* sp. JJM22	*Ceriops tagal* (Mangrove plant, Rhizophoraceae)	Dongzhaigang of Hainan Province, China	[97]
2-Butyryl-3,5-dihydroxycyclohex-2-enone (**222**)	198	C_10_H_14_O_4_	*Cladosporium* sp. OUCMDZ-302	*Excoecaria agallocha* (Mangrove plant, Euphorbiaceae)	Wenchang, Hainan, China	[95]
Perangustol A (**223**)	210	C_11_H_14_O_4_	*C. perangustm* FS62	Marine sediment	South China Sea, China	[87]
Perangustol B (**224**)	210	C_11_H_14_O_4_	*C. perangustm* FS62	Marine sediment	South China Sea, China	[87]
Bicyclic diol (**225**)	210	C_11_H_14_O_4_	*C. perangustm* FS62	Marine sediment	South China Sea, China	[87]
16. Phenolics and other aromatic compounds						
3-Phenyl-propionic acid (**226**)	210	C_11_H_14_O_4_	*Cladosporium* sp. JJM22	*Ceriops tagal* (Rhizophoraceae)	South China Sea, China	[91]
*P*-Toluic acid (**227**)	136	C_8_H_8_O_2_	*C. cladosporioides*	Marine sponge	Argentina	[98]
L-*β*-Phenyllactic acid (**228**)	166	C_9_H_10_O_3_	*Cladosporium* sp. EF424419	*Porphyra yezoensis* (Red alga, Bangiaceae)	Lianyungang, Jiangsu, China	[59]
α-Resorcylic acid (**229**)	154	C_7_H_6_O_4_	*Cladosporium* sp. EF424419	*Porphyra yezoensis* (Red alga, Bangiaceae)	Lianyungang, Jiangsu, China	[59]
Phenylacetic acid (**230**)	136	C_8_H_8_O_2_	*Cladosporium* sp. EF424419	*Porphyra yezoensis* (Red alga, Bangiaceae)	Lianyungang, Jiangsu, China	[59]
*P*-Hydroxyphenylacetic acid (**231**)	152	C_8_H_8_O_3_	*Cladosporium* sp. EF424419	*Porphyra yezoensis* (Red alga, Bangiaceae)	Lianyungang, Jiangsu, China	[59]
Cinnamic acid (3-Phenyl-2-propenoic acid) (**232**)	148	C_9_H_8_O_2_	*Cladosporium* sp. F14	Seawater from the mangrove stand	Kei Ling Ha Lo Wai, Sai Kung, China	[60]
3-(2,3-Dihydroxy phenoxy) butanoic acid (**233**)	212	C_10_H_12_O_5_	*Cladosporium* sp. OUCMDZ-302	*Excoecaria agallocha* (Mangrove plant, Euphorbiaceae)	Wenchang, Hainan, China	[95]
*P*-Hydroxy benzoic acid methyl ester (**234**)	152	C_8_H_8_O_3_	*Cladosporium* sp. EF424419	*Porphyra yezoensis* (Red alga, Bangiaceae)	Lianyungang, Jiangsu, China	[59]
Methyl (3*S*)-3-(2,3-dihydroxy phenyloxy)butanoate (**235**)	226	C_11_H_14_O_5_	*Cladosporium* sp. OUCMDZ-302	*Excoecaria agallocha* (Mangrove plant, Euphorbiaceae)	Wenchang, Hainan, China	[95]
*P*-Hydroxyphenylethyl alcohol (**236**)	138	C_8_H_10_O_2_	*Cladosporium* sp. EF424419	*Porphyra yezoensis* (Red alga, Bangiaceae)	Lianyungang, Jiangsu Province, China	[59]
*P*-Hydroxybenzyl alcohol (**237**)	142	C_7_H_8_O_2_	*Cladosporium* sp. EF424419	*Porphyra yezoensis* (Red alga, Bangiaceae)	Lianyungang, Jiangsu Province, China	[59]
2-Phenylethanol (**238**)	122	C_8_H_10_O	*Cladosporium* sp. F14	Seawater from the mangrove stand	Kei Ling Ha Lo Wai, Sai Kung, China	[60]
4-*O*-*α*-*D*-Ribofuranose-3-hydroxymethyl-2-pentyl- phenol (**239**)	342	C_17_H_26_O_7_	*Cladosporium* sp. JJM22	*Ceriops tagal* (Mangrove plant, Rhizophoraceae)	South China Sea, Dongzhaigang, Hainan, China	[88]
4-*O*-*α*-D-Ribofuranose-2-pentyl-3-phemethylol (**240**)	326	C_17_H_26_O_6_	*Cladosporium* sp. JJM22	*Ceriops tagal* (Mangrove plant, Rhizophoraceae)	Dongzhaigang of Hainan Province, China	[97]
Clavatol (**241**)	180	C_10_H_12_O_3_	*Cladosporium* sp.MFC353-b	*Chondria crassicualis* (Red alga, Rhodomelaceae)	Yokji Island, Kyeongnam, Korea	[65]
1-(3,5-Dihydroxy-4-methylphenyl)propan-2-one (**242**)	180	C_10_H_12_O_3_	*C. perangustm* FS62	Marine sediment	South China Sea, china	[87]
*α*-Acetylorcinol (**243**)	166	C_9_H_10_O_3_	*C. perangustm* FS62	Marine sediment	South China Sea, china	[87]
1-(2,6-Dihydroxyphenyl) ethanone (**244**)	152	C_8_H_8_O_3_	*Cladosporium* sp. OUCMDZ-302	*Excoecaria agallocha* (Mangrove plant, Euphorbiaceae)	Wenchang, Hainan, China	[95]
1-(2,6-Hihydroxyphenyl)-1-butanone (**245**)	180	C_10_H_12_O_3_	*Cladosporium* sp. OUCMDZ-302	*Excoecaria agallocha* (Mangrove plant, Euphorbiaceae)	Wenchang, Hainan, China	[95]
(*R*)-3-Methoxyl-1-(2,6-dihydroxyphenyl)-butan-1-one (**246**)	210	C_11_H_14_O_4_	*Cladosporium* sp. JJM22	*Ceriops tagal* (Rhizophoraceae)	South China Sea, China	[91]
Cladosporin D (**247**)	224	C_12_H_16_O_4_	*C. cladosporioides* SCSIO z015	Deep sea sediment	Okinawa, Japan	[36]
(2*S*)-7,4′-dihydroxy-5-methoxy-8-(*γ*,*γ*-dimethylallyl)-flavanone (**248**)	354	C_21_H_22_O_5_	*Cladosporium* sp. TPU1507	Unidentified marine sponge	Manado, Indonesia	[68]
Bis(2-Ethylhexyl)phthalate (**249**)	390	C_24_H_38_O_4_	*Cladosporium* sp. F14	Seawater from the mangrove stand	Kei Ling Ha Lo Wai, Sai Kung, China	[60]
Herbaric acid (**250**)	196	C_9_H_8_O_5_	*C. herbarum* (Pers.)	*Callyspongia aerizusa* (Sponge, Callyspongiidae)	Bali Bata National Park, Indonesia	[96]
Cladosacid (**251**)	250	C_15_H_22_O_3_	*Cladosporium* sp. OUCMDZ-1635	Unidentified sponge	Xisha Islands, China	[56]
1,1′-Dioxine-2,2′-dipropionic acid (**252**)	228	C_10_H_12_O_6_	*Cladosporium* sp. JS1-2	*Ceriops tagal* (Mangrove, plant, Rhizophoraceae)	Dongzhaigang, Hainan, China	[71]
Sumiki’s acid (**253**)	142	C_6_H_6_O_4_	*C. herbarum* (Pers.)	*Callyspongia aerizusa* (Sponge, Callyspongiidae)	Bali Bata National Park, Indonesia	[73]
Acetyl Sumiki’s acid (**254**)	184	C_8_H_8_O_5_	*C. herbarum* (Pers.)	*Callyspongia aerizusa* (Sponge, Callyspongiidae)	Bali Bata National Park, Indonesia	[74]
17. Sterols and terpenes						
5*α*,8*α*-Epidioxy-24(*R*)-methyl-cholesta-6,22-diene-3-*β*-ol (**255**)	428	C_28_H_44_O_3_	*C. sphaerospermum* Penz	*Ceramium condi* (Red alga, Ceramiaceae)	Ussuriysk Bay, Japan	[99]
			*C.cladosporioides* MCCC 3A00182	Marine sediment	Southwest Pacific Ocean	[75]
5*α*,8*α*-Epidioxy-ergosta-6,22E-dien-3*β*-ol (**256**)	428	C_28_H_44_O_3_	*Cladosporium* sp. WZ-2008-0042	*Dichotella gemmacea* (Gorgonian, Ellisellidae)	Weizhou Island coral reef, South China Sea	[100]
			*C. cladosporioides* MCCC 3A00182	Marine Sediment	Southwest Pacific Ocean	[75]
5α,8α-Epidioxy-24(*R*)-methyl-cholesta-6,9(11),22-triene-3-*β*-ol (**257**)	426	C_28_H_42_O_3_	*C. sphaerospermum* Penz	*Ceramium condi* (Red alga, Ceramiaceae)	Ussuriysk Bay, Japan	[99]
5*α*,8*α*-Epidioxy-ergosta-6,9,22*E*-triene-3*β*-ol (**258**)	426	C_28_H_42_O_3_	*Cladosporium* sp. WZ-2008-0042	*Dichotella gemmacea* (Gorgonian, Ellisellidae)	Weizhou Island coral reef, South China Sea	[100]
3*β*,5*α*,6*β*- Trihydroxyergosta-7,22-diene = Cerevisterol (**259**)	430	C_28_H_46_O_3_	*Cladosporium* sp. SCSIO41007	*Callyspongia* sp. (Sponge, Callyspongiidae)	Xuwen, Guangdong, China	[61]
Ergosta-7,22*E*-diene-3*β,*5*α,*6*β*-triol (**260**)	430	C_28_H_46_O_3_	*Cladosporium* sp. WZ-2008-0042	*Dichotella gemmacea* (Gorgonian, Ellisellidae)	Weizhou Island coral reef, South China Sea	[100]
3*β*,5*α*,6*α*-Trihydroxy-(22E,24R) -ergosta-7,22-diene (**261**)	430	C_28_H_46_O_3_	*C. cladosporioides* MCCC 3A00182	Marine Sediment	Southwest Pacific Ocean	[75]
3*β,*5α-Dihydroxy-6*β*-methoxyergosta-7,22-diene (**262**)	444	C_29_H_48_O_3_	*Cladosporium* sp. WZ-2008-0042	*Dichotella gemmacea* (Gorgonian, Ellisellidae)	Weizhou Island coral reef, South China Sea	[100]
Ergosterol (**263**)	396	C_28_H_44_O	*Cladosporium* sp. WZ-2008-0042	*Dichotella gemmacea* (Gorgonian, Ellisellidae)	Weizhou Island coral reef, South China Sea	[100]
Cladosporisteroid A (**264**)	460	C_28_H_44_O_5_	*Cladosporium* sp. SCSIO41007	*Callyspongia* sp. (Sponge, Callyspongiidae)	Xuwen, Guangdong, China	[61]
3*β*,5*α*,9*α*-Trihydroxy-(22*E*,24*R*)-ergosta-7,22-diene-6-one (**265**)	444	C_28_H_44_O_4_	*Cladosporium* sp. SCSIO41007	*Callyspongia* sp. (Sponge, Callyspongiidae)	Xuwen, Guangdong, China	[61]
			*C. cladosporioides* MCCC 3A00182	Marine Sediment	Southwest Pacific Ocean	[75]
3*β*,5*α*-Dihydroxy-(22*E*,24*R*)-ergosta-7,22-diene-6-one (**266**)	428	C_28_H_44_O_3_	*C. cladosporioides* MCCC 3A00182	Marine Sediment	Southwest Pacific Ocean	[75]
Stigma-5-en-3-*O*-*β*-glucopyranoside (**267**)	576	C_35_H_60_O_6_	*Cladosporium* sp. WZ-2008-0042	*Dichotella gemmacea* (Gorgonian, Ellisellidae)	Weizhou Island coral reef, South China Sea	[100]
3*α*-Hydroxy-pregna-7-ene-6,20-dione = Cladosporisteroid B (**268**)	330	C_21_H_30_O_3_	*Cladosporium* sp. WZ-2008-0042	*Dichotella gemmacea* (Gorgonian, Ellisellidae)	Weizhou Island coral reef, South China Sea	[100]
			*Cladosporium* sp. SCSIO41007	*Callyspongia* sp. (Sponge, Callyspongiidae)	Xuwen, Guangdong, China	[61]
			*C. cladosporioides* MCCC 3A00182	Marine Sediment	Southwest Pacific Ocean	[75]
			*C. sphaerospermum* SW67	*Hydractinia echinata* (Hydroid, Hydractiniidae)	South Korea	[101]
Cladosporisteroid C (**269**)	374	C_23_H_34_O_4_	*Cladosporium* sp. SCSIO41007	*Callyspongia* sp. (Sponge, Callyspongiidae)	Xuwen, Guangdong, China	[61]
Pregn-7-dien-3,6,20-trione (**270**)	328	C_21_H_28_O_3_	*Cladosporium* sp. SCSIO41007	*Callyspongia* sp. (Sponge, Callyspongiidae)	Xuwen, Guangdong, China	[61]
18. Alcohols and aldehydes				70.43 μg/mL (EC_50_)		
Compound (**271**)	434	C_30_H_58_O	*Cladosporium* sp.	Marine sediment	San Antonio Oeste, Río Negro, Argentina	[102]
Compound (**272**)	458	C_32_H_58_O	*Cladosporium* sp.	Marine sediment	San Antonio Oeste, Río Negro, Argentina	[102]
Compound (**273**)	458	C_32_H_58_O	*Cladosporium* sp.	Marine sediment	San Antonio Oeste, Río Negro, Argentina	[102]
Compound (**274**)	458	C_32_H_58_O	*Cladosporium* sp.	Marine sediment	San Antonio Oeste, Río Negro, Argentina	[102]
Compound (**275**)	460	C_32_H_60_O	*Cladosporium* sp.	Marine sediment	San Antonio Oeste, Río Negro, Argentina	[102]
Compound (**276**)	460	C_32_H_60_O	*Cladosporium* sp.	Marine sediment	San Antonio Oeste, Río Negro, Argentina	[102]
Compound (**277**)	462	C_32_H_62_O	*Cladosporium* sp.	Marine sediment	San Antonio Oeste, Río Negro, Argentina	[102]
Compound (**278**)	462	C_32_H_62_O	*Cladosporium* sp.	Marine sediment	San Antonio Oeste, Río Negro, Argentina	[102]
Compound (**279**)	482	C_34_H_58_O	*Cladosporium* sp.	Marine sediment	San Antonio Oeste, Río Negro, Argentina	[102]
Compound (**280**)	484	C_34_H_60_O	*Cladosporium* sp.	Marine sediment	San Antonio Oeste, Río Negro, Argentina	[102]
Compound (**281**)	484	C_34_H_60_O	*Cladosporium* sp.	Marine sediment	San Antonio Oeste, Río Negro, Argentina	[102]
Compound (**282**)	484	C_34_H_60_O	*Cladosporium* sp.	Marine sediment	San Antonio Oeste, Río Negro, Argentina	[102]
Compound (**283**)	484	C_34_H_60_O	*Cladosporium* sp.	Marine sediment	San Antonio Oeste, Río Negro, Argentina	[102]
Compound (**284**)	486	C_34_H_62_O	*Cladosporium* sp.	Marine sediment	San Antonio Oeste, Río Negro, Argentina	[102]
(2*S*,3*S*,4*E*)-Hepta-4,6-diene-2,3-diol (**285**)	128	C_7_H_12_O_2_	*Cladosporium* sp. OUCMDZ-302	*Excoecaria agallocha* (Mangrove plant, Euphorbiaceae)	Wenchang, Hainan, China	[95]
(3*E*,8*E*,6*S*)-Undeca-3,8,10-trien-1,6-diol (**286**)	182	C_11_H_18_O_2_	*Cladosporium* sp. OUCMDZ-302	*Excoecaria agallocha* (Mangrove plant, Euphorbiaceae)	Wenchang, Hainan, China	[95]

**Table 2:** Biological activity of secondary metabolites isolated from *Cladosporium* species.

Compound Name	Biological Activity	Assay, Organism, or Cell Line	Biological Results	Positive Control	Ref.
Cladosin C (**3**)	Antiviral	Neuraminidase inhibition assay/Influenza A H1N1 virus	276.0 µM (IC_50_)	Ribavirin 131.0 µM (IC_50_)	[42]
Cladosin I (**8**)	Cytotoxicity	MTT/K562	4.1 µM (IC_50_)	Doxorubicin 0.3 µM (IC_50_)	[55]
	Cytotoxicity	MTT/HL-60	2.8 µM (IC_50_)	Doxorubicin 0.2 µM (IC_50_)	[55]
	Cytotoxicity	SEB/HCT-116	11.0 µM (IC_50_)	Doxorubicin 0.2 µM (IC_50_)	[55]
	Cytotoxicity	SRB/PC-3	13.0 µM (IC_50_)	Doxorubicin 1.0 µM (IC_50_)	[55]
	Cytotoxicity	SRB/SH-SY5Y	12.0 µM (IC_50_)	Doxorubicin 0.1 µM (IC_50_)	[55]
	Cytotoxicity	SRB/MGC-803	19.0 µM (IC_50_)	Doxorubicin 0.2 µM (IC_50_)	[55]
Cladosin K (**10**)	Cytotoxicity	MTT/K562	5.9 µM (IC_50_)	Doxorubicin 0.3 µM (IC_50_)	[55]
	Cytotoxicity	MTT/HL-60	7.5 µM (IC_50_)	Doxorubicin 0.2 µM (IC_50_)	[55]
	Cytotoxicity	SEB/HCT-116	14.0 µM (IC_50_)	Doxorubicin 0.2 µM (IC_50_)	[55]
	Cytotoxicity	SRB/PC-3	18.0 µM (IC_50_)	Doxorubicin 1.0 µM (IC_50_)	[55]
Cladosporicin A (**12**)	Cytotoxicity	SRB/Bt549	70.88 µM (IC_50_)	Etoposide 1.82 µM (IC_50_)	[38]
	Cytotoxicity	SRB/HCC70	74.48 µM (IC_50_)	Etoposide 1.76 µM (IC_50_)	[38]
	Cytotoxicity	SRB/MDA-MB-231	75.54 µM (IC_50_)	Etoposide 2.27 µM (IC_50_)	[38]
	Cytotoxicity	SRB/MDA-MB-468	79.36 µM (IC_50_)	Etoposide 2.08 µM (IC_50_)	[38]
Cladodionen (**13**)	Cytotoxicity	MTT/K562	4.5 µM (IC_50_)	Doxorubicin 0.3 µM (IC_50_)	[55]
	Cytotoxicity	MTT/HL-60	6.6 µM (IC_50_)	Doxorubicin 0.2 µM (IC_50_)	[55]
	Cytotoxicity	SRB/HCT-116	12.0 µM (IC_50_)	Doxorubicin 0.2 µM (IC_50_)	[55]
	Cytotoxicity	SRB/PC-3	11.0 µM (IC_50_)	Doxorubicin 1.0 µM (IC_50_)	[55]
	Cytotoxicity	SRB/SH-SY5Y	15.0 µM (IC_50_)	Doxorubicin 0.1 µM (IC_50_)	[55]
	Cytotoxicity	SRB/MGC-803	22.0 µM (IC_50_)	Doxorubicin 0.2 µM (IC_50_)	[55]
	Cytotoxicity	MTT/MCF-7	18.7 µM (IC_50_)	Adriamycin 0.67 µM (IC_50_)	[56]
	Cytotoxicity	MTT/HeLa	19.1 µM (IC_50_)	Adriamycin 0.32 µM (IC_50_)	[56]
	Cytotoxicity	CCK-8/HCT-116	17.9 µM (IC_50_)	Adriamycin 0.21 µM (IC_50_)	[56]
	Cytotoxicity	MTT/HL-60	9.0 µM (IC_50_)	Adriamycin 0.02 µM (IC_50_)	[56]
Cladosporiumin I (**29**)	Cytotoxicity	SRB/Bt549	76.18 µM (IC_50_)	Etoposide 1.82 µM (IC_50_)	[38]
	Cytotoxicity	SRB/HCC70	85.29 µM (IC_50_)	Etoposide 1.76 µM (IC_50_)	[38]
	Cytotoxicity	SRB/MDA-MB-231	82.37 µM (IC_50_)	Etoposide 2.27 µM (IC_50_)	[38]
	Cytotoxicity	SRB/MDA-MB-468	81.44 µM (IC_50_)	Etoposide 2.08 µM (IC_50_)	[38]
Cladosporiumin J (**30**)	Cytotoxicity	SRB/Bt549	78.96 µM (IC_50_)	Etoposide 1.82 µM (IC_50_)	[38]
	Cytotoxicity	SRB/HCC70	76.41 µM (IC_50_)	Etoposide 1.76 µM (IC_50_)	[38]
	Cytotoxicity	SRB/MDA-MB-231	79.27 µM (IC_50_)	Etoposide 2.27 µM (IC_50_)	[38]
	Cytotoxicity	SRB/MDA-MB-468	74.64 µM (IC_50_)	Etoposide 2.08 µM (IC_50_)	[38]
Cyclo-(Val-Pro) (**32**)	Insecticidal	Inhibitinon 50%/*B. amphitrite*	37.82 μg/mL (EC_50_)	FSW with DMSO	[60]
		Lethality 50%/*B. amphitrite*	>200 μg/mL (LC_50_)	FSW with DMSO	[60]
		Inhibitinon 50%/*B. neritina*	>200 μg/mL (EC_50_)	FSW with DMSO	[60]
		Lethality 50%/*B. neritina*	>200 μg/mL (LC_50_)	FSW with DMSO	[60]
Cyclo-(Val-Pro) (**32**)	Antimicrobial	Serial dilution/*L. hongkongensis*	80 μg/mL (MIC)	Streptomycin 250 μg/mL (MIC) Penicillin 0.25 μg/mL (MIC)	[60]
Cyclo-(Phe-Pro) (**33**)	Insecticidal	Inhibitinon 50%/*B. amphitrite*	68.57 μg/mL (EC_50_)	FSW with DMSO	[60]
		Lethality 50%/*B. amphitrite*	115.04 μg/mL (LC_50_)	FSW with DMSO	[60]
		Inhibitinon 50%/*B. neritina*	70.43 μg/mL (EC_50_)	FSW with DMSO	[60]
		Lethality 50%/*B. neritina*	>200 μg/mL (LC_50_)	FSW with DMSO	[60]
Cyclo-(Phe-Pro) (**33**)	Antimicrobial	Serial dilution/*L. hongkongensis*	200 μg/mL (MIC)	Streptomycin 1.0 250 μg/mL (MIC)	[60]
		Serial dilution/*M. luteus*	200 μg/mL (MIC)	Streptomycin 250 μg/mL (MIC) Penicillin 0.5 μg/mL (MIC)	[60]
		Serial dilution/*Ruegeria* sp.	100 μg/mL (MIC)	Streptomycin 500 μg/mL (MIC) Penicillin 0.25 μg/mL (MIC)	[60]
Glyantrypine (**42**)	Antiviral	CPE inhibition assay/Influenza A H1N1 virus	150 µM (IC_50_)	Ribavirin 87.0 µM (IC_50_)	[64]
3-Hydroxyglyantrypine (**43**)	Antiviral	CPE inhibition assay/Influenza A H1N1 virus	110 µM (IC_50_)	Ribavirin 87.0 µM (IC_50_)	[64]
14R-Oxoglyantrypine (**44**)	Antiviral	CPE inhibition assay/Influenza A H1N1 virus	130 µM (IC_50_)	Ribavirin 87.0 µM (IC_50_)	[64]
14S-Oxoglyantrypine (**45**)	Antiviral	CPE inhibition assay/Influenza A H1N1 virus	85 µM (IC_50_)	Ribavirin 87.0 µM (IC_50_)	[64]
Cladoquinazoline (**47**)	Antiviral	CPE inhibition assay/Influenza A H1N1 virus	150 µM (IC_50_)	Ribavirin 87.0 µM (IC_50_)	[64]
*Epi*-Cladoquinazoline (**48**)	Antiviral	CPE inhibition assay/Influenza A H1N1 virus	140 µM (IC50)	Ribavirin 87.0 µM (IC_50_)	[64]
Norquinadoline A (**49**)	Antiviral	CPE inhibition assay/Influenza A H1N1 virus	82 µM (IC_50_)	Ribavirin 87.0 µM (IC_50_)	[64]
Quinadoline A (**50**)	Antiviral	CPE inhibition assay/Influenza A H1N1 virus	130 µM (IC_50_)	Ribavirin 87.0 µM (IC_50_)	[64]
Deoxynortryptoquivaline (**51**)	Antiviral	CPE inhibition assay/Influenza A H1N1 virus	87 µM (IC_50_)	Ribavirin 87.0 µM (IC_50_)	[64]
Deoxytryptoquivaline (**52**)	Antiviral	CPE inhibition assay/Influenza A H1N1 virus	85 µM (IC_50_)	Ribavirin 87.0 µM (IC_50_)	[64]
Tryptoquivaline (**53**)	Antiviral	CPE inhibition assay/Influenza A H1N1 virus	89 µM (IC_50_)	Ribavirin 87.0 µM (IC_50_)	[64]
CS-C (**54**)	Antiviral	CPE inhibition assay/Influenza A H1N1 virus	140 µM (IC_50_)	Ribavirin 87.0 µM (IC_50_)	[64]
Quinadoline B (**55**)	Antiviral	CPE inhibition assay/Influenza A H1N1 virus	82 µM (IC_50_)	Ribavirin 87.0 µM (IC_50_)	[64]
Quinolactacin A2 (**58**)	Cytotoxicity	MTT/HepG-2	96.54 µM (IC_50_)	Curcumin 61.38 µM (IC_50_)	[66]
		MTT/HL-60	54.47 µM (IC_50_)	Curcumin 13.78 µM (IC_50_)	[66]
		MTT/MCF-7	94.49 µM (IC_50_)	Curcumin 20.68 µM (IC_50_)	[66]
		MTT/LNCap	45.71 µM (IC_50_)	Curcumin 6.15 µM (IC_50_)	[66]
	Anti-malarial	Flow cytometry/SYBR Green I fluorescence/*P. falciparum* chloroquine sensitive (3D7)	24.8 µM (EC_50_)	Artesunate 0.074 µM (EC_50_)	[66]
Citrinadin A (**68**)	Cytotoxicity	MTT/HepG-2	82.15 µM (IC_50_)	Curcumin 61.38 µM (IC_50_)	[66]
		MTT/HL-60	57.23 µM (IC_50_)	Curcumin 13.78 µM (IC_50_)	[66]
		MTT/MCF-7	66.07 µM (IC_50_)	Curcumin 20.68 µM (IC_50_)	[66]
		MTT/LNCap	41.42 µM (IC_50_)	Curcumin 6.15 µM (IC_50_)	[66]
	Anti-malarial	Flow cytometry/SYBR Green I fluorescence/*P. falciparum* chloroquine sensitive (3D7)	>25.0 µM (EC_50_)	Artesunate 0.074 µM (EC_50_)	[66]
Butrecitrinadin (**70**)	Cytotoxicity	MTT/HepG-2	78.57 µM (IC_50_)	Curcumin 61.38 µM (IC_50_)	[66]
		MTT/HL-60	60.31 µM (IC_50_)	Curcumin 13.78 µM (IC_50_)	[66]
		MTT/MCF-7	51.32 µM (IC_50_)	Curcumin 20.68 µM (IC_50_)	[66]
		MTT/LNCap	32.94 µM (IC_50_)	Curcumin 6.15 µM (IC_50_)	[66]
	Anti-malarial	Flow cytometry/SYBR Green I fluorescence/*P. falciparum* chloroquine sensitive (3D7)	>25.0 µM (EC_50_)	Artesunate 0.074 µM (EC_50_)	[66]
Cladosporitin B (**74**)	Cytotoxicity	MTT/BEL-7042	29.4 µM (IC_50_)	Adriamycin 11.9 µM (IC_50_)	[67]
	Cytotoxicity	MTT/K562	25.6 µM (IC_50_)	Adriamycin 14.2 µM (IC_50_)	[67]
	Cytotoxicity	MTT/SGC-7901	41.7 µM (IC_50_)	Adriamycin 6.66 µM (IC_50_)	[67]
Talaroconvolutin A (**76**)	Cytotoxicity	MTT/HeLa	14.9 µM (IC_50_)	Adriamycin 11.5 µM (IC_50_)	[67]
	Cytotoxicity	MTT/BEL-7042	26.7 µM (IC_50_)	Adriamycin 11.9 µM (IC_50_)	[67]
Talaroconvolutin A (**76**)	α-Glucosidase inhibitory	Glucose oxidase method	78.2 µM (IC_50_)	Acarbose 275.7 µM (IC_50_)	[67]
Cladosporamide A (**77**)	Protein tyrosine phosphatase 1B inhibitory	PTP1B/Spectrophotometry	48.0 µM (IC_50_)	Oleanolic acid 0.9 µM (IC_50_)	[68]
		TCPTP/Spectrophotometry	54.0 µM (IC_50_)	Oleanolic acid 0.8 µM (IC_50_)	[68]
Cladosporilactam A (**78**)	Cytotoxicity	MTT/HeLa	0.76 µM (IC_50_)	Adriamycin	[69]
		MTT/HT-29	2.48 µM (IC_50_)	Adriamycin	[69]
		SRB/P388	1.35 µM (IC_50_)	Adriamycin	[69]
		SRB/A549	3.11 µM (IC_50_)	Adriamycin	[69]
2-Methylacetate-3,5,6-trimethylpyrazine (**84**)	Insecticidal	CM/Helicoverpa armigera Hubner larvae	100.0 μg/mL (IC_50_)	Azadirachtin 25.0 μg/mL (IC_50_)	[71]
	Antimicrobial	Microplate assay/*S. aureus*	12.5 μg/mL (MIC)	Ciprofloxacin 0.39 μg/mL (MIC)	[71]
Cytochalasin D (**85**)	Antimicrobial	Microplate assay/*S. aureus*	25.0 μg/mL (MIC)	Ciprofloxacin 0.39 μg/mL (MIC)	[71]
Pandangolide 3 (**96**)	Antimicrobial	Microplate assay/*C. glecosporioides*	2.0 µg/mL (MIC)	Amphotericin B 0.5 µg/mL (MIC)	[39]
		Microplate assay/*B. sorokiniana*	8.0 µg/mL (MIC)	Amphotericin B 0.5 µg/mL (MIC)	[39]
Thiocladospolide A (**101**)	Antimicrobial	Microplate assay/*E. tarda*	1.0 µg/mL (MIC)	Chloramphenicol 0.5 µg/mL (MIC)	[39]
		Microplate assay/*E. ictarda*	8.0 µg/mL (MIC)	Chloramphenicol 0.5 µg/mL (MIC)	[39]
		Microplate assay/*C. glecosporioides*	2.0 µg/mL (MIC)	Amphotericin B 0.5 µg/mL (MIC)	[39]
Thiocladospolide B (**102**)	Antimicrobial	Microplate assay/*C. glecosporioides*	2.0 µg/mL (MIC)	Amphotericin B 0.5 µg/mL (MIC)	[39]
		Microplate assay/*P. piricola* Nose	32.0 µg/mL (MIC)	Amphotericin B 2.0 µg/mL (MIC)	[39]
		Microplate assay/*F. oxysporum* f. sp. *cucumerinum*	1.0 µg/mL (MIC)	Amphotericin B 0.5 µg/mL (MIC)	[39]
Thiocladospolide C (**103**)	Antimicrobial	Microplate assay/*C. glecosporioides*	1.0 µg/mL (MIC)	Amphotericin B 0.5 µg/mL (MIC)	[39]
		Microplate assay/*P. piricola* Nose	32.0 µg/mL (MIC)	Amphotericin B 2.0 µg/mL (MIC)	[39]
		Microplate assay/*F. oxysporum* f. sp. *cucumerinum*	32.0 µg/mL (MIC)	Amphotericin B 0.5 µg/mL (MIC)	[39]
Thiocladospolide D (**104**)	Antimicrobial	Microplate assay/*E. ictarda*	1.0 µg/mL (MIC)	Chloramphenicol 0.5 µg/mL (MIC)	[39]
		Microplate assay/*C*. *glecosporioides*	1.0 µg/mL (MIC)	Amphotericin B 0.5 µg/mL (MIC)	[39]
		Microplate assay/*P. piricola* Nose	32.0 µg/mL (MIC)	Amphotericin B 2.0 µg/mL (MIC)	[39]
		Microplate assay/*F. oxysporum* f. sp. *cucumerinum*	1.0 µg/mL (MIC)	Amphotericin B 0.5 µg/mL (MIC)	[39]
Thiocladospolide F (**106**)	Antimicrobial	Microplate assay/*E. tarda*	2.0 µg/mL (MIC)	Chloramphenicol 0.5 µg/mL (MIC)	[79]
	Antimicrobial	Microplate assay/*H. maydis*	4.0 µg/mL (MIC)	Amphotericin B 0.5 µg/mL (MIC)	[79]
Thiocladospolide G (**108**)	Antimicrobial	Microplate assay/*E. tarda*	2.0 µg/mL (MIC)	Chloramphenicol 0.5 µg/mL (MIC)	[79]
Thiocladospolide G (**109**)	Antimicrobial	Microplate assay/*E. tarda*	4.0 μg/mL (MIC)	Chloramphenicol 1.0 μg/mL (MIC)	[78]
Thiocladospolide H (**110**)	Antimicrobial	Microplate assay/*E. ictarda*	8.0 μg/mL (MIC)	Chloramphenicol 1.0 μg/mL (MIC)	[78]
Sporiolide A (**113**)	Cytotoxicity	MTT/L1210	0.13 µM (IC_50_)	-	[80]
Sporiolide B (**114**)	Cytotoxicity	MTT/L1210	0.81 µM (IC_50_)	-	[80]
Dendrodolide A (**117**)	Antimicrobial	Broth dilution assay/*B. cereus*	12.5 μM (MIC)	Ciprofloxacin 1.56 μM (MIC)	[69]
		Broth dilution assay/*T. halophilus*	3.13 μM (MIC)	Ciprofloxacin 1.56 μM (MIC)	[69]
		Broth dilution assay/*S. epidermidis*	6.25 μM (MIC)	Ciprofloxacin 0.78 μM (MIC)	[69]
		Broth dilution assay/*S. aureus*	6.25 μM (MIC)	Ciprofloxacin 0.39 μM (MIC)	[69]
		Broth dilution assay/*E. coli*	12.5 μM (MIC)	Ciprofloxacin 1.56 μM (MIC)	[69]
		Broth dilution assay/*P. putida*	12.5 μM (MIC)	Ciprofloxacin 0.39 μM (MIC)	[69]
		Broth dilution assay/*N. brasiliensis*	6.25 μM (MIC)	Ciprofloxacin 0.78 μM (MIC)	[69]
		Broth dilution assay/*V. parahaemolyticus*	12.5 μM (MIC)	Ciprofloxacin 1.56 μM (MIC)	[69]
Dendrodolide C (**118**)	Antimicrobial	Broth dilution assay/*B. cereus*	25.0 μM (MIC)	Ciprofloxacin 1.56 μM (MIC)	[69]
		Broth dilution assay/*T. halophilus*	3.13 μM (MIC)	Ciprofloxacin 1.56 μM (MIC)	[69]
		Broth dilution assay/*S. epidermidis*	25.0 μM (MIC)	Ciprofloxacin 0.78 μM (MIC)	[69]
		Broth dilution assay/*S. aureus*	25.0 μM (MIC)	Ciprofloxacin 0.39 μM (MIC)	[69]
		Broth dilution assay/*E. coli*	12.5 μM (MIC)	Ciprofloxacin 1.56 μM (MIC)	[69]
		Broth dilution assay/*P. putida*	25.0 μM (MIC)	Ciprofloxacin 0.39 μM (MIC)	[69]
		Broth dilution assay/*N. brasiliensis*	12.5 μM (MIC)	Ciprofloxacin 0.78 μM (MIC)	[69]
		Broth dilution assay/*V. parahaemolyticus*	25.0 μM (MIC)	Ciprofloxacin 1.56 μM (MIC)	[69]
Dendrodolide M (**120**)	Antimicrobial	Broth dilution assay/*B. cereus*	6.25 μM (MIC)	Ciprofloxacin 1.56 μM (MIC)	[69]
		Broth dilution assay/*T. halophilus*	25.0 μM (MIC)	Ciprofloxacin 1.56 μM (MIC)	[69]
		Broth dilution assay/*S. epidermidis*	25.0 μM (MIC)	Ciprofloxacin 0.78 μM (MIC)	[69]
		Broth dilution assay/*S. aureus*	12.5 μM (MIC)	Ciprofloxacin 0.39 μM (MIC)	[69]
Dendrodolide C (**118**)	Antimicrobial	Broth dilution assay/*B. cereus*	25.0 μM (MIC)	Ciprofloxacin 1.56 μM (MIC)	[69]
		Broth dilution assay/*E. col*i	25.0 μM (MIC)	Ciprofloxacin 1.56 μM (MIC)	[69]
		Broth dilution assay/*P. putida*	6.25 μM (MIC)	Ciprofloxacin 0.39 μM (MIC)	[69]
		Broth dilution assay/*N. brasiliensis*	25.0 μM (MIC)	Ciprofloxacin 0.78 μM (MIC)	[69]
		Broth dilution assay/*V. parahaemolyticus*	25.0 μM (MIC)	Ciprofloxacin 1.56 μM (MIC)	[69]
Cladocladosin A (**121**)	Antimicrobial	Microplate assay/*E. tarda*	1.0 µg/mL (MIC)	Chloramphenicol 0.5 µg/mL (MIC)	[79]
	Antimicrobial	Microplate assay/*P. aeruginosa*	4.0 µg/mL (MIC)	Chloramphenicol 2.0 µg/mL (MIC)	[79]
*Ent*-cladospolide F (**123**)	AchE inhibitory	Modified Ellman’s enzyme/Immunosorbent assay	40.26 µM (IC_50_)	Tacrine 0.5 µM (IC_50_)	[40]
Iso-cladospolide B (**126**)	Antimicrobial	Broth dilution assay/*B. cereus*	6.25 μM (MIC)	Ciprofloxacin 1.56 μM (MIC)	[69]
		Broth dilution assay/*T. halophilus*	6.25 μM (MIC)	Ciprofloxacin 1.56 μM (MIC)	[69]
		Broth dilution assay/*S. epidermidis*	25.0 μM (MIC)	Ciprofloxacin 0.78 μM (MIC)	[69]
		Broth dilution assay/*S. aureus*	25.0 μM (MIC)	Ciprofloxacin 0.39 μM (MIC)	[69]
		Broth dilution assay/*E. coli*	25.0 μM (MIC)	Ciprofloxacin 1.56 μM (MIC)	[69]
		Broth dilution assay/*P. putida*	6.25 μM (MIC)	Ciprofloxacin 0.39 μM (MIC)	[69]
		Broth dilution assay/*N. brasiliensis*	12.5 μM (MIC)	Ciprofloxacin 0.78 μM (MIC)	[69]
		Broth dilution assay/*V. parahaemolyticus*	25.0 μM (MIC)	Ciprofloxacin 1.56 μM (MIC)	[69]
		Microplate assay/*C. mandshurica* Miura	8.0 μg/mL (MIC)	Nystatin 1.0 μg/mL (MIC)	[78]
Cladosporol C (**143**)	Cytotoxicity	Trypan blue-cell viability assay/K562	˃30.0 µM (IC_50_)	Trichostatin A 0.24 µM (IC_50_)	[82]
		Trypan blue-cell viability assay/A549	33.9 µM (IC_50_)	Trichostatin A 0.05 µM (IC_50_)	[82]
		Trypan blue-cell viability assay/Huh-7	˃30.0 µM (IC_50_)	Trichostatin A 0.08 µM (IC_50_)	[82]
		Trypan blue-cell viability assay/H1975	45.6 µM (IC_50_)	Trichostatin A 0.09 µM (IC_50_)	[82]
		Trypan blue-cell viability assay/MCF-7	˃30.0 µM (IC_50_)	Trichostatin A 0.78 µM (IC_50_)	[82]
		Trypan blue-cell viability assay/U937	˃30.0 µM (IC_50_)	Trichostatin A 0.06 µM (IC_50_)	[82]
		Trypan blue-cell viability assay/BGC823	˃30.0 µM (IC_50_)	Trichostatin A 0.09 µM (IC_50_)	[82]
		Trypan blue-cell viability assay/HL-60	72.5 µM (IC_50_)	Trichostatin A 0.09 µM (IC_50_)	[82]
		Trypan blue-cell viability assay/A549	˃30.0 µM (IC_50_)	Trichostatin A 0.11 µM (IC_50_)	[82]
		Trypan blue-cell viability assay/MOLT-4	14.4 µM (IC_50_)	Trichostatin A 0.03 µM (IC_50_)	[82]
		MTT/A549	14.0 µM (IC_50_)	Cisplatin 1.3 µM (IC_50_)	[84]
		MTT/HeLa	4.0 µM (IC_50_)	Paclitaxel 4.9 µM (IC_50_)	[84]
	Antimicrobial	Microplate assay/*E. coli*	8.0 μg/mL (MIC)	Chloramphenicol 0.025 μg/mL (MIC)	[84]
		Microplate assay/*M. luteus*	32.0 μg/mL (MIC)	Chloramphenicol 0.5 μg/mL (MIC)	[84]
		Microplate assay/*V. harveyi*	16.0 μg/mL (MIC)	Chloramphenicol 2.0 μg/mL (MIC)	[84]
		Microplate assay/*S. aureus*	6.25 μg/mL (MIC)	Ciprofloxacin 0.39 μg/mL (MIC)	[71]
		Microplate assay/*M. luteus*	12.5 μg/mL (MIC)	Ciprofloxacin 0.39 μg/mL (MIC)	[71]
Cladosporol D (**144**)	Anti-inflammatory	Spectrophotometry/Anti-COX-2/PGF_2_*_α_* inhibition	60.2 µM (IC_50_)	Indomethacin 18.3 µM (IC_50_) NS-398 1.0 µM (IC_50_)	[82]
Cladosporol E (**145**)	Insecticidal	Measuring the corrected mortality (CM)	150.0 μg/mL (IC_50_)	Azadirachtin 25.0 μg/mL (IC_50_)	[71]
	Antimicrobial	Microplate assay/*S. aureus*	1.56 μg/mL (MIC)	Ciprofloxacin 0.39 μg/mL (MIC)	[71]
		Microplate assay/*M. luteus*	12.5 μg/mL (MIC)	Ciprofloxacin 0.39 μg/mL (MIC)	[71]
Cladosporol F (**146**)	Cytotoxicity	MTT/K562	23.0 µM (IC_50_)	Doxorubicin 0.6 µM (IC_50_)	[83]
		SRB/HeLa	13.8 µM (IC_50_)	Doxorubicin 0.5 µM (IC_50_)	[83]
		SRB/HCT-116	23.0 µM (IC_50_)	Doxorubicin 0.2 µM (IC_50_)	[83]
		MTT/A549	15.0 µM (IC_50_)	Cisplatin 1.3 µM (IC_50_)	[84]
		MTT/HeLa	10.0 µM (IC_50_)	Paclitaxel 4.9 µM (IC_50_)	[84]
	Antimicrobial	Microplate assay/*E. coli*	32.0 μg/mL (MIC)	Chloramphenicol 0.025 μg/mL (MIC)	[84]
		Microplate assay/*M. luteus*	64.0 μg/mL (MIC)	chloramphenicol 0.5 μg/mL (MIC)	[84]
		Microplate assay/*V. harveyi*	32.0 μg/mL (MIC)	Chloramphenicol 2.0 μg/mL (MIC)	[84]
Cladosporol G (**147**)	Cytotoxicity	MTT/K562	8.8 µM (IC_50_)	Doxorubicin 0.6 µM (IC_50_)	[83]
		SRB/HeLa	3.9 µM (IC_50_)	Doxorubicin 0.5 µM (IC50)	[83]
		SRB/HCT-116	19.4 µM (IC_50_)	Doxorubicin 0.2 µM (IC_50_)	[83]
Cladosporol G (**148**)	Cytotoxicity	MTT/A549	13.0 µM (IC_50_)	Cisplatin 1.3 µM (IC_50_)	[84]
		MTT/H446	11.0 µM (IC_50_)	Adriamycin 4.0 µM (IC_50_)	[84]
		MTT/Huh7	10.0 µM (IC_50_)	Fluorouracil 6.2 µM (IC_50_)	[84]
		MTT/L02	11.0 µM (IC_50_)	Cisplatin 13.0 µM (IC_50_)	[84]
		MTT/LM3	14.0 µM (IC_50_)	Cisplatin 9.1 µM (IC_50_)	[84]
		MTT/SW1990	15.0 µM (IC_50_)	Gemcitabine 2.2 µM (IC_50_)	[84]
	Antimicrobial	Microplate assay/*E. coli*	64.0 μg/mL (MIC)	Chloramphenicol 0.025 μg/mL (MIC)	[84]
		Microplate assay/*M. luteus*	128.0 μg/mL (MIC)	Chloramphenicol 0.5 μg/mL (MIC)	[84]
		Microplate assay/*V. harveyi*	64.0 μg/mL (MIC)	Chloramphenicol 2.0 μg/mL (MIC)	[84]
Cladosporol H (**149**)	Cytotoxicity	MTT/A549	5.0 µM (IC_50_)	Cisplatin 1.3 µM (IC_50_)	[84]
		MTT/H446	10.0 µM (IC_50_)	Adriamycin 4.0 µM (IC_50_)	[84]
		MTT/Huh7	1.0 µM (IC_50_)	Fluorouracil 6.2 µM (IC_50_)	[84]
		MTT/LM3	4.1 µM (IC_50_)	Cisplatin 9.1 µM (IC_50_)	[84]
		MTT/MCF-7	10.0 µM (IC_50_)	Paclitaxel 1.8 µM (IC_50_)	[84]
		MTT/SW1990	15.0 µM (IC_50_)	Gemcitabine 2.2 µM (IC_50_)	[84]
	Antimicrobial	Microplate assay/*E. coli*	32.0 μg/mL (MIC)	Chloramphenicol 0.025 μg/mL (MIC)	[84]
		Microplate assay/*M. luteus*	64.0 μg/mL (MIC)	Chloramphenicol 0.5 μg/mL (MIC)	[84]
		Microplate assay/*V. harveyi*	4.0 μg/mL (MIC)	Chloramphenicol 2.0 μg/mL (MIC)	[84]
Cladosporol I (**150**)	Cytotoxicity	MTT/HeLa	10.8 µM (IC_50_)	Paclitaxel 4.9 µM (IC_50_)	[84]
	Antimicrobial	Microplate assay/*E. coli*	64.0 μg/mL (MIC)	Chloramphenicol 0.025 μg/mL (MIC)	[84]
		Microplate assay/*M. luteus*	64.0 μg/mL (MIC)	Chloramphenicol 0.5 μg/mL (MIC)	[84]
		Microplate assay/*V. harveyi*	16.0 μg/mL (MIC)	Chloramphenicol 2.0 μg/mL (MIC)	[84]
Cladosporol J (**151**)		MTT/A549	15.0 µM (IC_50_)	Cisplatin 1.3 µM (IC_50_)	[84]
		MTT/H446	11.0 µM (IC_50_)	Adriamycin 4.0 µM (IC_50_)	[84]
		MTT/HeLa	15.0 µM (IC_50_)	Paclitaxel 4.9 µM (IC_50_)	[84]
		MTT/Huh7	20.0 µM (IC_50_)	Fluorouracil 6.2 µM (IC_50_)	[84]
		MTT/MCF-7	12.0 µM (IC_50_)	Paclitaxel 1.8 µM (IC_50_)	[84]
	Antimicrobial	Microplate assay/*E. coli*	16.0 μg/mL (MIC)	Chloramphenicol 0.025 μg/mL (MIC)	[84]
		Microplate assay/*M. luteus*	64.0 μg/mL (MIC)	Chloramphenicol 0.5 μg/mL (MIC)	[84]
		Microplate assay/*V. harveyi*	32.0 μg/mL (MIC)	Chloramphenicol 2.0 μg/mL (MIC)	[84]
Cladosporone A (**152**)	Cytotoxicity	Trypan blue-cell viability assay/K562	14.3 µM (IC_50_)	Trichostatin A 0.24 µM (IC_50_)	[82]
		Trypan blue-cell viability assay/A549	15.7 µM (IC_50_)	Trichostatin A 0.05 µM (IC_50_)	[82]
		Trypan blue-cell viability assay/Huh-7	29.9 µM (IC_50_)	Trichostatin A 0.08 µM (IC_50_)	[82]
		Trypan blue-cell viability assay/H1975	40.6 µM (IC_50_)	Trichostatin A 0.09 µM (IC_50_)	[82]
		Trypan blue-cell viability assay/MCF-7	21.3 µM (IC_50_)	Trichostatin A 0.78 µM (IC_50_)	[82]
		Trypan blue-cell viability assay/U937	10.5 µM (IC_50_)	Trichostatin A 0.06 µM (IC_50_)	[82]
		Trypan blue-cell viability assay/BGC823	17.0 µM (IC_50_)	Trichostatin A 0.09 µM (IC_50_)	[82]
		Trypan blue-cell viability assay/HL-60	10.1 µM (IC_50_)	Trichostatin A 0.09 µM (IC_50_)	[82]
		Trypan blue-cell viability assay/A549	53.7 µM (IC_50_)	Trichostatin A 0.11 µM (IC_50_)	[82]
		Trypan blue-cell viability assay/MOLT-4	14.6 µM (IC_50_)	Trichostatin A 0.03 µM (IC_50_)	[82]
	Anti-inflammatory	Spectrophotometry/Anti-COX-2/PGF_2_*_α_* inhibition	49.1 µM (IC_50_)	Indomethacin 18.3 µM (IC_50_) NS-398 1.0 µM (IC_50_)	[82]
Aladothalen (**160**)	Antimicrobial	Agar dilution method/*B. cereus*	50.0 µM (MIC)	Ciprofloxacin ˂ 0.4 µM (MIC)	[89]
		Agar dilution method/*M. phlei*	25.0 µM (MIC)	Ciprofloxacin ˂ 0.4 µM (MIC)	[89]
		Agar dilution method/MRCNS	25.0 µM (MIC)	Ciprofloxacin 25.0 µM (MIC)	[89]
Cladonaphchrom A (**169**)	Antimicrobial	Microplate assay/*S. albus*	1.25 µg/mL (MIC)	Ciprofloxacin 0.6 µg/mL (MIC)	[90]
		Microplate assay/*E. coli*	2.5 µg/mL (MIC)	Ciprofloxacin 0.3 µg/mL (MIC)	[90]
		Microplate assay/*B. subtilis*	10.0 µg/mL (MIC)	Ciprofloxacin 0.6 µg/mL (MIC)	[90]
		Microplate assay/*M. tetragenus*	5.0 µg/mL (MIC)	Ciprofloxacin 0.3µg/mL (MIC)	[90]
		Microplate assay/*M. luteus*	10.0 µg/mL (MIC)	Ciprofloxacin 0.3 µg/mL (MIC)	[90]
		Microplate assay/*A. brassicicola*	50.0 µg/mL (MIC)	Prochloraz 12.5 µg/mL (MIC)	[90]
		Microplate assay/*P. parasitica* var. *nicotianae*	50.0 µg/mL (MIC)	Prochloraz 50.0 µg/mL (MIC)	[90]
		Microplate assay/*C. capsici*	25.0 µg/mL (MIC)	Prochloraz 12.5 µg/mL (MIC)	[90]
		Microplate assay/*B. oryzae*	100.0 µg/mL (MIC)	Prochloraz 50.0 µg/mL (MIC)	[90]
		Microplate assay/*D. medusaea*	50.0 µg/mL (MIC)	Prochloraz 50.0 µg/mL (MIC)	[90]
		Microplate assay/*C. paradoxa*	50.0 µg/mL (MIC)	Prochloraz 25.0 µg/mL (MIC)	[90]
Cladonaphchrom B (**170**)	Antimicrobial	Microplate assay/*S. albus*	2.5 µg/mL (MIC)	Ciprofloxacin 0.6 µg/mL (MIC)	[90]
		Microplate assay/*E. coli*	2.5 µg/mL (MIC)	Ciprofloxacin 0.3 µg/mL (MIC)	[90]
		Microplate assay/*B. subtilis*	5.0 µg/mL (MIC)	Ciprofloxacin 0.6 µg/mL (MIC)	[90]
		Microplate assay/*M. tetragenus*	5.0 µg/mL (MIC)	Ciprofloxacin 0.3µg/mL (MIC)	[90]
		Microplate assay/*M. luteus*	10.0 µg/mL (MIC)	Ciprofloxacin 0.3 µg/mL (MIC)	[90]
		Microplate assay/*A. brassicicola*	25.0 µg/mL (MIC)	Prochloraz 12.5 µg/mL (MIC)	[90]
		Microplate assay/*P. parasitica* var. *nicotianae*	50.0 µg/mL (MIC)	Prochloraz 50.0 µg/mL (MIC)	[90]
		Microplate assay/*C. capsici*	25.0 µg/mL (MIC)	Prochloraz 12.5 µg/mL (MIC)	[90]
		Microplate assay/*D. medusaea*	100.0 µg/mL (MIC)	Prochloraz 50.0 µg/mL (MIC)	[90]
		Microplate assay/*C. paradoxa*	50.0 µg/mL (MIC)	Prochloraz 25.0 µg/mL (MIC)	[90]
Malettinin A (**178**)	Antimicrobial	Microplate assay/*T. rubrum*	33.1 µM (IC_50_)	Clotrimazole 0.2 µM (IC_50_)	[93]
Malettinin B (**179**)		Microplate assay/*X. campestris*	28.3 µM ((IC_50_)	Chloramphenicol 2.1 µM (IC_50_)	[93]
		Microplate assay/*T. rubrum*	60.6 µM (IC_50_)	Clotrimazole 0.2 µM (IC_50_)	[93]
Malettinin C (**180**)	Antimicrobial	Microplate assay/*T. rubrum*	37.9 µM (IC_50_)	Clotrimazole 0.2 µM (IC_50_)	[93]
		Microplate assay/*X. campestris*	83.2 µM (IC_50_)	Chloramphenicol 2.1 µM (IC_50_)	[93]
Malettinin E (**181**)	Antimicrobial	Microplate assay/*T. rubrum*	28.7 µM (IC_50_)	Clotrimazole 0.2 µM (IC_50_)	[93]
		Microplate assay/*X. campestris*	30.7 µM (IC_50_)	Chloramphenicol 2.1 µM ((IC_50_)	[93]
Cladosporinone (**182**)		Broth dilution assay/*S*. *aureus*	64.0 μg/mL(MIC)	-	[94]
Viriditoxin (**183**)		Broth dilution assay/*S*. *aureus*	0.015 μg/mL (MIC) 0.023 μM (MIC)	-	[94]
Viriditoxin derivative 1 (**184**)		Broth dilution assay/*S*. *aureus*	2.0 μg/mL (MIC)	-	[94]
Viriditoxin derivative 2 (**185**)		Broth dilution assay/*S*. *aureus*	16.0 μg/mL (MIC)	-	[94]
(2S,4S)-4-Methoxy-2-methylchroman-5-ol (**201**)	Antioxidant	DPPH assay	5.66 µM (IC_50_)	Ascorbic acid 3.29 µM (IC_50_)	[95]
(2S,4S)-2-Methylchroman-4,5-diol (**203**)	Antioxidant	DPPH assay	6.67 µM (IC_50_)	Ascorbic acid 3.29 µM (IC_50_)	[95]
Citrinin H1 (**206**)	Insecticidal	Measuring the corrected mortality (CM)	100.0 μg/mL (IC_50_)	Azadirachtin 25.0 μg/mL (IC_50_)	[71]
	Antimicrobial	Microplate assay/*S. aureus*	6.25 μg/mL (MIC)	Ciprofloxacin 0.39 μg/mL (MIC)	[71]
		Microplate assay/*E. coli*	12.5 μg/mL (MIC)	Ciprofloxacin 0.19 μg/mL (MIC)	[71]
		Microplate assay/*B. cereus*	12.5 μg/mL (MIC)	Ciprofloxacin 0.19 μg/mL (MIC)	[71]
Vermistatin (**214**)	Insecticidal	CM/Helicoverpa armigera Hubner larvae	150.0 μg/mL (IC_50_)	Azadirachtin 25.0 μg/mL (IC_50_)	[71]
	Antimicrobial	Microplate assay/*S. aureus*	25.0 μg/mL (MIC)	Ciprofloxacin 0.39 μg/mL (MIC)	[71]
		Microplate assay/*B. cereus*	25.0 μg/mL (MIC)	Ciprofloxacin 0.39 μg/mL (MIC)	[71]
Cladoscyclitol B (**219**)	α-Glucosidase inhibitory	Colorimetric assay	2.95 µM (IC_50_)	Acarbose 2.35 µM (IC_50_)	[97]
3-Phenyl-2-propenoic acid (**232**)	Insecticidal	Inhibitinon 50%/*B. amphitrite*	84.28 μg/mL (EC_50_)	FSW with DMSO	[60]
Cladosporinone (**182**)		Broth dilution assay/*S*. *aureus*	64.0 μg/mL(MIC)	-	[94]
		Lethality 50%/*B. amphitrite*	>200 μg/mL (LC_50_)	FSW with DMSO	[60]
		Inhibitinon 50%/*B. neritina*	11.15 μg/mL (EC_50_)	FSW with DMSO	[60]
		Lethality 50%/*B. neritina*	>200 μg/mL (LC_50_)	FSW with DMSO	[60]
	Antimicrobial	Serial dilution/*L. hongkongensis*	80 μg/mL (MIC)	Streptomycin 250 μg/mL (MIC) Penicillin 0.25 μg/mL (MIC)	[60]
3-(2,3-Dihydroxy phenoxy) butanoic acid (**233**)	Antioxidant	DPPH assay	0.24 µM (IC_50_)	Ascorbic acid 3.29 µM (IC_50_)	[95]
Methyl (3S)-3-(2,3-dihydroxy phenyloxy)butanoate (**235**)	Antioxidant	DPPH assay	2.65 µM (IC_50_)	Ascorbic acid 3.29 µM (IC_50_)	[95]
2-Phenylethanol (**238**)	Insecticidal	Inhibitinon 50%/*B. amphitrite*	53.65 μg/mL (EC_50_)	FSW with DMSO	[60]
		Lethality 50%/*B. amphitrite*	>200 μg/mL (LC_50_)	FSW with DMSO	[60]
		Inhibitinon 50%/*B. neritina*	102.23 μg/mL (EC_50_)	FSW with DMSO	[60]
		Lethality 50%/*B. neritina*	>200 μg/mL (LC_50_)	FSW with DMSO	[60]
4-*O*-*α*-*D*-Ribofuranose-2-pentyl-3-phemethylol (**240**)	α-Glucosidase inhibitory	Colorimetric assay	2.05 µM (IC_50_)	Acarbose 2.35 µM (IC_50_)	[97]
Cladosporin D (247)	Antioxidant	DPPH assay	16.4 µM (IC_50_)	Ascorbic acid 4.9 µM (IC_50_)	[36]
(2S)-7,4′-dihydroxy-5-methoxy-8-(γ,γ-dimethylallyl)-flavanone (**248**)	Protein tyrosine phosphatase 1B inhibitory	PTP1B/Spectrophotometry	11.0 µM (IC_50_)	Oleanolic acid 0.9 µM (IC_50_)	[68]
		TCPTP/Spectrophotometry	27.0 µM (IC_50_)	Oleanolic acid 0.8 µM (IC_50_)	[68]
Bis(2-ethylhexyl)phthalate (**249**)	Insecticidal	Inhibitinon 50%/*B. amphitrite*	9.18 μg/mL (EC_50_)	FSW with DMSO	[60]
		Lethality 50%/*B. amphitrite*	>200 μg/mL (LC_50_)	FSW with DMSO	[60]
		Inhibitinon 50%/*B. neritina*	77.85 μg/mL (EC_50_)	FSW with DMSO	[60]
		Lethality 50%/*B. neritina*	>200 μg/mL (LC_50_)	FSW with DMSO	[60]
1,1′-Dioxine-2,2′-dipropionic acid (**252**)	Insecticidal	Measuring the corrected mortality (CM)/Helicoverpa armigera Hubner larvae	150.0 μg/mL (IC_50_)	Azadirachtin 25.0 μg/mL (IC_50_)	[70,71]
	Antimicrobial	Microplate assay/*S. aureus*	25.0 μg/mL (MIC)	Ciprofloxacin 0.39 μg/mL (MIC)	[71]
		Microplate assay/*E. coli*	25.0 μg/mL (MIC)	Ciprofloxacin 0.19 μg/mL (MIC)	[71]
		Microplate assay/*B. cereus*	12.5 μg/mL (MIC)	Ciprofloxacin 0.39 μg/mL (MIC)	[71]
5α,8α-Epidioxy-ergosta-6,22E-dien-3β-ol (**256**)	Antiviral	Neuraminidase inhibition assay/RSV	0.11 µM (IC_50_)	Ribavirin 0.08 µM (IC_50_)	[100]
3β,5α-Dihydroxy-6β-methoxyergosta-7,22-diene (**262**)	Antiviral	Neuraminidase inhibition assay/RSV	0.11 µM (IC_50_)	Ribavirin 0.08 µM (IC_50_)	[100]
5α,8α-Epidioxy-ergosta-6,9,22E-triene-3β-ol (**258**)	Antiviral	Neuraminidase inhibition assay/RSV	0.17 µM (IC_50_)	Ribavirin 0.08 µM (IC_50_)	[100]
3*α*-Hydroxy-pregna-7-ene-6,20-dione = Cladosporisteroid B (**268**)	Antiviral	Neuraminidase inhibition assay/RSV	0.12 µM (IC_50_)	Ribavirin 0.08 µM (IC_50_)	[100]
		CPE inhibition assay/ Influenza A H3N3 virus	16.2 µM (IC_50_)	Oseltamivir 34.0 nM (IC_50_)	[61]
(3E,8E,6S)-Undeca-3,8,10-trien-1,6-diol (**286**)	Cytotoxicity	SRB/H1975	10.0 µM (IC_50_)	Adriamycin 0.38 µM (IC_50_)	[95]

Pectinases are hydrolytic enzymes that are accountable for the hydrolysis of pectins. They are commonly found in fungi, bacteria, and plants. They have remarkable importance in the food industry such as vegetables and fruits processing, wine production, and olive oil extraction, as well as coffee, cocoa, and tea fermentation. They are utilized in the beverage industry to produce high yields due to improving clarification and pressing of concentrated fruit juices [103]. Bastos et al. purified pectinase enzymes PG and PME from *C. cladosporioides* using the Buescher and Furmanski procedure after 10-day incubation and precipitation with (NH_4_)_2_SO_4_ and benzoate buffer at pH 4.0 [49].

Agarases and carrageenases can decompose algal biomass, producing carrageenans and agars that are the major components of the red algae cell wall. Furthermore, agarases hydrolyze agar, resulting in oligosaccharides that are employed as food additives with beneficial influences on human health [104,105]. Additionally, carrageenases are used to obtain carrageenans that have varied industrial applications as emulsifying, thickening, and gelling agents in the preparation of food, as well as bioactivities such as anti-tumor, antiviral, antithrombotic, immunomodulatory, anticoagulant, and antioxidant [106]. *Cladosporium* sp. isolated from the Antarctic macroalgae *Ascoseira mirabilis* and *Georgiella confuens* produced agarase that may have industrial importance in the extraction of agar or its byproducts such as bioactive galactose and oligosaccharides exist in the algal biomass to be utilized as substrates of 3rd generation bioethanol [11].

Xylan, the main component of hemicelluloses in the plant cell walls, represents about one-third of all renewable organic carbon on earth. Xylanases hydrolyze xylan to oligosaccharides that are further degraded to xylose. The latter is utilized for xylitol and bioethanol production. Xylanases have remarkable biotechnological influence in developing eco-friendly technologies in the pulp and paper industry and in food and feed industries, and for generating chemicals and liquid fuels from lignocellulose [107,108,109]. The cold-active xylanases have notable applications in bioremediation and food and textile and industries [110]. *Cladosporium* sp. isolated from Antarctic marine sponge had high xylanase potential when grown on wheat bran and pure xylans at lower temperatures that is a feature of cold-active enzymes [48]. Therefore, cold-active xylanases preparations from *Cladosporium* sp. could be convenient for many biotechnological processes, utilizing moderate- to low-temperature processes, especially those in food industries [48]. Gil-Durán et al. purified and characterized XynA, a cold-active endo-xylanase from *Cladosporium* sp. derived from Antarctic sponge. XynA is highly active on xylans with high arabinose content. Moreover, it is the most thermolabile endo-xylanase reported from filamentous fungus. Therefore, it could be a good alternative in some biotechnological operations to avoid heating, thereby reducing the costs [45].

The three main lignin-hydrolyzing enzymes that have great potential for industrial applications are LiP (lignin peroxidase), MnP (manganese-dependent peroxidase), and Lac (laccase) [111]. LiP is a high oxidant heme protein that oxidizes non-phenolic and phenolic substrates. MnP is a H_2_O_2_-dependent glycoprotein that needs Mn^2+^ for oxidizing aromatic dyes and mono-aromatic phenols [112]. Laccase is multi-copper oxidase, which oxidizes aromatic amines and catalyzes the O_2_ reduction to H_2_O [111]. *C. cladosporioides* CBMAI 857 isolated from the Brazilian cnidarian *Palythoa variabilis* produced ligninolytic enzymes (LiP, MnP, and Lac) with particular response to the various conditions of salinity and carbon sources. It possessed high values of MnP and laccase activities under salinity (12.5% and 23% *w*/*v*, respectively), indicating the potential use of this fungus for industrial applications and bioremediation of high-salt contaminated sites [50].

RBBR (Remazol Brilliant Blue R) and polymeric dyes decolorization has been assigned as an effective screening method for the fungi ability to degrade recalcitrant pollutants, including aromatic compounds such as PAHs. It was demonstrated that marine-derived fungi are often more effective than terrestrial fungi in treating various colored effluents because they are better adapted to perform under extreme conditions such as high salinity [113]. *C. cladosporioides* CBMAI-857 associated with the coral *Palythoa caribaeorum* was tested for its RBBR decolorizing potential. It had efficient dye decolorization potential (93%) after 12 days in both liquid and solid media [114]. Further, *Cladosporium* sp. associated with the seagrass *Posidonia oceanica* produced tannases and ligninolytic enzymes at high salt concentrations. Its laccase and peroxidase activity was evident by the degradation of RBBR and Amaranth Red dyes [12,115].

Invertase is a β-fructo-furanosidase that catalyzes sucrose conversion into fructose and glucose, giving invert syrup. This invert syrup is utilized in the beverage and food industries as a humectant in non-crystallizing creams, candies, artificial honey, and jam preparation [116]. Molasses is a sugar solution that is obtained as a co-product of sugar production. Due to its high sucrose content and low cost, it is utilized as an invertase production substrate to produce industrially valuable substances [117]. However, it contains melanoidins, which are dark brown pigments. Its discharge in the soil prohibits seed germination and decreases manganese availability and soil alkalinity. Furthermore, it blocks photosynthesis and sunlight penetration in the aquatic system [118]. Therefore, its removal from molasses-based wastewater is potentially important for environmental safety. Taskin et al. reported that *C. herbarum* ER-25 possessed a high invertase potential and removed melanoidins from molasses through bio-adsorption and biodegradation mechanisms by Lac and MnP in the non-sterilized medium than in sterilized one at 5.5 pH and 20 °C. Therefore, this cold-adapted fungus can be used for molasses de-colorization [46].

Cellulose is a main component of the plant material that is abundantly utilized for the production of alternative liquid fuels such as bioethanol. *C. sphaerospermum* obtained from deteriorated seaweed *Ulva* through SSF (solid-state fermentation) produced cellulase that had saccharification potential of seaweed biomass using green seaweed *Ulva* *fasciata*. Therefore, this cellulase can be utilized for saccharification of cellulosic feedstock for bioethanol production from marine macro-algal feedstock [47].

Biocatalysis is an eco-friendly process for renewable raw materials and clean energy production and for the remediation of environmental contaminants [119]. Recently, the synthesis of industrial and chemically interesting complex molecules using biocatalysts, including enzymes and whole-cell systems is a grown-research field. Reductases have been utilized for various substrates reduction such as aldehydes, carboxylic acid derivatives, ketones, nitro compounds, and nitriles [119,120]. Furthermore, it has been reported that microorganisms’ whole cells are a potential source for new enzymes used in carbonylated compounds reduction [121]. Knoevenagel condensation is a very useful synthetic tool for functionalization, as well as for increasing the carbon chains that is applied for the synthesis of intermediates polymers and various bioactive organic compounds [122]. Birolli et al. reported that the bio-reduction of Knoevenagel adducts between cyano-acetamide and aromatic aldehydes was achieved in considerable yields with whole-cells of *Cladosporium* sp. CBMAI 1237 isolated from *Dragmacidon reticulatum*, revealing the existence of ene-reductases [43]. Additionally, *C. cladosporioides* CBMAI-857 isolated from the Brazilian cnidarian *Palythoa caribaeorum* catalyzed the asymmetric bio-reduction of 1-(4-methoxyphenyl)ethanone to 1-(4-methoxyphenyl)ethanol [123]. Moreover, the sponge-associated *C. cladosporioides* CBMAI-857 catalyzed the enantio-selective bio-reduction of different aromatic ketones at pH 7.0 and 32 °C [124].

## 3. Secondary Metabolites and Bioactivities of Marine-Associated *Cladosporium* Species

Marine-associated *Cladosporium* species are rich with diverse types of metabolites with varied structural features such as macrolides, fatty acids, pyrones, phenolics, alkaloids, diketopiperazines, terpenes, sterols, quinones, lactones, and tetramic acid derivatives. Their classification was carried out here according to the chemical nature. During our search, it was found that some of the reported metabolites had the same structures and molecular formulae with different nomenclature. On the other hand, some metabolites had the same names with different structures. Moreover, some metabolites did not have names, thus they are named here using the AUPAC system for nomenclature. Herein, the reported secondary metabolites from *Cladosporium* species, as well as their bioactivities have been discussed (Table 1 and Table 2).

### 3.1. Tetramic Acid Derivatives

Tetramic acids are five-membered heterocycles with a pyrrolidine-2,4-dione core that are formed by the fusion of polyketide units and amino acid [125]. The tertarmic acid moiety is commonly present as 3-acyl or 4-*O*-alkyl ether derivatives [126]. These structures can be characterized as simple heterocycles or more complex systems possibly containing long chains or fused polycyclic skeletons [127]. They are found in varied natural metabolites and isolated from various terrestrial and marine species, such as bacteria, sponges, and fungi [127,128]. They exhibited a wide range of bioactivities: cytotoxic, antimicrobial, antiulcer, and antiviral [125]. Note that 30 tetramic acid derivatives have been reported from marine-derived *Cladosporium* species, 28 (93.3%) of them are from *C. sphaerospermum*.

**Figure 1:**
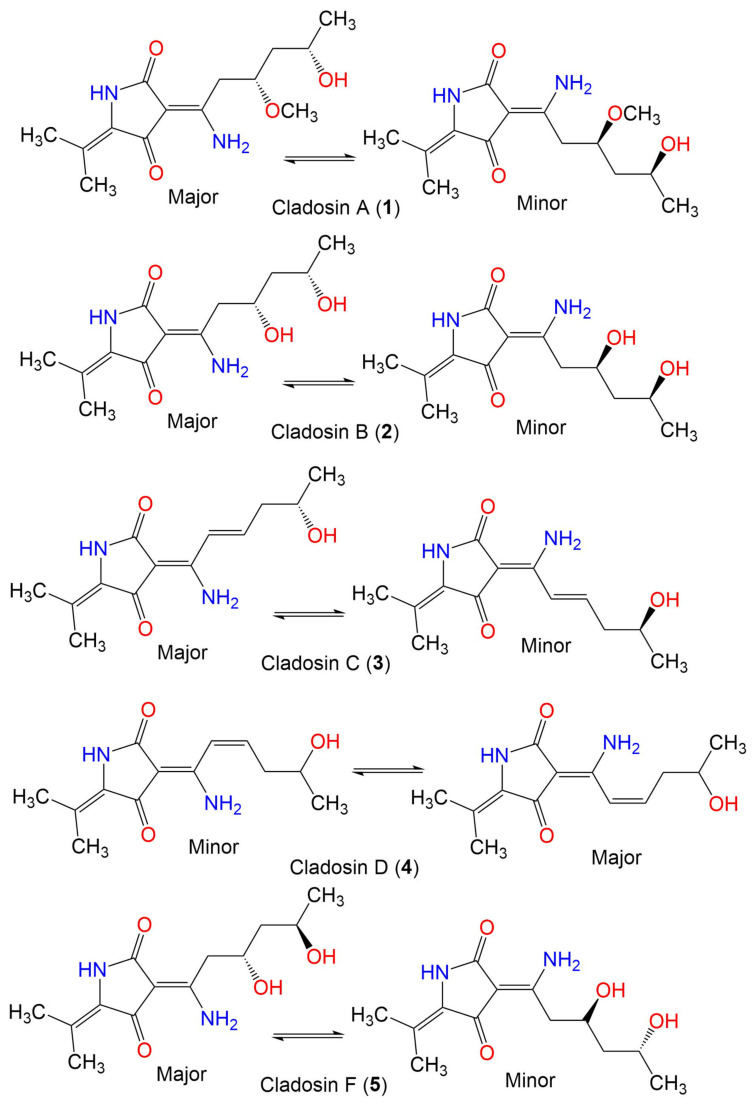
Tetramic acid derivatives **1**–**5**.

The tetramic acid derivatives, cladosins A, B, D, and E (**1**, **2**, **4**, and **5**) biosynthesized by *C. sphaerospermum* 2005-01-E3 obtained from deep-sea sludge had no activity towards influenza A H1N1 virus (Figure 1). While **3** exhibited anti-H1N1 activity (IC_50_ 276.0 μM) in comparison to ribavirin (IC_50_ 131.0 μM) [42]. Moreover, they showed no NF-κB inhibitory and no cytotoxic effect towards BGC-823, HL-60, HCT-8, A2780, A549, and Bel-7402 cell lines, as well as no activity towards *Mycobacterium tuberculosis* in the disk diffusion method [42]. Moreover, cladosins B (**2**), C (**3**), F (**5**), and L (**11**) separated from *C. sphaerospermum* SW67 associated with *Hydractinia echinat* hydroid polyp were assessed for protection towards cisplatin-caused cell damage in LLC-PK1 cells [53]. The co-treatment with compounds **2** and **5** alleviated the LLC-PK1 cells damage induced by cisplatin (Conc. 25 µM). Compound **2** (Conc. 100 µM) recovered cell viability with 90.68% that was more than NAC (*N*-acetylcysteine, 88.23%, Conc. 500 µM), whereas **5** (Conc. 50 and 100 μM) increased cell viability by 77.65 and 85.60%, respectively. Thus, **2** may be a candidate for treating cisplatin-produced unwanted effects and/or to prohibited nephrotoxicity induced by anticancer drugs. It was proposed that the existence of the C-8 hydroxy group may be essential for the reno-protective effect towards cisplatin-produced toxicity in LLC-PK1 cells [53].

**Figure 2:**
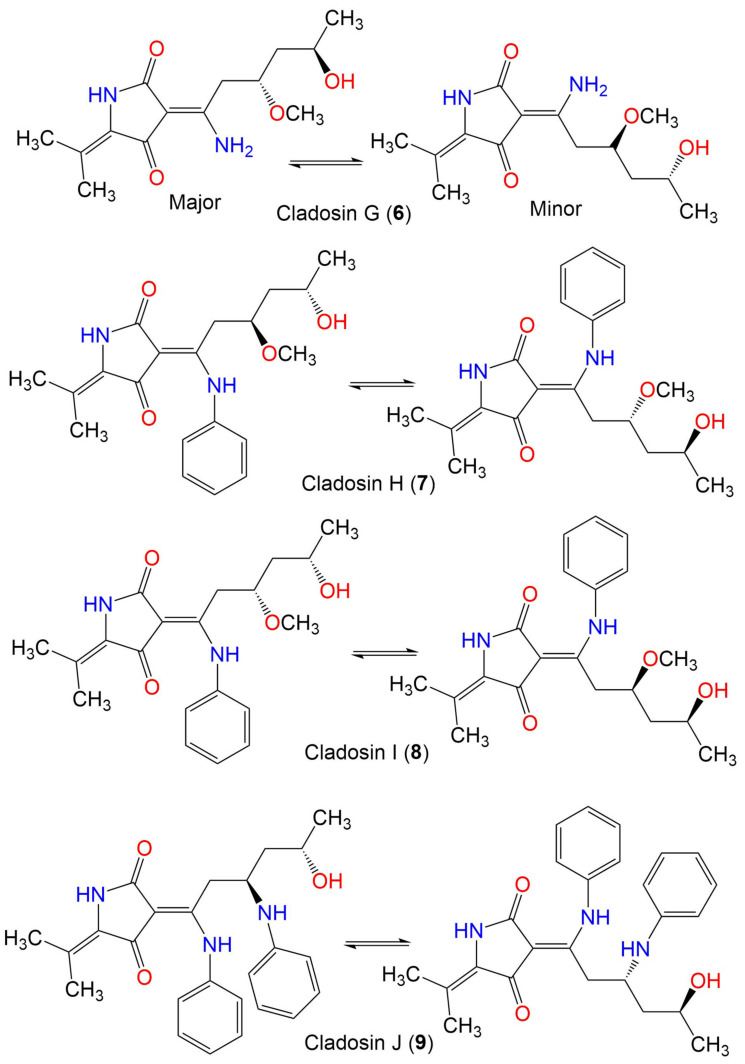
Tetramic acid derivatives **6**–**9**.

In 2015, by OSMAC (one strain many compounds) technique, Yu et al. separated compounds **5** and **6** from *C. sphaerospermum* 2005-01-E3 that did not have anti-influenza A H1N1, anticancer, and anti-tubercular, as well as no NF-κB inhibitory activities [54]. Note that a tetramic acid derivative named cladosin L with a different structure was separated in 2020 by Pan et al. from the plant-associated *C. sphaerospermum* WBS017 isolated from *Fritillaria unibracteata* var. *wabuensis* [14]. Cladosins H-K (**7**–**10**) and cladodionen (**13**) were isolated from sediment-derived *C*. *sphaerospermum* L3P3 and evaluated for cytotoxic capacity towards PC-3, MGC-803, SH-SY5Y, and HCT-116 cell lines using SRB method and against K562 and HL-60 using MTT method (Figure 2). Compounds **8**–**10** and **13** had a cytotoxic effect against HL-60 and K562 cell lines with IC_50_ ranging from 2.8 to 7.8 μM, while **7** (IC_50_ > 10 μM) was inactive. The results revealed that the C-8 absolute configuration and aniline moiety were essential for activity [55] (Table 2).

**Figure 3:**
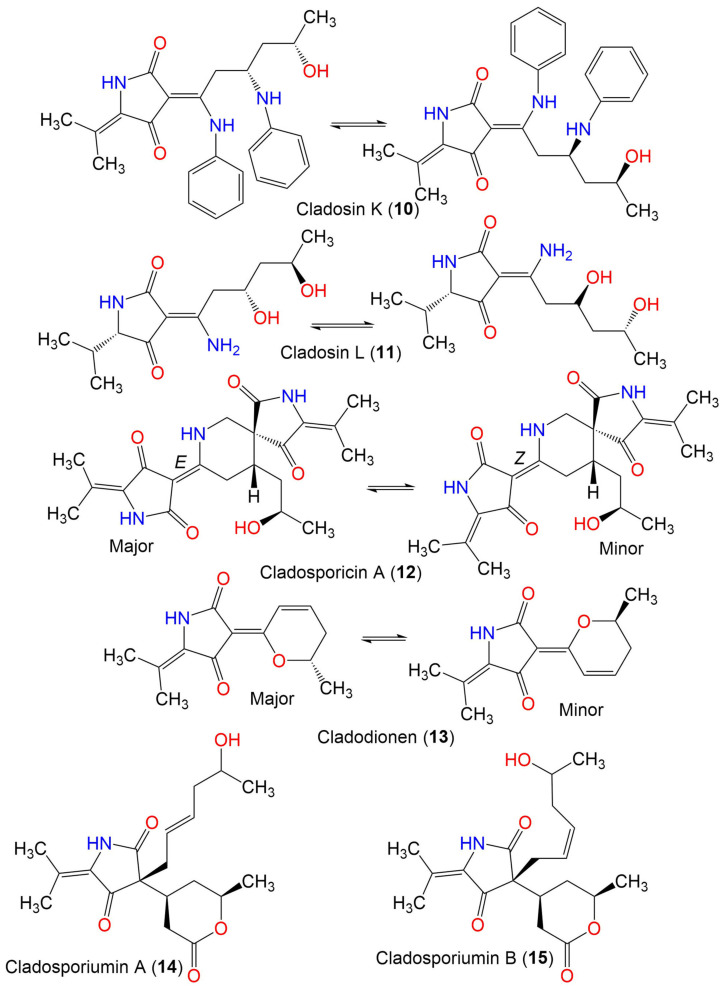
Tetramic acid derivatives **10**–**15**.

*C. sphaerospermum* SW67 associated with marine invertebrate *Hydractinia echinata* yielded three new spirocyclic tetramic acid-related metabolites **12**, **29**, and **30** (Figure 3). Compound **12** has a tetramic acid moiety conjugated with an unprecedented 2,7-diazaspiro[4.5]decane-1,4-dione core one, while **29** and **30** are tetramic acid stereoisomers with a C-3 quaternary center, bearing a six-membered lactone ring and a *trans*-hexylenic alcohol side chain. These metabolites had weak inhibitory effects versus HCC70, Bt549, MDA-MB-468, and MDA-MB-231 in the SRB bioassay (IC_50_ ranged from 70 to 85 μM), compared to etoposide (IC_50_ ranged from 1.76 to 2.27 μM) [38].

**Figure 4:**
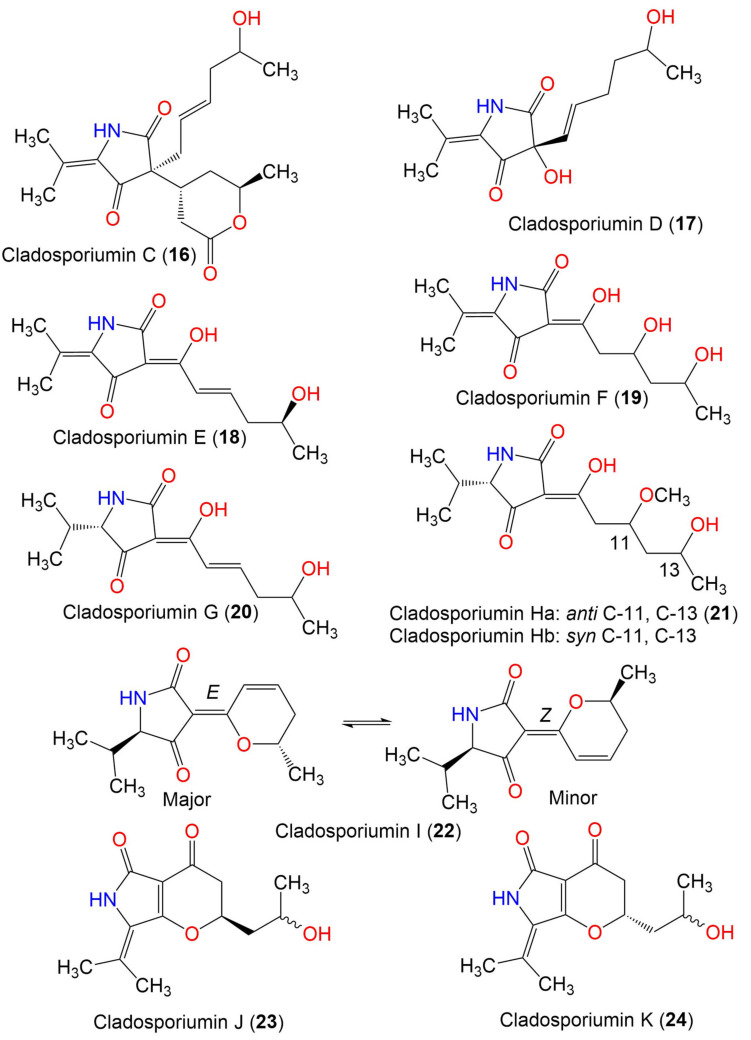
Tetramic acid derivatives **16**–**24**.

**Figure 5:**
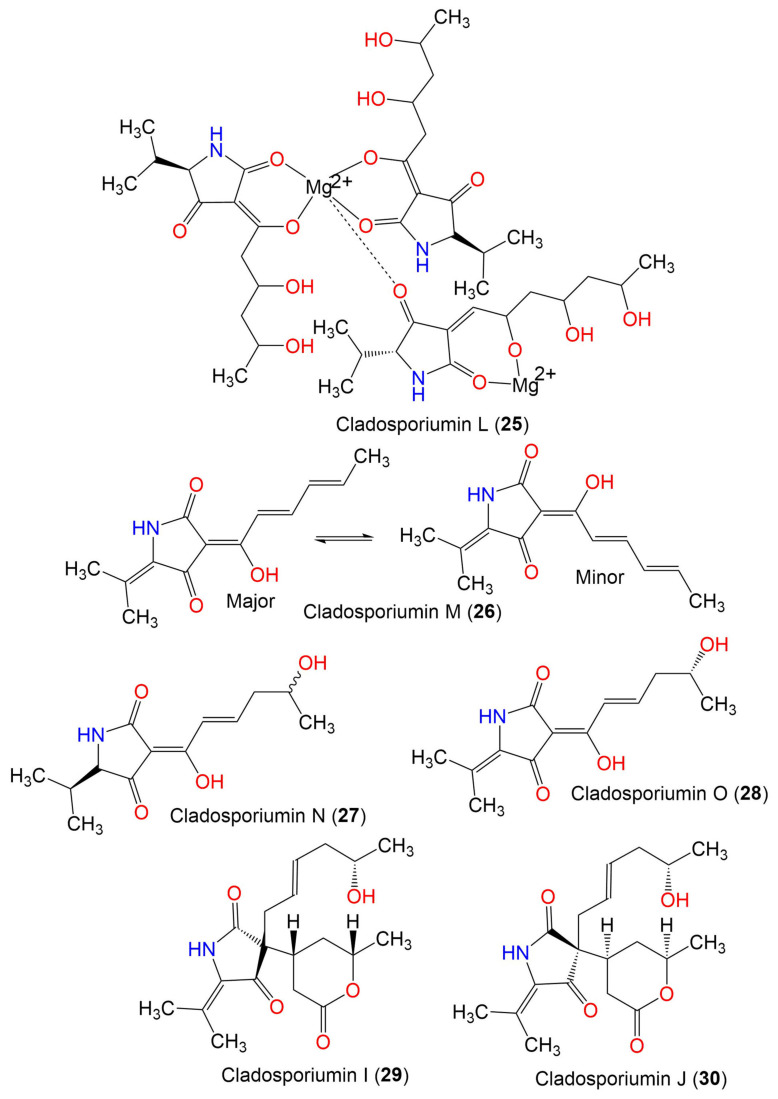
Tetramic acid derivatives **25**–**30**.

*C*. *sphaerospermum* EIODSF 008 isolated from the deep-sea sediment collected from the East Indian Ocean yielded tetramic acid derivatives **13** and **22**–**28** (Figure 4 and Figure 5). They were assessed for cytotoxicity towards HL-60, HepG2, and MCF-7. Only **13** had cytotoxicity (IC_50_ 28.6 µM) towards the HL-60 cell line [57]. Additionally, they showed no antibacterial potential towards *E. coli*, *M. luteus*, and *B subtilis* [57]. Additionally, **13** showed cytotoxic capacity towards HL-60, HeLa, HCT-116, and MCF-7 cell lines (IC_50_ ranged from 9.1 to 19.1 μM), compared to ADR (adriamycin) (IC_50_ ranged from 0.02 to 0.67 μM). However, it did not have antibacterial activities (conc. 100 μg/mL) against *B. subtilis*, *P. aeruginosa*, *C. perfringens*, *S. aureus*, *E. coli*, and *C. albicans* [56]. Compounds **14**–**21**, new tetramic acid derivatives, were purified from the sea-sediment derived *Cladosporium* sp. acetone extract by Huang et al. in 2018. Compounds **14**–**16** are unusual 3-acyltetramic acids, having at C-3 of the pyrrolidine-2,4-dione core, a six-membered lactone ring, and hexyl-enic alcohol chain. They showed no obvious AchEI activity in the modified Ellman’s enzyme assay [58]. Moreover, they displayed no anti-biofilm effect against *C. albicans* and *S. aureus* in the broth micro-dilution method and no cytotoxic effect towards HL60, HepG-2, and MCF-7 cell lines in the CCK8 assay [58].

### 3.2. Diketopiperazines

Diketopiperazines (DKPs) are cyclic dipeptides, consisting of two amino acids with or without extra structural modifications in the DKPs nucleus [108]. Their main skeleton comprises a six-membered piperazine nucleus produced from the double condensations among two amino acids [129,130]. The formation of peptide bonds in DKPs are catalyzed mainly by cyclodipeptide synthases (CDPSs) and non-ribosomal peptide synthetases (NRPSs) [131]. They possessed interesting bioactivities such as anti-Alzheimer, antimicrobial, antiviral, microtubule polymerization inhibitory, antitumor, anti-quorum-sensing, and haemosuppressor [129,130,132].

**Figure 6:**
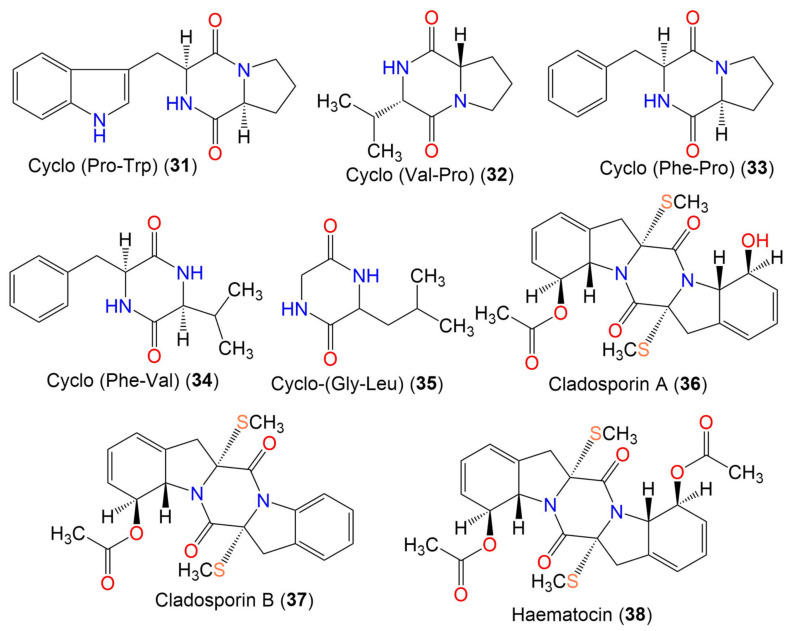
Diketopiperazine derivatives **31**–**38**.

Cyclo-(Val-Pro) (**32**) and cyclo-(Phe-Pro) (**33**) were separated from the EtOAc extract of *Cladosporium* sp. F14 isolated from seawater and investigated for their anti-larval activity at conc. 50 µg/mL towards *Bugula neritina* and *Balanus amphitrite* larvae in the settlement inhibition assays [60] (Figure 6). They inhibited *B*. *neritina* settlement (EC_50_ 70.43 and >200 µg/mL, respectively) and *B*. *amphitrite* settlement (EC_50_ 68.57 and 37.82 µg/mL, respectively). Furthermore, **32** and **33** obviously prohibited *L. hongkongensis* growth (IZDs 8 mm and MICs 200 and 200 µg/mL, respectively), compared to streptomycin (MIC 250 µg/mL). The MICs of **33** towards *Ruegeria* sp. and *M*. *luteus* were 200 and 100 µg/mL, respectively, compared to streptomycin (MIC 500 and 250 µg/mL, respectively) [60]. On the other hand, thio-diketopiperazine derivatives, cladosporins A (**36**) and B (**37**), and haematocin (**38**) purified from the sediment-derived *Cladosporium* sp. were moderately cytotoxic towards HepG2 cell line (IC_50_ 48, 21, and 42 μg/mL, respectively) [62].

### 3.3. Alkaloids

Fungal alkaloids are nitrogen-containing metabolites that are derived from amino acid metabolism and the mevalonate pathway [133]. Many studies reported the detection of various classeses of alkaloids from marine-derived fungi such as pyrrolidine, indole, pyrrolizidine, quinazoline, quinoline, and purine classes [134,135,136]. These metabolites have shown broad biological activities: cytotoxic, anti-inflammatory, antioxidant, antibacterial, antifungal, antiviral, protease inhibitory. Therefore, they could have a potential for the development of innovative therapies [134,135,136]. In the current work, 49 alkaloids, belonging to different classes have been reported. Among them, 27 alkaloids were reported from unidentified *Cladosporium* species.

**Figure 7:**
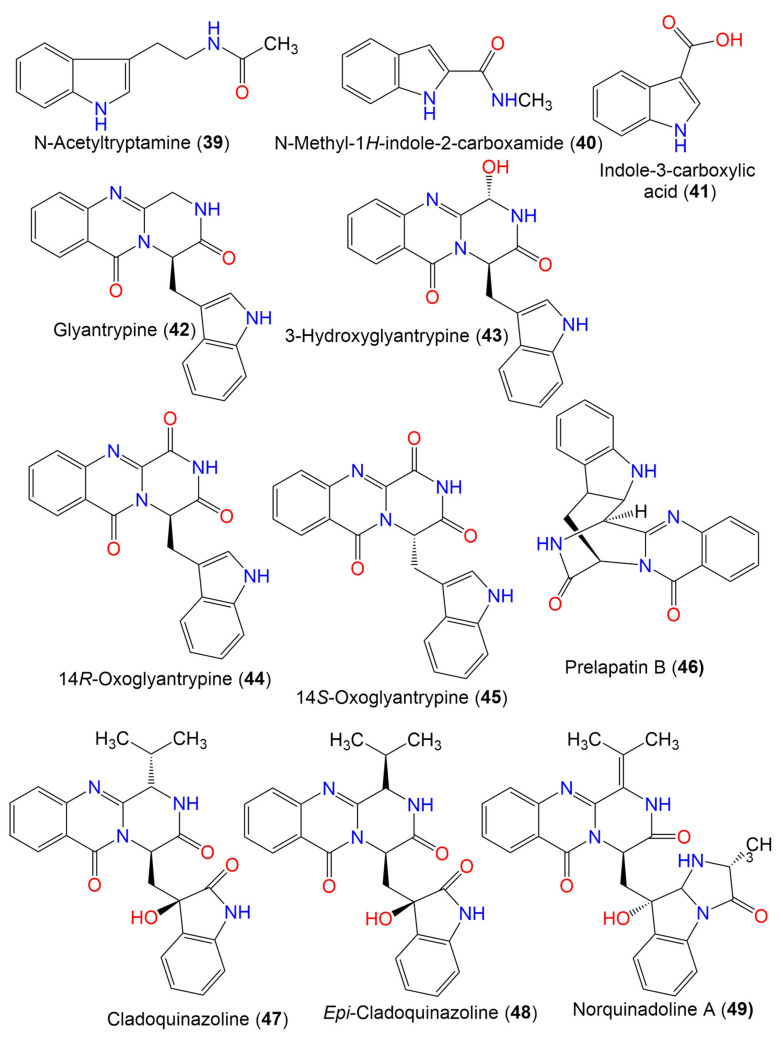
Alkaloids **39**–**49**.

**Figure 8:**
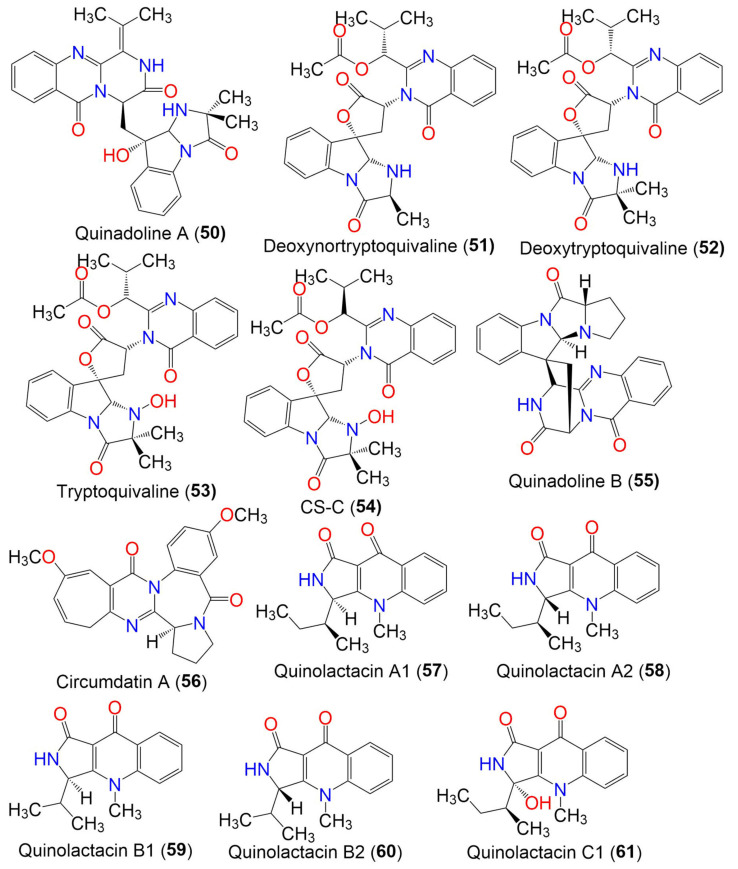
Alkaloids **50**–**61**.

**Figure 9:**
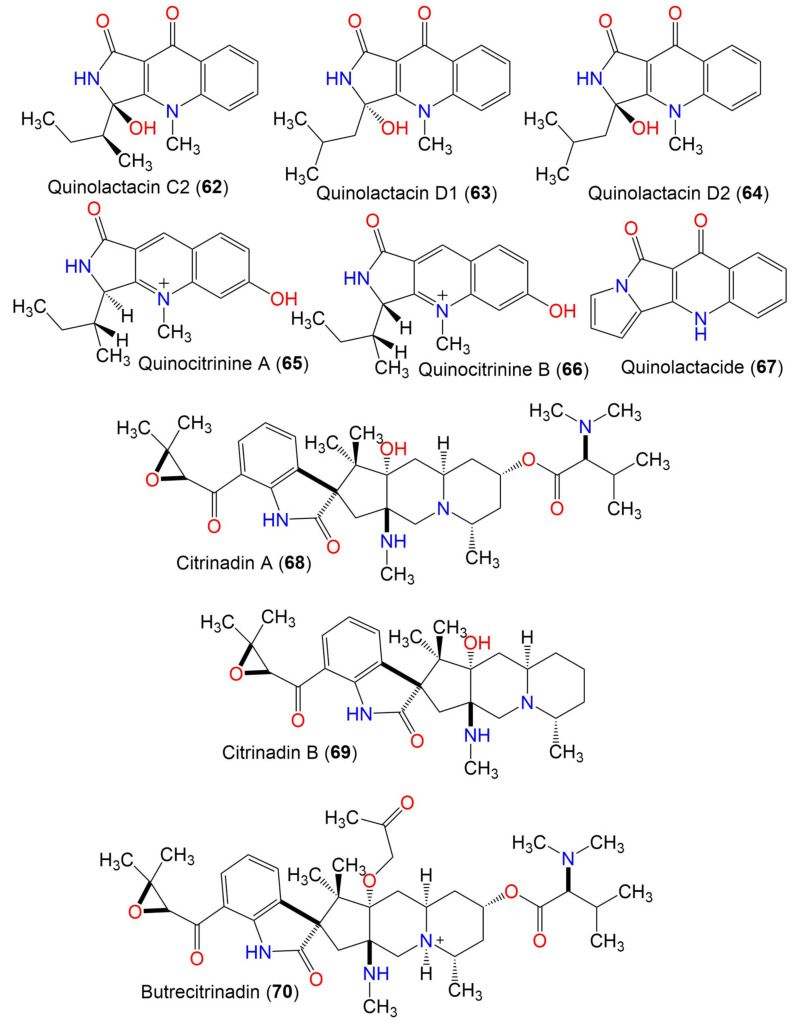
Alkaloids **62**–**70**.

The glyantrypine-type alkaloids, **42**–**55**, were separated from *Cladosporium* sp. PJX-41 isolated from mangrove and assessed for anti-H1N1 activity using CPE (cytopathic effect) inhibition assay (Figure 7 and Figure 8). Compounds **45**, **49**, **51**–**53**, and **55** displayed remarkable anti-H1N1 activities (IC_50_ values ranged from 82 to 89 μM), compared to ribavirin (IC_50_ 87 μM), while **42**–**44**, **46**–**48**, **50**, and **54** (IC_50_ 100–150 μM) had weak activity [64]. The mycelium extract of the marine-derived *Cladosporium* sp. associated with *Chondria crassicualis* red alga afforded **56** that exhibited antioxidant potential (ED_50_ 82.0 µM) more than oxybenzone (sunscreen agent, ED_50_ 350 µM) as evident by their UV-A protecting potential [65]. Furthermore, it had a moderate antibacterial effect towards multidrug-resistant and methicillin-resistant *S. aureus* and *S*. *aureus* with MICs 31.0, 62.5, and 62.5, µg/mL, respectively [65]. The quinolactacins and citrinadins alkaloids **58**, **68**, and **70** separated from C. oxysporum were assessed for anti-plasmodial potential towards chloroquine-sensitive *Plasmodium falciparum* 3D7 [66] (Figure 9). Only **58** (conc. 3.13 µg to 25.0 µg) had an anti-plasmodial effect (EC_50_ 24.8 µM), while **68** and **70** displayed no activity (EC_50_ > 25.0 µM), compared to artesunate (EC_50_ 0.074 μM) in the SYBR Green I assay. Further, **58** (conc. ranged from 6.25 µM to 50.0 µM for 24 h) was investigated for apoptotic effect on 3D7-plasmodia strain by measuring the parasite ΔΨm (mitochondrial membrane potential). It induced loss of ΔΨm, leading to the release of cytochrome C from mitochondria to the cytosol resulted in parasite apoptosis. Therefore, it may provide a scaffold to apoptotic death in the stages of *P. falciparum* development [66]. Moreover, **58**, **68**, and **70** had no anti-buruli ulcer activity against *Mycobacterium ulcerans* (IC_50_ ˃ 10 µM), compared to rifampicin (IC_50_ ˂ 1 µM) in the Resazurin microtiter assay [66].

**Figure 10:**
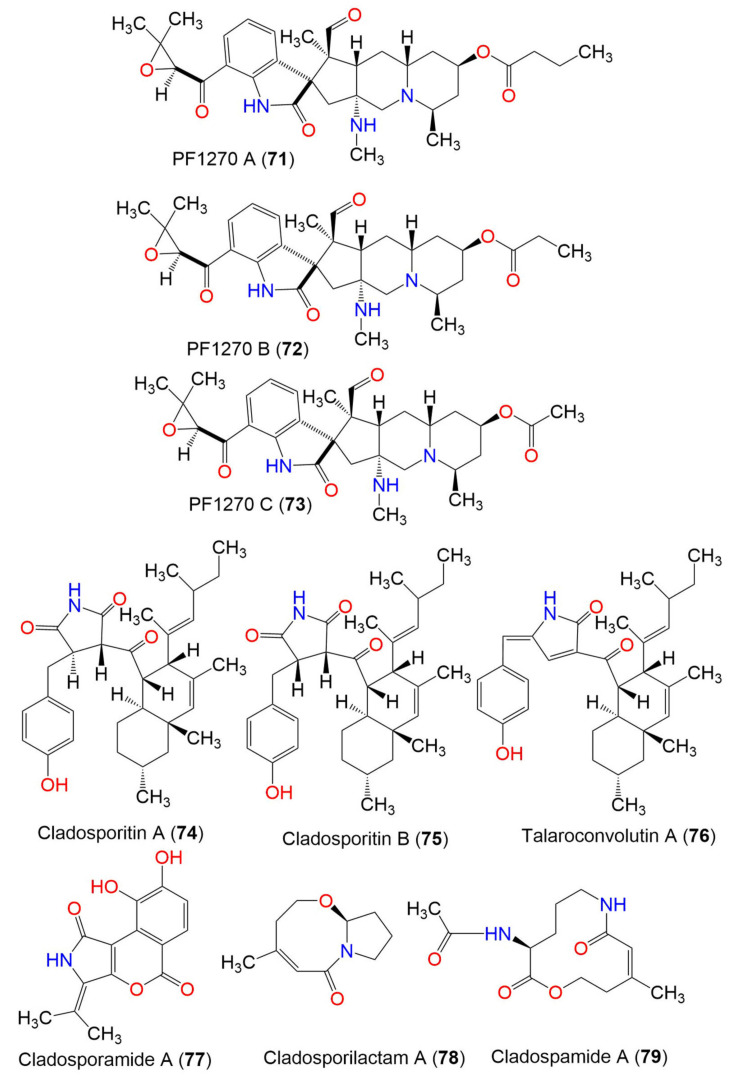
Alkaloids **71**–**79**.

They had significant activity towards HepG-2 and MCF-7 (IC_50_ ranging from 78.57 to 96.54 µM and from 51.32 to 94.49 µM, respectively), compared to curcumin (IC_50_ 61.38 and 20.68 µM, respectively). However, they showed moderate activity versus LNCap and LNCap (IC_50_ ranging from 32.94 to 45.71 µM and from 54.47 to 60.31 µM, respectively), in comparison to curcumin (IC_50_ 6.15 and 13.78 µM, respectively) in the MTT assay [66]. *Cladosporium* sp. HNWSW-1 associated with the mangrove plant *Ceriops tagal* biosynthesized compounds **74**–**76** that were assessed for their cytotoxic and α-glycosidase inhibitory effects (Figure 10). Compound **75** had cytotoxicity versus SGC-7901, K562, and BEL-7042 cell lines (IC_50_ 41.7, 25.6, and 29.4 μM, respectively), whereas **76** revealed cytotoxic potential towards BEL-7042 and Hela cell lines (IC_50_ 26.7 and 14.9 µM µM, respectively) in the MTT assay.

Additionally, **76** exhibited α-glucosidase inhibitory activity (IC_50_ 78.2 µM), compared to acarbose (IC_50_ 275.7 µM) in the glucose oxidase method [67]. Cladosporamide A (**77**) separated from *Cladosporium* sp. TPU1507 derived from marine sponge was assessed for its inhibitory effect towards PTP1B (protein tyrosine phosphatase) and TCPTP (T-cell PTP), using an enzyme-based assay [68]. It had mostly equivalent inhibition towards TCPTP and PTP1B (IC_50_ 48 and 54 μM, respectively), in comparison to oleanolic acid (IC_50_ 0.9 μM) [68]. Cao et al. purified a new 7-oxabicyclic[6.3.0]lactam, **78**, from a gorgonian-derived *Cladosporium* sp. collected from the South China Sea. It (IC_50_ 0.76–3.11 μM) exhibited significant cytotoxicity towards HeLa, P388, HT-29, and A549 cell lines [69]. On the other hand, it had weak antibacterial activity (MIC ˃ 25.0 μM) in broth dilution assay towards *B. cereus*, *T. halophilus*, *S. epidermidis*, *S. aureus*, *E. coli*, *P. putida*, *N. brasiliensis*, and *V. parahaemolyticus* [69]. *Cladosporium* sp. SCNU-F0001 isolated from a mangrove plant yielded a novel lactam macrolide named cladospamide A (**79**) that was evaluated for cytotoxic effect (conc. 50 μM) versus MDA-MB-435, A549, HCT116, HepG2, and BT549 in the MTT method and for antimicrobial potential (conc. 100 μg/mL) towards *S. aureus*, *B. subtilis*, *E. coli, Salmonella* ATCC 14028, and *P. aeruginosa*. Unfortunately, it exhibited no noticeable activity [70].

**Figure 11:**
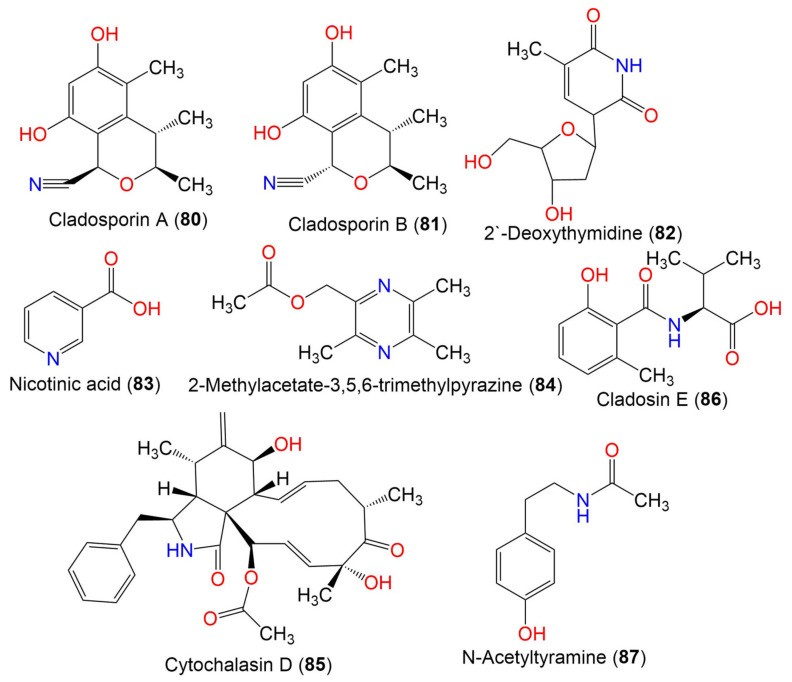
Alkaloids **80**–**87**.

The new cyano-containing alkaloids, cladosporins A (**80**) and B (**81**) purified from *Cladosporium* sp. SCSIO z015 broth did not have an obvious anti-biofilm activity towards *S. aureus*, *E. coli*, and *B. subtilis* [36] (Figure 11).

In the DPPH assay, they also had no activity (IC_50_ > 100 µM), compared to ascorbic acid (IC_50_ 4.9 µM). Besides, they showed moderate toxicity towards brine shrine naupalii (LC_50_s 72.0 and 81.7 µM, respectively), compared with toosendanin (LC_50_ 21.2 µM) in the brine shrimp lethality assay [36]. In 2019, Bai et al. purified **84** and **85** from *Cladosporium* sp. JS1-2 isolated from the mangrove *Ceriops tagal* collected in the South China Sea. Compound **84** moderately prohibited the growth of Helicoverpa armigera Hubner newly hatched larvae (IC_50_ 100 μg/mL), compared to azadirachtin (IC_50_ 25 μg/mL). Further, they showed moderate antibacterial potential versus *S. aureus* with MICs 12.5 and 25.0 μg/mL, respectively, compared with ciprofloxacin (MIC 0.39 μg/mL) [71].

### 3.4. Macrolides

**Figure 12:**
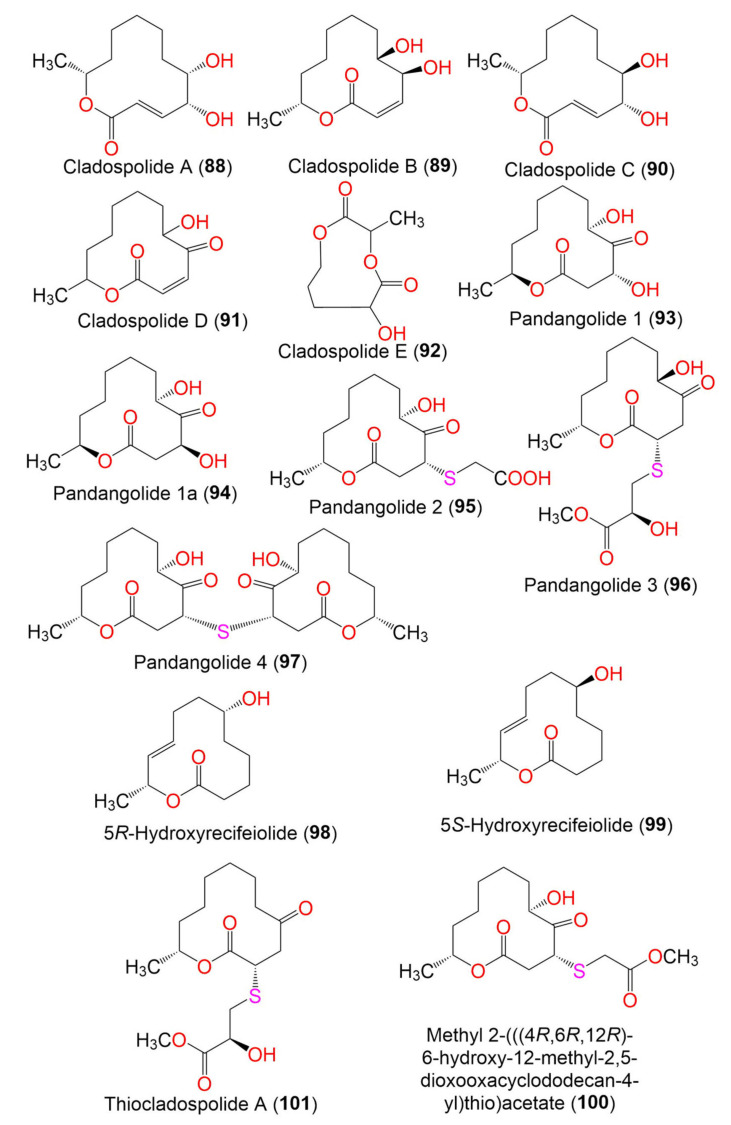
Macrolides **88**–**101**.

The term “macrolides” was first used to describe the natural antibiotics that have 12–16-membered macrocyclic lactone ring, functionalized by double bonds, and carrying different aminosaccharide and saccharide components [137]. Among these macrolides are 14-membered lactones (erythromycin and clarithromycin), 15-membered macrolides (azithromycin and spiramycin), and the 16-membered (avermectin B1a) that are clinically used macrolide antibiotics [138]. Members of this group possess a wide range of bioactivities such as antibacterial, anti-inflammatory, antiviral, antimalarial, antimitotic, and anticancer activity. They have been reported from various marine organisms [138,139]. The new 12-membered macrolide, cladospolide D (**91**), together with **88** and **89** were separated from *Cladosporium* sp. FT-0012 was obtained from Pohnpei Island, Federated State of Micronesia, and assessed for antimicrobial activity using paper disks at conc. 10 µg/disk (Figure 12). Compound **91** exhibited activity versus *M. racemosus* KF223, *B. subtilis* KB27, and *P. oryzae* KB110 (IZDs 11.5, 16.0, and 14.0 mm, respectively), while **88** was active (IZD 14.0 mm and IC_50_ 17.0 µg/mL) towards *X. campestris* pv. *oryzae*. Moreover, **91** prohibited *P*. *oryzae* and *M*. *racemosus* growth (IC_50_s 29.0 and 0.15 µg/mL, respectively) [72].

**Figure 13:**
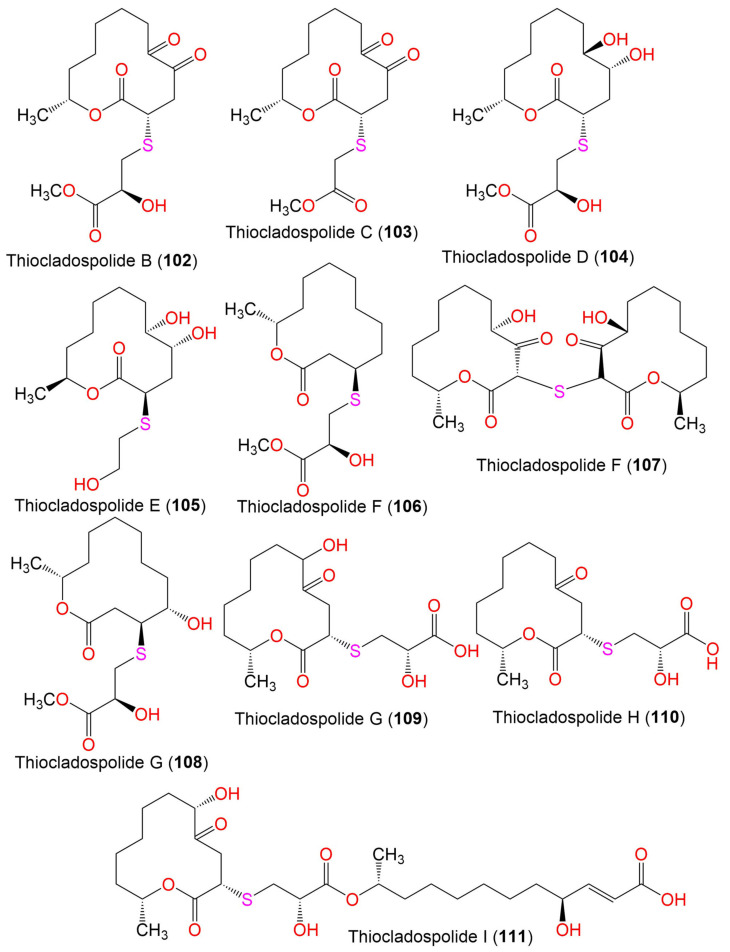
Macrolides **102**–**111**.

*Cladosporium* sp. F14 isolated from seawater yielded a nine-membered macrolide, **92** that had weak antibacterial potential towards *M. smegmatis*, *E. coli*, *B. thuringiensis*, *S. aureus*, and *B. subtilis* and weak cytotoxic potential toward A435, HeLa, K562, and A549 in the MTT method [76]. *C. herbarum* isolated from *Callyspongia aerizusa* sponge yielded cladospolide B (**89**) and pandangolides 2–4 (**95**–**97**) that showed no antimicrobial potential versus *S. aureus* ATCC 25923, *B. subtilis* 168, *E. coli* ATCC 25922, and *C. albicans* in the agar plate diffusion assay [74]. Moreover, the EtOAc extract of *Cladosporium* sp. IFB3lp-2 isolated from the mangrove forest of Hainan province of China yielded **88**, **89**, **93**–**96**, **100**, and **116** that had no significant activity against HCT-116, Coxsachievirus A16, A549, MD-MBA-231, HepG2, human enterovirus 71, A375, and SW1116 cell lines (conc. 20 µM) in the MTT assay [73] (Figure 13).

**Figure 14:**
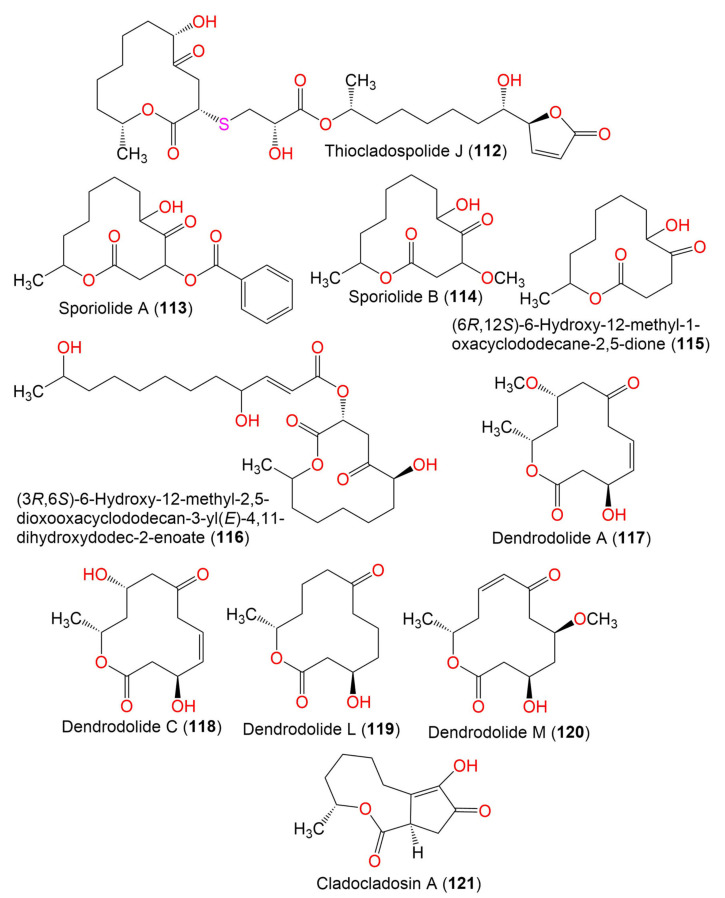
Macrolides **112**–**121**.

Additionally, *Cladosporium* sp. SCNU-F0001 isolated from a mangrove plant biosynthesized a new macrolide thiocladospolide E (**105**), along with **89** that were evaluated for cytotoxic effect (conc. 50 μM) versus MDA-MB-435, A549, HCT116, HepG2, and BT549 in the MTT method and for antimicrobial potential (conc. 100 μg/mL) towards *S. aureus*, *B. subtilis*, *E. coli*, *Salmonella* ATCC 14028, and *P. aeruginosa*. Unfortunately, none of them exhibited noticeable activity [70]. Cao et al. purified 12-membered macrolides **89** and **117**–**120** from a gorgonian-derived *Cladosporium* sp. collected from the South China Sea. They showed no cytotoxicity towards HeLa, P388, HT-29, and A549 cell lines [69]. Furthermore, they were evaluated for antibacterial activity in broth dilution assay towards *B. cereus*, *T. halophilus*, *S. epidermidis*, *S. aureus*, *E. coli*, *P. putida*, *N. brasiliensis*, and *V. parahaemolyticus*. Compounds **117**–**119** exhibited antibacterial potential against all tested bacteria (MIC values ranging from 3.13 to 25.0 μM), however **89** and **120** had weak activity (MIC ˃ 25.0 μM) [69]. The metabolites **93** and **115** separated from *Cladosporium* sp. F14 at conc. 50 µg/mL had no anti-larval activity towards both *B. neritina* and *B. amphitrite* larvae in the settlement inhibition assays [60]. In 2019, Zhang et al. separated the new polyketides **98** and **99** and a known analog **93** from the rice culture EtOAc extract of *C. cladosporioides* associated with *Bruguiera gymnorrhiza*. Their configuration was established using ECD, modified Mosher’s, and X-ray diffraction methods, as well as optical rotations to be 5R, 11R for **98**; 11R for **99**; and 3R, 5*S*, 11*S* for **93**. They had weak AChEI activity (IC_50_ > 50 µM), in comparison to tacrine in the modified Ellman’s method [40]. *C*. *cladosporioides* MA-299 obtained from the mangrove plant *B. gymnorrhiza* yielded 12-membered thio-macrolides **96** and **101**–**104** that were assessed for antimicrobial potential against *E. tarda* QDIO-2 and *E. ictarda* QDIO-9 (aquatic pathogens) and *C. glecosporioides* QDAU-2, *B. sorokiniana* QDAU-5, *P. piricola Nose* QDAU-15, and *F*. *oxysporum* f. sp. *cucumerinum* QDAU-8 (plant pathogenic fungi) in the microtiter plates assay. All metabolites revealed activity against *C. glecosporioides* (MIC 1 or 2 μg/mL), compared to amphotericin B (MIC 0.5 μg/mL). Moreover, **101** and **104** showed noticeable activity (MIC 1.0 μg/mL) towards with *E. tarda* and *E*. *ictarda*, respectively, compared to chloramphenicol (MIC 0.5 μg/mL), while **102** and **104** exerted obvious effectiveness (MIC 1.0 μg/mL) versus *F. oxysporum* f. sp. *cucumerinum*, compared to amphotericin B (MIC 0.5 μg/mL). The data revealed that sulfur substituent may influence the macrolides’ bioactivities [39]. The newly reported 12-membered macrolides having thioethers **107**–**112** and the related formerly reported **93** and **101** isolated from mangrove-derived *C. oxysporum* HDN13-314 had no cytotoxic activity versus HCT-116, BEL-7402, HL-60, A549, L-02, HeLa, K562, MGC-803, MCF-7, PC-3, SH-SY5Y, and MDA-MB-231 (IC_50_ > 50 μM) [78] (Figure 14).

Additionally, they exerted antibacterial activities versus the aquatic pathogens *E. ictarda* and *E. tarda* (MICs ranging from 4 to 32 μg/mL), whereas **108** had the best effect (MIC 4 μg/mL) versus *E. tarda* [78]. In 2020, new thiomacrolides thiocladospolides F (**106**) and G (**108**) and cladocladosin A (**121**), a macrolide with bicyclo 5/9-ring, were purified from *C*. *cladosporioides* MA-299 by Zhang et al. and assessed for antimicrobial effect versus various plant, human, and aquatic pathogenic microbes in the microtiter plates assay. All metabolites revealed activity (MIC ranging from 1.0 to 4.0 μg/mL) towards *V. anguillarum* and *E. tarda* (aquatic pathogenic bacteria) [79].

Moreover, **108** and **121** exerted activity (MICs 4.0 μg/mL) towards *H. maydis* (plant-pathogenic fungus) and *P. aeruginosa* (aquatic-pathogenic bacterium), respectively [79]. The new 12-membered macrolides, **113** and **114**, purified from *Cladosporium* sp. L037 isolated from the Okinawan marine brown alga *Actinotrichia fragilis* exhibited cytotoxic influence (IC_50_ 0.13 and 0.81 µg/mL, respectively) towards L1210 murine lymphoma cells in the MTT assay [80]. Moreover, **113** had antifungal potential against *C. albicans*, *C. neoformans*, *A. niger*, and *N. crassa* (MICs 8.4–16.7 µg/mL), whereas **114** exhibited antibacterial activity only towards *M. luteus* and inactive against the other microorganisms [80].

### 3.5. Butanolides and Butenolides

Butanolides and butenolides are five-membered γ-lactones which may also be regarded as furan derivatives. They are an important class of structural motifs often encountered in various natural metabolites and synthetic targets [140]. They have an impressive range of bioactivities including antibiotic, antitumor, and anticancer that are intimately connected to their relative and absolute configurations [141].

**Figure 15:**
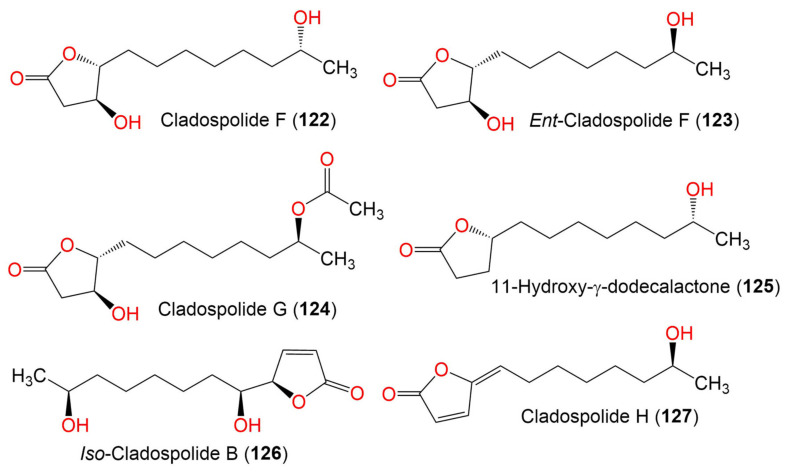
Butenolides and butanolides **122**–**127**.

The newly separated C12-macrolide, cladospolide F (**122**), purified from a soft coral-associated fungus *Cladosporium* sp. TZP-29, together with the formerly isolated derivative **126** showed no cytotoxic effect towards A-549, SMMC-7721, and HeLa cells in the SRB method [41]. Wuringege et al. reported that the butenolide, **126** isolated from *Cladosporium* sp. IFB3lp-2 exhibited no significant activity against HCT-116, Coxsachievirus A16, A549, MD-MBA-231, HepG2, human enterovirus 71, A375, and SW1116 cell lines (Conc. 20 µM) in the MTT assay [73]. Moreover, it showed no cytotoxicity towards various cancer cell lines: HeLa, P388, HT-29, HCT-116, BEL-7402, HL-60, A549, L-02, HeLa, K562, MGC-803, MCF-7, PC-3, SH-SY5Y, MDA-MB-231, and A549 [69,78]. On the other hand, it had antibacterial activity in broth dilution assay towards *B. cereus*, *T. halophilus*, *S. epidermidis*, *S. aureus*, *E. coli*, *P. putida*, *N. brasiliensis*, and *V. parahaemolyticus* (MIC values ranging from 6.25 to 25.0 μM) [46]. Qi et al. stated that **126** displayed no anti-larval activity towards both *B. neritina* and *B. amphitrite* larvae in the settlement inhibition assays [60]. Additionally, it exerted antimicrobial activity versus *E. ictarda* and *Cytospora mandshurica Miura* (MIC 8 μg/mL) [78]. The new metabolites **123**, **124**, and **127** and the known analog **126** separated from *C. cladosporioides* were assessed for AChEI activity using modified Ellman’s method (Figure 15). Only **123** exhibited potent AChEI activity with the IC_50_ value of 40.26 µM, in comparison to tacrine, while other metabolites possessed weak activity (IC_50_ > 50 µM) [40].

### 3.6. Seco-Acids

**Figure 16:**
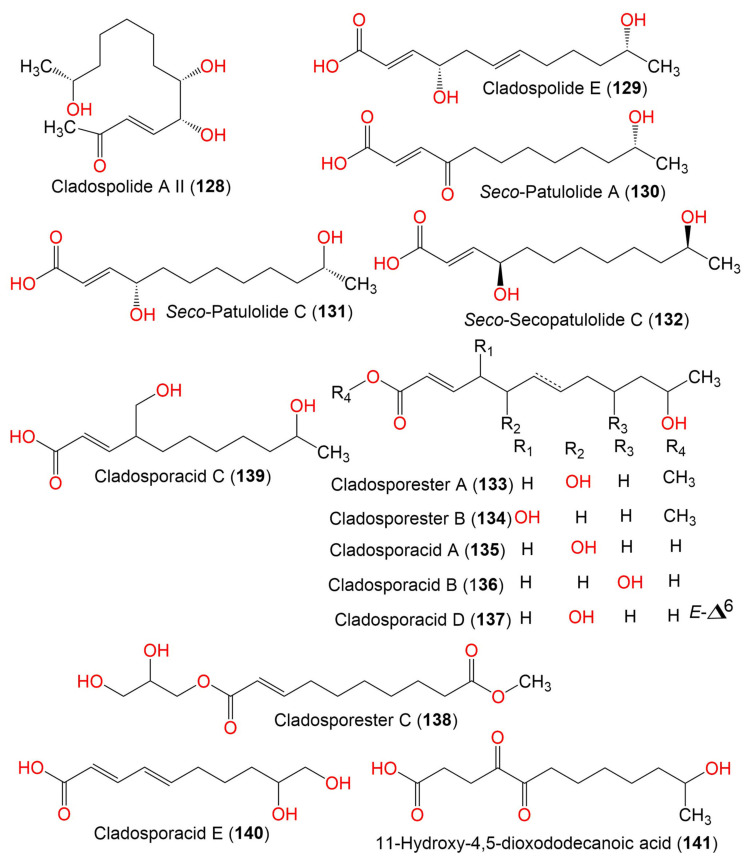
Seco-acids **128**–**141**.

The seco-acids **128**, **130**, and **141** isolated from *Cladosporium* sp. IFB3lp-2 EtOAc extract had no noticeable cytotoxicity versus HCT-116, Coxsachievirus A16, A549, MD-MBA-231, HepG2, human enterovirus 71, A375, and SW1116 cell lines (Conc. 20 µM) in the MTT assay [73]. Compound **131** did not show any anti-larval activity towards both *B. neritina* and *B. amphitrite* larvae [60]. Moreover, **132** did not have cytotoxic activity towards HCT-116, BEL-7402, HL-60, A549, L-02, HeLa, K562, MGC-803, MCF-7, PC-3, SH-SY5Y, and MDA-MB-231 (IC_50_ > 50 μM) [78], while it exhibited weak activity versus the aquatic pathogens *E. ictarda, E. tarda,* and *Cytospora glecosporioides* (MICs ranging from 16 to 32 μg/mL) [78]. Cladospolide E (**129**) separated from a soft coral-associated *Cladosporium* sp. TZP-29, together with the formerly isolated derivatives **130** and **131** had no cytotoxic effect towards A-549, SMMC-7721, and HeLa cells in the SRB method. Moreover, **129**–**131** with IC_50_ ranged from 7.1 to 13.1 µM remarkably reduced the accumulation of lipid elicited by oleic acid (OA) in the HepG2 liver cells, in comparison to lovastatin as determined by oil-red O staining and intracellular triglyceride (TG) and total cholesterol (TC) quantification (Figure 16).

Further, they exhibited potent lipid-lowering potential in HepG2 hepatocytes, revealing a promising anti-hyperlipidemic capacity [41]. The new fatty acid esters **133**, **134**, and **138** and new fatty acids **135**–**137**, **139**, and **140** isolated from *C*. *cladosporioides* OUCMDZ-187 obtained from the mangrove plant *Rhizophora stylosa* collected in Shankou, Guangxi Province of China showed no cytotoxic effects (IC_50_ > 50 μM) towards K562, A549, and HeLa cells in the SRB method [81]. Additionally, they revealed no antimicrobial activities (MIC > 150 μM) towards *S. aureus* CGMCC-1.2465, *E. coli* CGMCC-1.2389, *E. aerogenes* CGMCC-1.0876, *P. aeruginosa* CGMCC-1.1785, *B. subtilis* CGMCC-1.3376, and *C. albicans* CGMCC-2.2086 in the agar dilution method [81].

### 3.7. Tetralones (Napthalenones)

Tetralones comprise a bicyclic aromatic hydrocarbon and a ketone and are regarded as benzo-fused cyclohexanone derivatives. They played a substantial role as a starting material for the synthesis of a range of synthetic heterocyclic compounds and pharmaceuticals due to their potential reactivity and suitability [142]. Additionally, they are precursors of many natural metabolites and their derivatives. They have been used in the synthesis of therapeutically functional compounds such as antibiotics, acetylcholinesterase inhibitors, antidepressants, and antitumor alkaloids [142,143].

**Figure 17:**
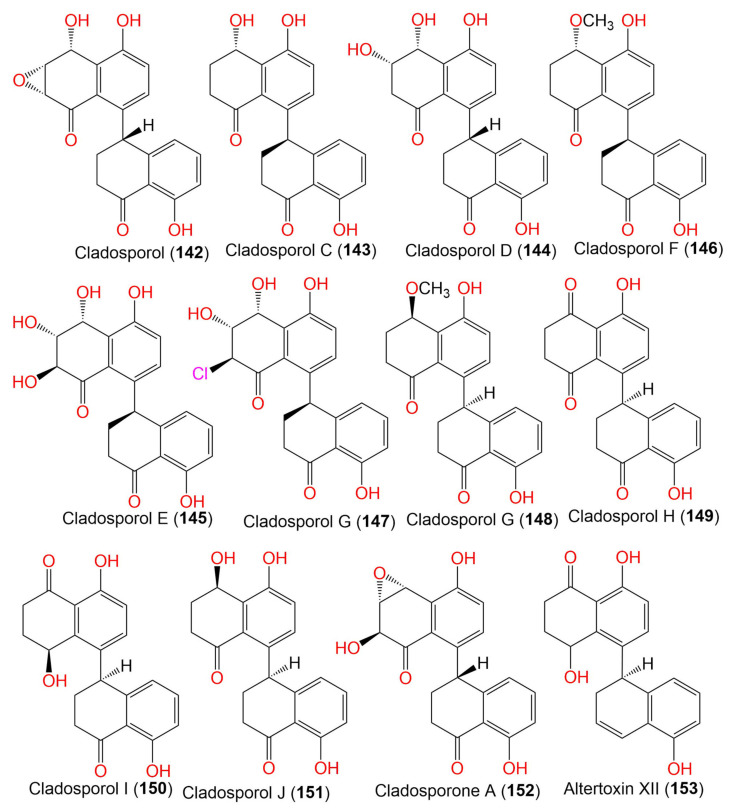
Tetralones (napthalenones) **142**–**153**.

Cladosporone A (**152**), a new dimeric tetralone bridged via C-C linkage, was separated from *Cladosporium* sp. KcFL6 derived from the mangrove plant *Kandelia candel*, together with **142**–**144** (Figure 17). In anti-COX-2 assay, **144** and **152** displayed COX-2 inhibitory activities (IC_50_ 60.2 and 49.1 μM, respectively), in comparison to NS-398 and indomethacin [82]. Moreover, none of these metabolites had antimicrobial activities against *A. baumannii* ATCC-19606, *S. aureus* ATCC-29213, *E. faecalis* ATCC-29212, *A. hydrophila* ATCC-7966, *E. coli* ATCC-25922, *K. pneumonia* ATCC-13883, *Fusarium* sp., *F*. *oxysporum* f. sp. *cucumeris*, *F.* o*xysporum* f. sp. *niveum*, *A. niger*, and *R. solani* in the disc diffusion assay [82].

Compounds **143** and **152** had moderate cytotoxic activity towards Huh-7, K562, HL-60, MCF-7, H1975, U937, A549, BGC823, MOLT-4, and HeLa cell lines (IC_50_ of **143** ranging from 11.4 to 72.5 µM and for **152** ranging from 10.1 to 53.7 µM), compared to trichostatin A in the trypan blue-cell viability assay [82]. Zurlo et al. reported that **142** had a remarkable anti-proliferative potential towards SW480, HT-29, and CaCo-2, in particular towards HT-29. It was revealed that HT-29 cells exposure to **142** produced G1/S phase cell cycle arrest, assisted by a vigorous p21^waf1/cip1^ expression, a significant down-regulation of CDK4, CDK2, cyclin E, and cyclin D1, and repression of CDK4 and CDK2 kinase activity [144]. It was demonstrated that its antiproliferative potential towards HT-29 cells was mediated via activation PPARγ, resulting in upregulation of p21^waf1/cip1^ expression and inducing degradation of β-catenin, as well as impairing TCF/β-catenin pathway as evident by reduced cyclin D1 and c-Myc transcription. Finally, it induced the expression of E-cadherin, therefore antagonizing invasion and metastasis [145]. *C*. *cladosporioides* HDN14-342 isolated from marine sediments yielded tetralone derivatives **143**, **145**–**147**, **154**, and **155** that were evaluated for cytotoxic activities towards HCT-116, HeLa, and A549 cell lines by SRB method and towards HL-60 and K562 cell lines by MTT method, in comparison to doxorubicin (IC_50_ 0.2–0.8 µM). Compounds **146** and **147** were active towards K562, HeLa, and HCT-116 cell lines (IC_50_ ranging from 3.9 to 23.0 µM), while other metabolites had no activity (IC_50_ > 50.0 µM) [83]. In 2020, He et al. reported that **143** possessed no anti-allergic effect (IC50 > 200 µM) on RBL-2H3 cells, in comparison to loratadine (IC50 35.01 µM) using fluorometric assay [75]. In 2017, Li et al. separated six cladosporol derivatives, cladosporol C (**143**) and cladosporols F-J (**146** and **148**–**151**) from the marine algal-derived *C. cladosporioides* EN-399 and evaluated their cytotoxic activities towards H446, A549, HeLa, L02, Huh7, LM3, SW1990, and MCF-7 using MTT assay. Note that **143**, **148**, and **149** displayed cytotoxic activities towards most of the tested cell lines with IC_50_ ranging from 1.0 to 20.0 μM. Notably, **149** had cytotoxic effect towards LM3, A549, and Huh7 cell lines (IC_50_ 4.1, 5.0, and 1.0 μM, respectively), compared to cisplatin (IC_50_ 1.3 μM for A549 and 9.1 μM for LM3) and fluorouracil (IC_50_ 6.2 μM for Huh7), whereas **143** exhibited cytotoxic activity (IC_50_ 4.0 μM) towards H446 cell line, compared to adriamycin (IC_50_ 4.0 μM). These results revealed that the existence of dihydro-1,4-naphthoquinone nucleus was important for the activity (**149** vs. **146**, **148**, and **143**, **150**, and **151**) and C-4 methoxyl strengthened the activity (**148** vs. **151**) [84].

**Figure 18:**
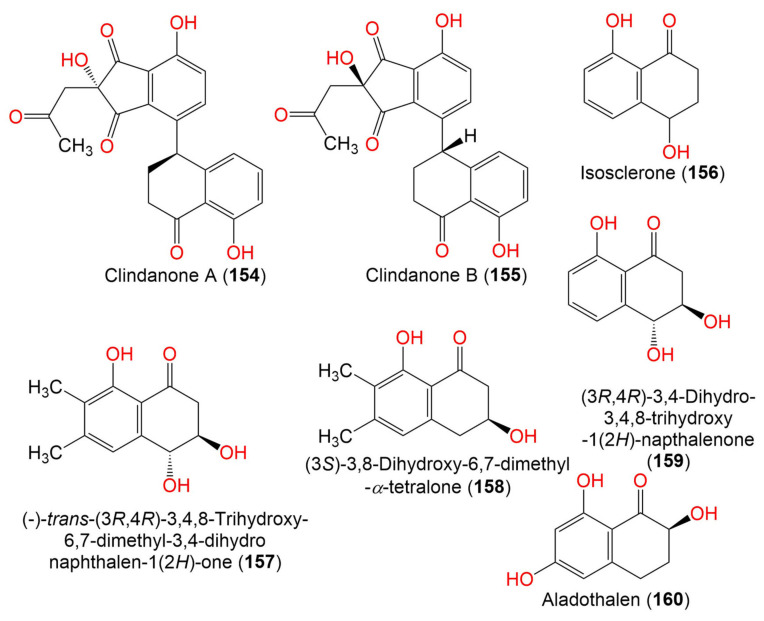
Tetralones (napthalenones) **154**–**160**.

Moreover, their antimicrobial potential was assessed versus *E. coli*, *A. hydrophila*, *S. aureus*, *E. tarda*, *P. aeruginosa*, *M. luteus*, *V. alginolyticus*, *V. parahemolyticus*, *V. harveyi*, *A. brassicae*, *F. oxysporum*, *G. graminis*, *C. gloeosporioides*, and *P. piricolav* using micro-plate assay. Compounds **143**, **146**, and **148**–**151** showed inhibitory potential towards *M. luteus*, *E. coli*, and *V. harveyi* (MICs 4–128 μg/mL). None of them had activity (MIC > 128 μg/mL) towards other tested microbes [84]. Bai et al. purified **143** and **145** from *Cladosporium* sp. JS1-2 isolated from the mangrove Ceriops tagal collected in the South China Sea [71]. Compound **145** prohibited the growth of Helicoverpa armigera Hubner newly hatched larvae (IC_50_ 150 μg/mL), compared to azadirachtin (IC_50_ 25 μg/mL) [71]. Further, they showed antibacterial potential versus *S. aureus* with MIC 6.25 and 1.56 μg/mL, respectively, compared with ciprofloxacin (MIC 0.39 μg/mL) [71]. Cladosporium sp. KFD33 isolated from blood cockle collected from Haikou Bay produced **150** and **153** that exhibited quorum sensing inhibitory potential towards *Chromobacterium violaceum* CV026 (MICs 30 and 20 µg/well, respectively) in the well diffusion assay [85]. Nevertheless, **156** had no observable cytotoxic activity towards SF-268, NCI-H460, MCF-7, and HepG-2 (conc. 100 μM) in the SRB assay [87]. The new naphthalenone derivative **157**, in addition to **156**, **158**, and **159** isolated *Cladosporium* sp. JJM22 associated with the mangrove plant *C. tagal* had no cytotoxic effect (IC_50_ > 10 μM) versus HeLa cell line in the MTT assay, compared to epirubicin [88] (Figure 18). In the micro-plate assay, only **158** exhibited noticeable antibacterial potential towards *S. aureus*, *B. cereus*, *E. coli*, *V. alginolyticus*, *V. parahemolyticus*, and MR *S. aureus* (conc. 20 μM) [88]. One new tetralone derivative, aladothalen (**160**) and previously reported (3S,4S)-3,4,8-trihydroxy-1-tetralone (**159**) were isolated from a sediment-associated *Cladosporium* sp. HDN17-58 (Figure 17). Note that **160** possessed potent bacteriostatic potential versus *Mycobacterium phlei*, *B. cereus*, and MRCNS (methicillin-resistant coagulase-negative Staphylococci) (MIC values of 25, 50, and 25 µM, respectively), compared to ciprofloxacin [89].

### 3.8. Perylenequinones

Perylenequinones comprise a class of natural products characterized by an oxidized pentacyclic core. They are dark-colored pigments isolated from diverse sources such as mold species, plants, and aphids [146]. They reported to have anthelmintic, photoactivity, antiviral and antitumor [146].

**Figure 19:**
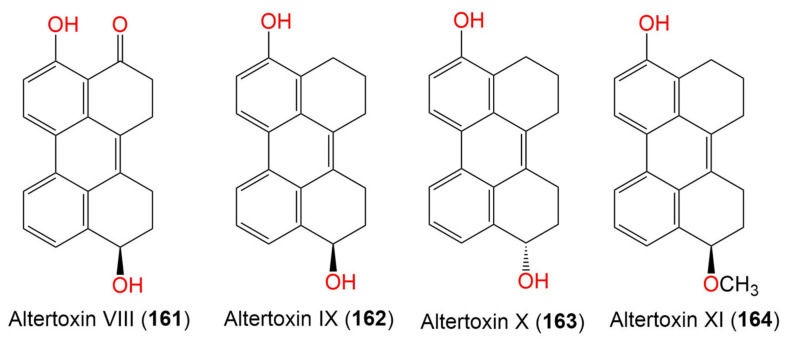
Perylenequinone **161**–**164**.

Four new perylenequinone derivatives, altertoxins VIII–XI (**161**–**164**), were isolated from *Cladosporium* sp. KFD33 (Figure 19). They exhibited quorum sensing inhibitory potential towards *C. violaceum* CV026 with MICs ranging from 20 to 30 µg/well in the well diffusion assay [85]. Structurally, these metabolites related to altertoxins I–III previously were reported from *Alternaria alternata* [147].

### 3.9. Naphthalene Derivatives

Naphthalenes are a class of arenes containing two *ortho*-fused benzene rings that have been reported from plants, liverworts, fungi, and insects [148]. Their derivatives exhibited anti-inflammatory, antimicrobial, antioxidant, anti-protozoal, cytotoxic, and anti-platelet aggregation activities [148].

**Figure 20:**
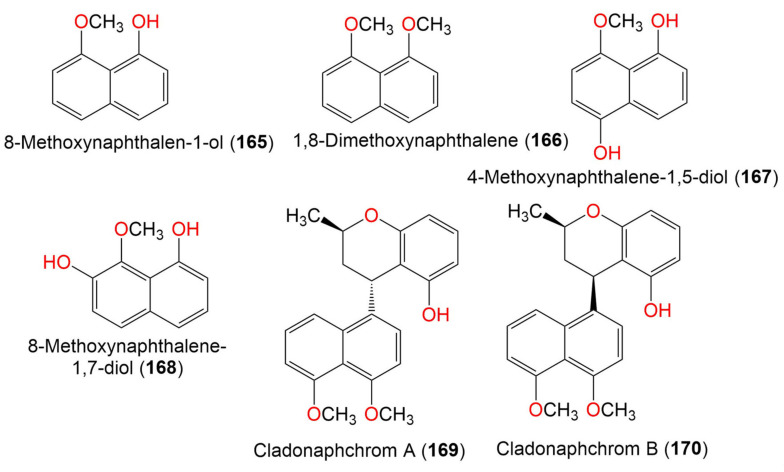
Naphthalene derivatives **165**–**170**.

*Cladosporium* sp. associated with the mangrove *C. tagal* biosynthesized the naphthalene derivatives **166**–**168** that had anti-inflammatory potential via in-vitro inhibition of induced NO (nitric oxide) production by LPS (lipopolysaccharide) in RAW264.7 cells [91] (Figure 20). The mangrove-associated fungus *Cladosporium* sp. JJM22 yielded new naphthalene-chromane derivatives, cladonaphchroms A (**169**) and B (**170**), and related metabolites **165** and **168** that were assessed for antibacterial effectiveness versus *S. albus* ATCC-8799, *E. coli* ATCC-25922, *B. subtilis* ATCC-6633, *Micrococcus tetragenus* ATCC-13623, and *M*. *luteus* ATCC-9341, employing microplate assay. Compound **169** possessed significant potential against *S. albus* (MIC 1.25 µg/mL), compared to ciprofloxacin (MIC 0.6 µg/mL). Moreover, **169** and **170** demonstrated broad-spectrum antifungal activities (MICs 25.0–100.0 µg/mL) towards *P. parasitica* var. *nicotianae*, *A. brassicicola*, *B. oryzae*, *C. capsici*, *C. paradoxa* Moreau, and *D. medusaea* Nitschke, compared to pochloraz (MICs 12.5–50.0 µg/mL) [90]. Wu et al. stated that **166** had no cytotoxic effect (IC_50_ > 10 μM) versus HeLa cell line in the MTT assay and no antibacterial activity towards *S. aureus*, *B. cereus*, *E. coli*, *V. alginolyticus*, *V. parahemolyticus*, and MR *S. aureus* (conc. 20 μM) in the microplate assay [88].

### 3.10. Xanthones

Xanthones are secondary metabolites commonly reported from plants, fungi, and lichen [149]. They are heterocyclic metabolites with a xanthene-9-one framework, which is connected to different functional groups: methoxy, hydroxyl, prenyl, and dihydrofuran [150]. These metabolites showed diverse bioactivities: anti-HIV, anti-leishmanial, antitumor, anti-quorum sensing, antimicrobial, anti-inflammatory, antimalarial, advanced glycation end-products inhibitory, antioxidant, antihypertensive, and cytotoxic [150,151].

**Figure 21:**
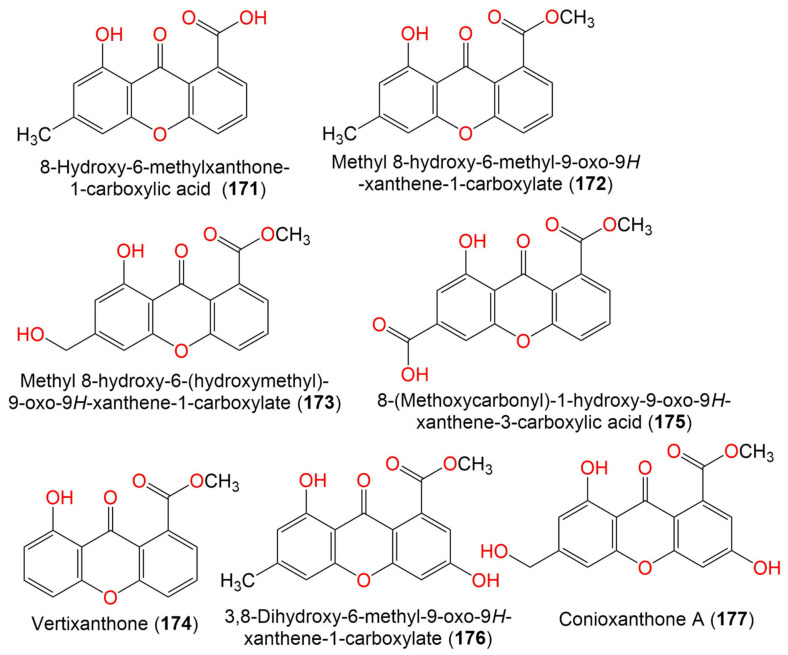
Xanthones **171**–**177**.

*C*. *halotolerans* GXIMD 02502 associated with the coral *Porites lutea* yielded compounds **171**–**177** that were evaluated for their cytotoxicity versus 22RV1 and C4-2B (prostatic cancer cell lines), as well as RWPE-1 (normal prostate epithelial cell). Among them, **171**–**173**, **175**, and **176** revealed notable cytotoxicity versus C4-2B and 22RV1 cells (inhibitions ranged from 55.8% to 82.1% at conc. 10 µM), whereas **176** was the potent one (inhibitions 77.7% and 82.1%, respectively). On the other hand, they exhibited nearly no cytotoxic effect versus RWPE-1 cell (inhibition < 27% at conc. 10 µM) [92] (Figure 21).

### 3.11. Tropolones

Tropolones are natural metabolites with a cyclohepta-2,4,6-trienone moiety [152]. They are known to be produced by fungi, bacteria, and plants. It was reported to display diverse bioactivities, including antimicrobial, antiviral, anti-HIV, hepatitis, anti-inflammatory, and anticancer [152].

**Figure 22:**
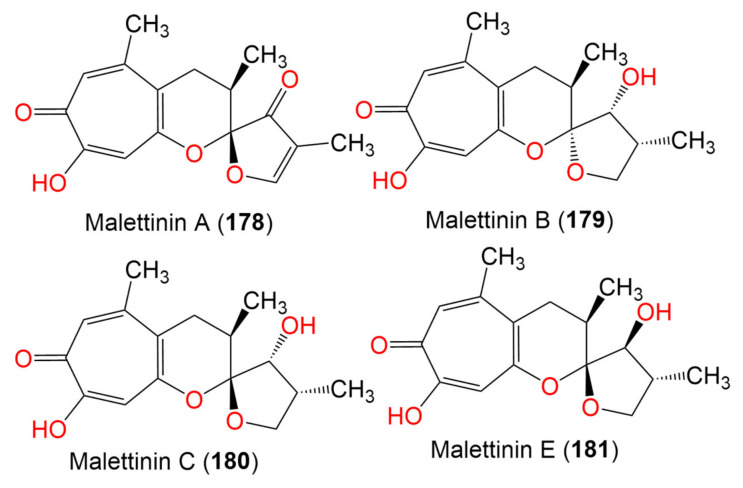
Tropolones **178**–**181**.

Silber et al. reported the isolation of malettinins A–C (**178**–**180**), along with the new metabolite, malettinin E (181) from *Cladosporium* sp. strain KF501 isolated from the German Wadden Sea (Figure 22). These metabolites have dihydropyran/tropolone structures connected to a furan ring. The configuration of 181 was determined by the single-crystal X-ray diffraction method. Interestingly, this was the first report for tropolones isolation from genus *Cladosporium*. They were evaluated for antimicrobial activity towards *X. campestris*, *B. subtilis*, *S. epidermidis*, *C. albicans*, and *Trichophyton rubrum* using the microplate assay. Note that **178**–**181** exhibited weak antifungal potential towards *Trichophyton rubrum* (IC_50_ 30.7–83.2 μM), whereas **179**–**181** exhibited weak antibacterial effect towards *Xanthomonas campestris* (IC_50_ 28.3–37.9 μM), compared to chloramphenicol (IC_50_ 2.1 μM) [93].

### 3.12. Binaphthopyrones

Bisnaphthopyrones are dimers, belonging to naphthopyrones. They have C13 basic skeleton (C6-C4-C3) that consists of naphthalene and pyrone cores [153].

**Figure 23:**
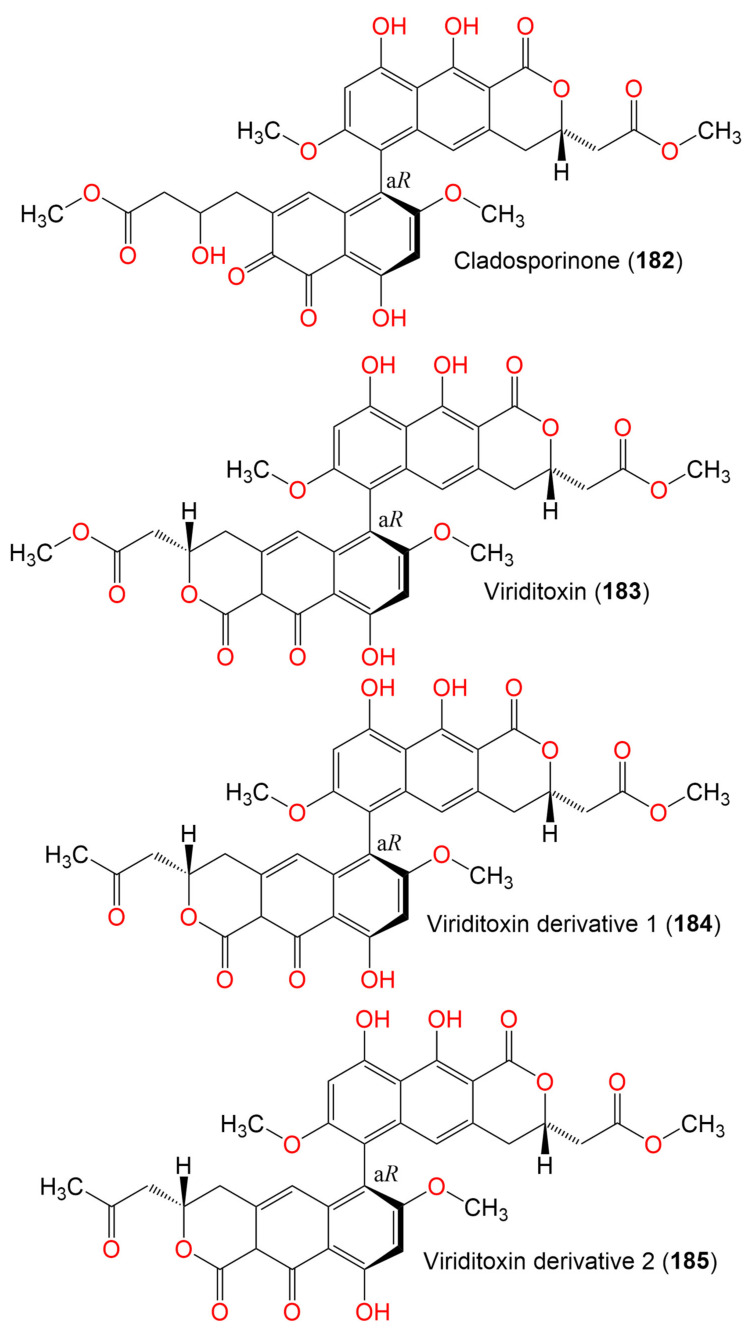
Binaphthopyrones **182**–**185**.

The new binaphthopyrone, cladosporinone (**182**), and the formerly isolated viriditoxin (**183**) and viriditoxin derivatives (**184** and **185**) were separated the sediment associated *C. cladosporioides* (Figure 23). Note that **183** was firstly reported from *Aspergillus viridinutans* [154]. They were assayed for their cytotoxic potential versus L5178Y cells in the MTT assay. Compound **183** was the most potent one (IC_50_ 0.1 μM), however **182** and **184** had a cytotoxic effect (IC_50_ 0.88 and 0.25 μM, respectively). However, **185** was ineffective [94]. Note that all metabolites had selective potential towards *S. aureus* ATCC-29213, with **183** being the most effective (MIC 0.023 μM) [94].

### 3.13. Benzopyranes, Benzopyrones, and Pyrones

**Figure 24:**
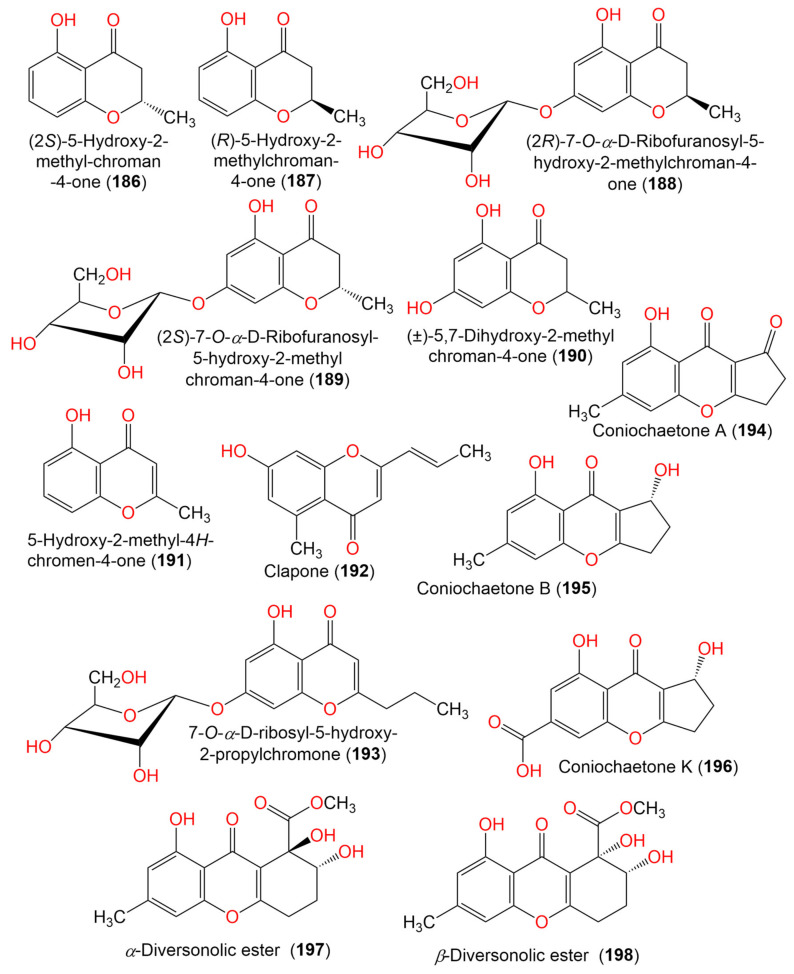
Benzopyrone derivatives **186**–**198**.

Wang et al. reported the separation of compounds **188**–**190**, **193**, **200**, **201**, and **203** from *Cladosporium* sp. OUCMDZ-302 isolated from mangrove plant *Excoecaria agallocha*. They possessed no cytotoxic effect towards BEL-7402, A549, HeLa, K562, HL-60, and H1975 cell lines in the MTT and SRB methods. Whilst **201** and **203** showed radical scavenging activity against DPPH (IC_50_ 5.66 and 6.67 µM, respectively). None of these metabolites exhibited antimicrobial activities against *E. coli*, *E. aerogenes*, *P. aeruginosa*, *B. subtilis*, and *C. albicans* [95]. The newly isolated benzopyrone, clapone (**192**), had no α-glycosidase inhibitory effect and no cytotoxic activity towards SGC-7901, K562, Hela, and BEL-7042 cell lines in the MTT assay [67]. Furthermore, **186** and **205** displayed no cytotoxic effect (IC_50_ > 10 μM) versus HeLa cell line in the MTT assay, as well as no antibacterial potential towards *S. aureus*, *B. cereus*, *E. coli*, *V. alginolyticus*, *V. parahemolyticus*, and MR *S. aureus* (conc. 20 μM) in the microplate assay [88]. *C*. *halotolerans* GXIMD 02502 associated with the coral *Porites lutea* yielded a new benzopyranone derivative, coniochaetone K (**196**) with unusual C-8 carboxyl, along with **194**, **195**, **197**, and **198** that were evaluated for their cytotoxicity versus 22RV1, C4-2B, and RWPE-1 cell lines (Figure 24).

**Figure 25:**
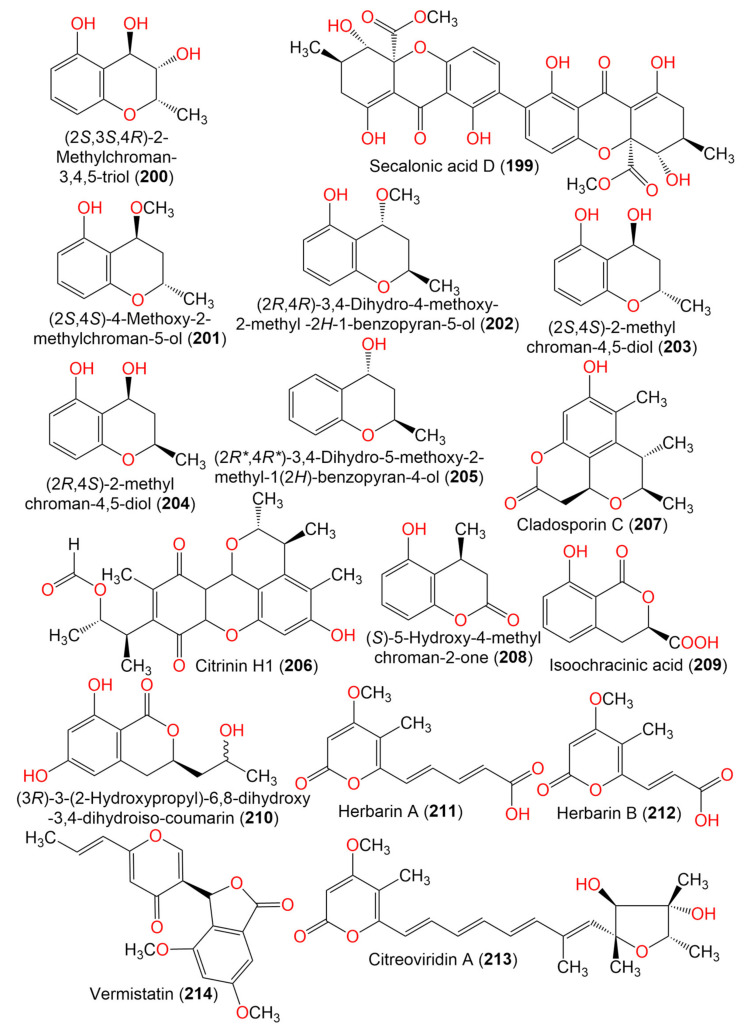
Benzopyrone **199**–**210** and pyrone (**211**–**214**) derivatives.

Among them, **194** and **196** revealed notable cytotoxicity versus 22RV1 cells (inhibition 67.4% and 64.6%, respectively, at conc. 10 µM). On the other hand, they exhibited nearly no cytotoxic effect versus RWPE-1 and C4-2B cells [92]. Bai et al. reported that **206** prohibited the growth of H. armigera Hubner newly hatched larvae (IC_50_ 100 μg/mL), compared to azadirachtin (IC_50_ 25 μg/mL) [71]. Further, it showed moderate antibacterial potential versus *S. aureus* (MIC 6.25 μg/mL), compared with ciprofloxacin (MIC 0.39 μg/mL) [71]. Cladosporin C (**207**) did not have obvious anti-biofilm activity towards *S. aureus*, *E. coli*, and *B. subtilis* [36]. On the other hand, it showed moderate toxicity towards brine shrine naupalii (LC_50_ 49.9 µM), compared to toosendanin (LC_50_ 21.2 µM) in the brine shrimp lethality assay [36]. Furthermore, **210** possessed no anti-allergic effect (IC_50_ > 200 µM) on RBL-2H3 cells, in comparison to loratadine (IC_50_ 35.01 µM) using fluorometric assay [75]. α-Pyrone derivatives **211**–**213** were separated from *C. herbarum* isolated from the sponge *Aplysina aerophoba* (Figure 25). Compounds **211** and **212** had activity towards *Artemia salina* (conc. 100 µg and 50 µg) with mortality rates 85 and 75% and 80 and 65%, respectively, while **213** did not have any activity. Besides, **213** showed growth inhibitory activity towards *Spodoptera littoralis* larvae (7 and 33% at conc. 250 and 100 ppm, respectively) [96]. However, **211**–**213** did not show any noticeable antimicrobial activity in the agar plate diffusion assay [96].

### 3.14. Lactones, Cyclohexene, and Azaphilone Derivatives

**Figure 26:**
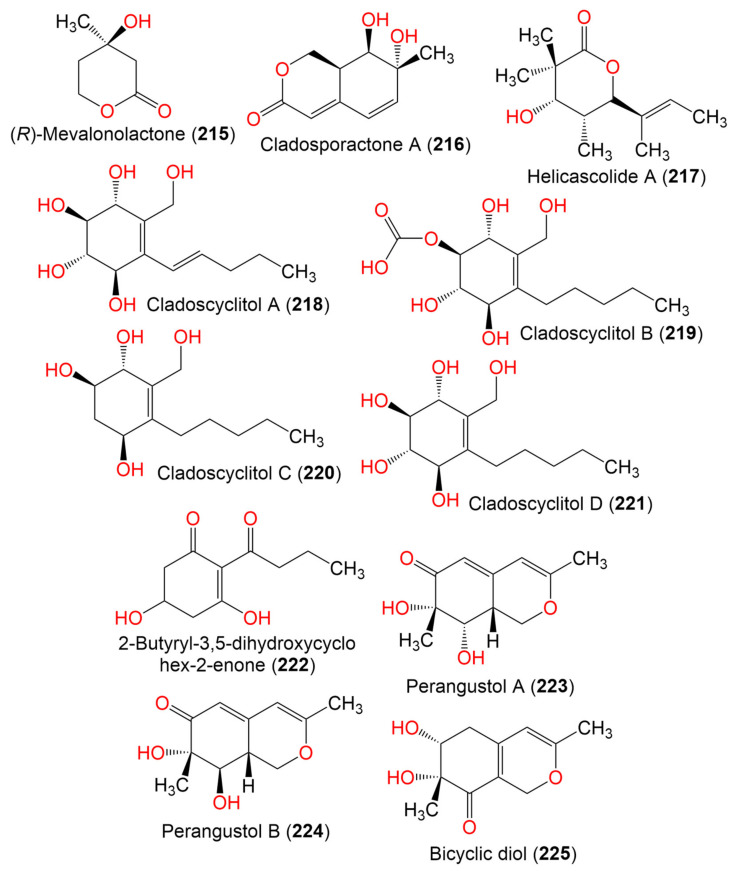
Lactone (**215**–**217**), cyclohexene (**218**–**222**), and azaphilones (**223**–**225**) derivatives.

In 2020, He et al. purified **216** from *C. cladosporioides* that possessed no anti-allergic effect (IC_50_ > 200 µM) on RBL-2H3 cells, in comparison to loratadine (IC_50_ 35.01 µM) using fluorometeric assay [75]. The mangrove plant *C. tagal* associated-fungus *Cladosporium sp*. JJM22 produced new cyclohexene derivatives, cladoscyclitols A–D (**218**–**221**) (Figure 26). Compound **219** (IC_50_ 2.95 μM) revealed potent *α*-glucosidase inhibitory activity, compared to acarbose (IC_50_ 2.35 μM) in the colorimetric assay [97]. On the other hand, it had no antimicrobial potential towards *S. aureus* ATCC-6538, *E. coli* ATCC-25922, *B. cereu* ATCC-6633, *V. alginolyticus* ATCC-3787, *V. Parahemolyticus* ATCC-17802, or MRSA CMCC-B-63303 in the micro-plate assay [97]. Perangustols A (**223**) and B (**224**), representing new azaphilone epimers, together with bicyclic diol (**225**) were separated from sea sediment-associated *C. perangustum* FS62 fungus. They had no observable cytotoxic activity towards SF-268, NCI-H460, MCF-7, and HepG-2 (Conc. 100 μM) in the SRB assay [87].

### 3.15. Phenolics and Other Aromatic Compounds

**Figure 27:**
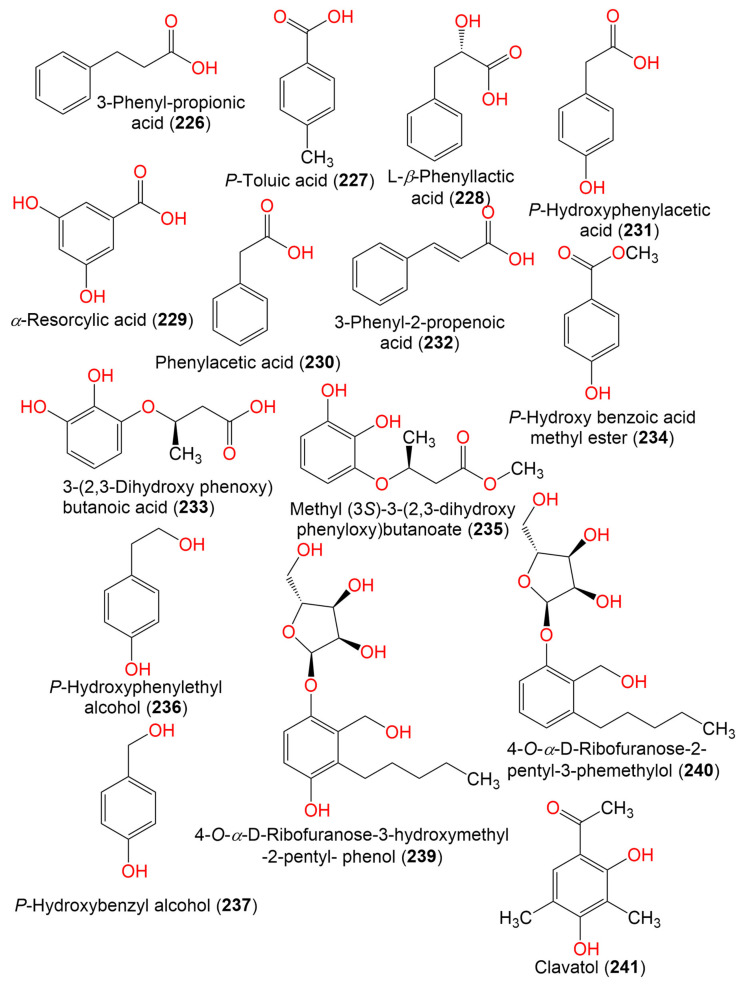
Phenolics **226**–**241**.

In the DPPH assay, **233** and **235** showed DPPH radical scavenging activity (IC_50_s 0.24 and 2.65 µM, respectively), in comparison to ascorbic acid (IC_50_ 3.29 µM). Further, none of these compounds had antimicrobial potential versus *P. aeruginosa*, *E. aerogenes*, *B. subtilis*, *E. coli*, and *C. albicans* [95]. The metabolites **232**, **238**, and **249** were separated from EtOAc extract of *Cladosporium* sp. F14 isolated from seawater and investigated for their anti-larval activity (conc. 50 µg/mL) towards *B. neritina* and *B. amphitrite* larvae in the settlement inhibition assays [60] (Figure 27). Compound **232** had weak larvae settlement inhibition towards *B. neritina* and *B. Amphitrite*, respectively, whereas **238** and **249** showed weak inhibitory effects towards *B*. *amphitrite* and *B*. *neritina* larvae, respectively. In another larval settlement bioassay, **232**, **238**, and **249** inhibited *B*. *neritina* larval settlement (EC_50_ 11.51, 102.23, and 77.85 µg/mL, respectively) and *B*. *amphitrite* larval settlement (EC_50_ 84.28, 53.65, and 9.18 µg/mL, respectively). The larval settlement EC_50_ values of **249** towards *B*. *amphitrite* and **232** towards *B*. *neritina* were less than the US Navy program established standard requirement (EC_50_ 25.0 µg/mL), revealing the potential of **232** and **249** as antifouling agents [60]. Furthermore, **232** obviously prohibited *L. hongkongensis* growth (IZD 8 mm and MIC 80 µg/mL), compared to streptomycin (MIC 250 µg/mL) [60]. The ribofuranose phenol derivative, **239** isolated *Cladosporium* sp. JJM22 associated with the mangrove plant *C. tagal* had no cytotoxic effect (IC_50_ > 10 μM) versus HeLa cell line in the MTT assay, compared to epirubicin [88].

**Figure 28:**
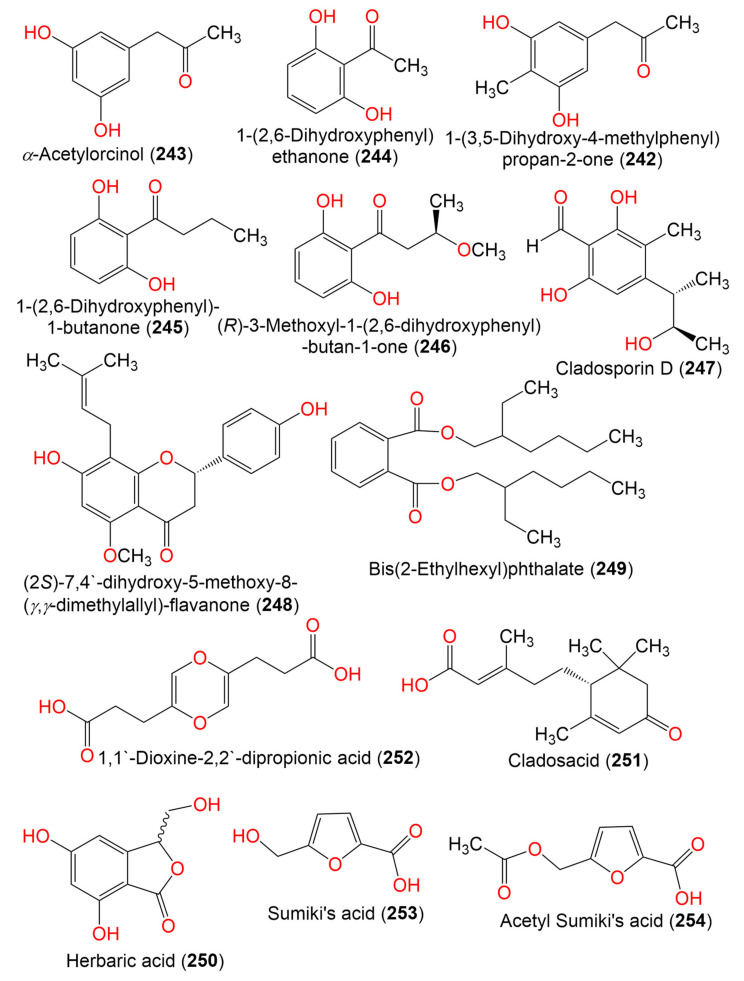
Phenolics **242**–**248** and others **249**–**254**.

Additionally, it exhibited no noticeable antibacterial potential towards *S. aureus*, *B. cereus*, *E. coli*, *V. alginolyticus*, *V. parahemolyticus*, and MR *S. aureus* (conc. 20 μM) in the microplate assay [88]. The new ribofuranose phenol derivative, **240** (IC_50_ 2.05 μM) revealed potent *α*-glucosidase inhibitory activity, compared to acarbose (IC_50_ 2.35 μM) in the colorimetric assay [97]. On the other hand, it had no antimicrobial potential towards *S. aureus* ATCC-6538, *E. coli* ATCC-25922, *B. cereus* ATCC-6633, *V. alginolyticus* ATCC-3787, *V. Parahemolyticus* ATCC-17802, and MRSA CMCC-B-63303 in the microplate assay [97]. Phytochemical investigation of the mycelium extract of the marine-derived fungus *Cladosporium* sp. associated with *Chondria crassicualis* red alga resulted in the separation of a phenol derivative, clavatol (**241**) that exhibited antioxidant capacity (ED_50_ 227.0 µM) more than oxybenzone (sunscreen agent, ED_50_ 350 µM) as evident by their UV-A protecting potential [65]. On the other hand, it was inactive towards MDRSA, MRSA, and *S*. *aureus* [65]. Fan et al. stated that compounds **242** and **243** exhibited no observable cytotoxic activity towards SF-268, NCI-H460, MCF-7, and HepG-2 (Conc. 100 μM) in the SRB assay [87] (Figure 28). Cladosporin D (**247**) did not have obvious anti-biofilm activity towards *S. aureus*, *E. coli*, and *B. subtilis* [36], while it exhibited significant antioxidant activity (IC_50_ 16.4 µM), compared with ascorbic acid (IC_50_ 4.9 µM). Besides, it showed moderate toxicity towards brine shrine naupalii (LC_50_ 81.4 µM), comparing with toosendanin (LC_50_ 21.2 µM) in the brine shrimp lethality assay [13].

Compound **248** separated from *Cladosporium* sp. TPU1507 derived from marine sponge and assessed for inhibitory effect towards PTP1B and TCPTP using enzyme-based assay [68]. It showed an inhibitory effect on TCPTP (IC_50_ 27 μM) that was 2-fold weaker than on PTP1B (IC_50_ 11 μM) [68]. The new phthalide, herbaric acid (**250**), separated from *C. herbarum* isolated from *Callyspongia aerizusa* had no activity towards *A. salina* and HL-60 human leukemia cell line [96]. In addition, the newly separated abscisic acid analog **251** from *Cladosporium* sp. OUCMDZ-1635 possessed no cytotoxic effect towards MCF-7, HeLa, HCT-116, HeLa, HCT-116, K562, and HL-60. Furthermore, it did not show antibacterial activity (conc. 100 μg/mL) against *B. subtilis*, *P. aeruginosa*, *C. perfringens, S. aureus, E. coli*, and *C. albicans* [56]. The new pentenoic acid derivative, 1,1′-dioxine-2,2′-dipropionic acid (**252**) prohibited the growth of H. armigera Hubner newly hatched larvae (IC_50_ 150 μg/mL), compared to azadirachtin (IC_50_ 25 μg/mL) [71]. Further, it showed moderate antibacterial potential versus *S. aureus* (MIC 25.0 μg/mL), compared with ciprofloxacin (MIC 0.39 μg/mL) [71]. The furan carboxylic acid metabolites, Sumiki’s acid (**253**) and acetyl Sumiki’s acid (**254**) exerted activity towards *S. aureus* and *B. subtilis* (IZDs 7 mm at conc. 5 µg/disk), whereas they had no activity towards *C. albicans* and *E. coli* [74].

### 3.16. Sterols and Terpenes

**Figure 29:**
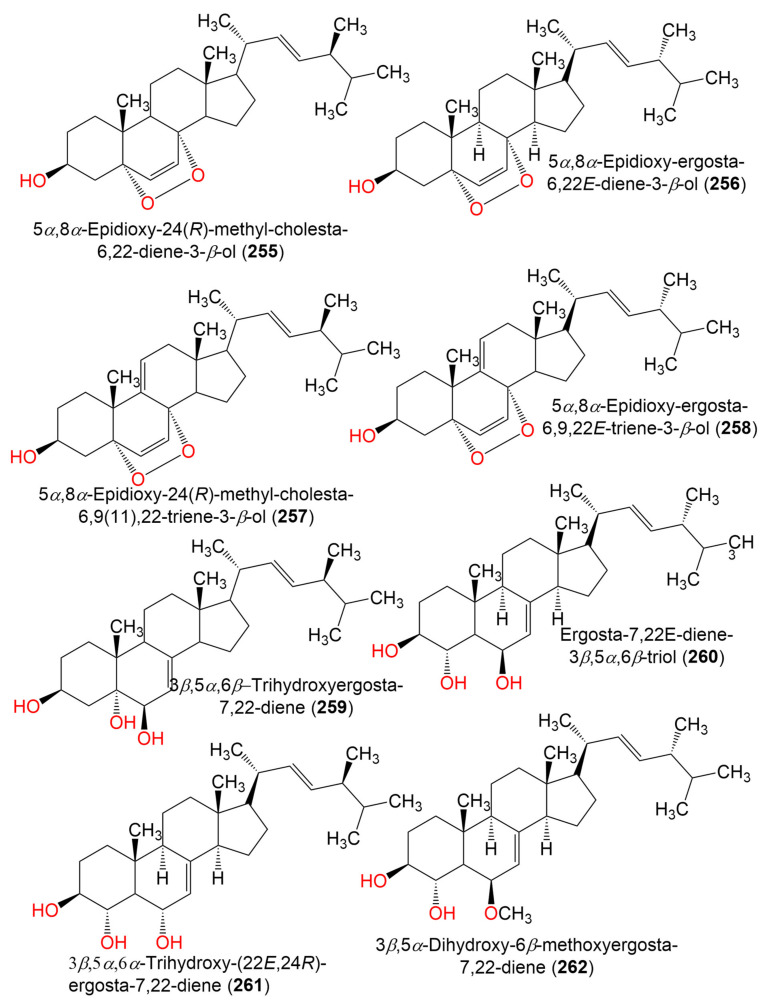
Sterols **255**–**262**.

**Figure 30:**
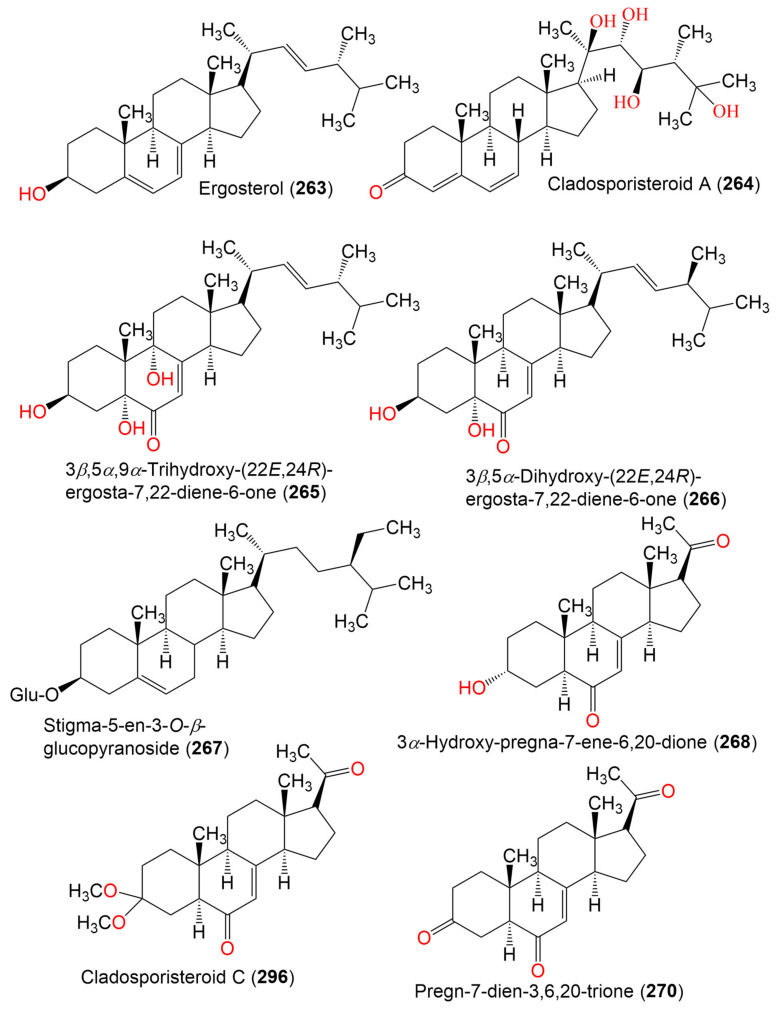
Sterols **263**–**267** and terpenes **268**–**270**.

A study conducted by Yu et al. in 2018 led to the separation of a new pregnane; 3α-hydroxy-7-ene-6,20-dione (**268**) and six sterol derivatives: **256**, **258**, **260**, **262**, **263**, and **267** from gorgonian-associated *Cladosporium* sp. WZ-2008-0042 [100]. Note that **268** was reported in the same year by Pang et al. as new metabolites with the name cladosporisteroid B from *Cladosporium* sp. SCSIO41007 associated with *Callyspongia* sp. [61]. These metabolites (IC_50_ values ranging from 0.11 to 0.17 µM) revealed antiviral activity against RSV (respiratory syncytial virus) with therapeutic ratio (TC_50_/IC_50_) values ranging from 5.18 to 9.92, in comparison to ribavirin in the neuraminidase inhibition assay. This could be due to their binding to RSV GREs (glucocorticoid response elements) [100]. Moreover, they (conc. 0.1 mg/mL) displayed weak to moderate AChEI potential, in comparison to huperzine A and galanthamine using the modified Ellman’s method [100]. Further, **268** had no noticeable antibacterial potential towards *B. cereus*, *M. luteus*, *S. aureus*, *V. anguillarum E. coli*, *Shigella dysenteriae*, *B. subtilis*, and *V. Parahemolyticus*, while **263** was moderately active (MIC 3.13 μM) towards *S. dysenteriae* [100]. In 2020, He et al. reported that **256**, **261**, **265**, **266**, and **268** separated from *C. cladosporioides* sea sediment-derived fungus possessed no anti-allergic effect on RBL-2H3 cells, in comparison to loratadine using fluorometeric assay [75] (Figure 29 and Figure 30). In 2018, Pang et al. separated new sterol cladosporisteroid A (**264**) and new pregnanes, cladosporisteroid B (**268**) and cladosporisteroid C (**269**), along with **259**, **265**, and **270** from *Cladosporium* sp. SCSIO41007 isolated from *Callyspongia* sp. and assessed their antiviral activity towards EV71 and H3N2 using CCK-8 and CPE assays, respectively. Only, **268** (IC_50_ 16.2 μM) had weak activity towards H3N2 compared to oseltamivir (IC_50_ 34.0 nM). Moreover, they revealed no cytotoxic effect towards K562, MCF-7, and SGC-7901 in the CCK-8 assay [61]. Additionally, **268** was purified from *C. sphaerospermum* EtOAc fraction by HPLC with the aid of LCMS and assessed for its influence on adipogenesis and lipid metabolism during maturation of adipocyte (Conc. 1.25, 2.5, 5, and 10 μM) using 3T3-L1 preadipocytes [101]. It substantially prohibited lipid accumulation and differentiation of 3T3-L1-preadipocytes into adipocytes, leading to reducing *Adipsin* (adipocyte marker gene) expression. Further, it significantly upregulated *ATGL* (lipolytic gene, Conc. 5 and 10 μM) and reduced *FASN* and *SREBP1* (lipogenic genes, conc. 1.25, 2.5, 5, and 10 μM) expression. Collectively, 268 facilitated lipid metabolism and disrupted adipogenesis via promoting lipolysis and prohibiting lipogenesis [101].

### 3.17. Alcohols and Aldehydes

**Figure 31:**
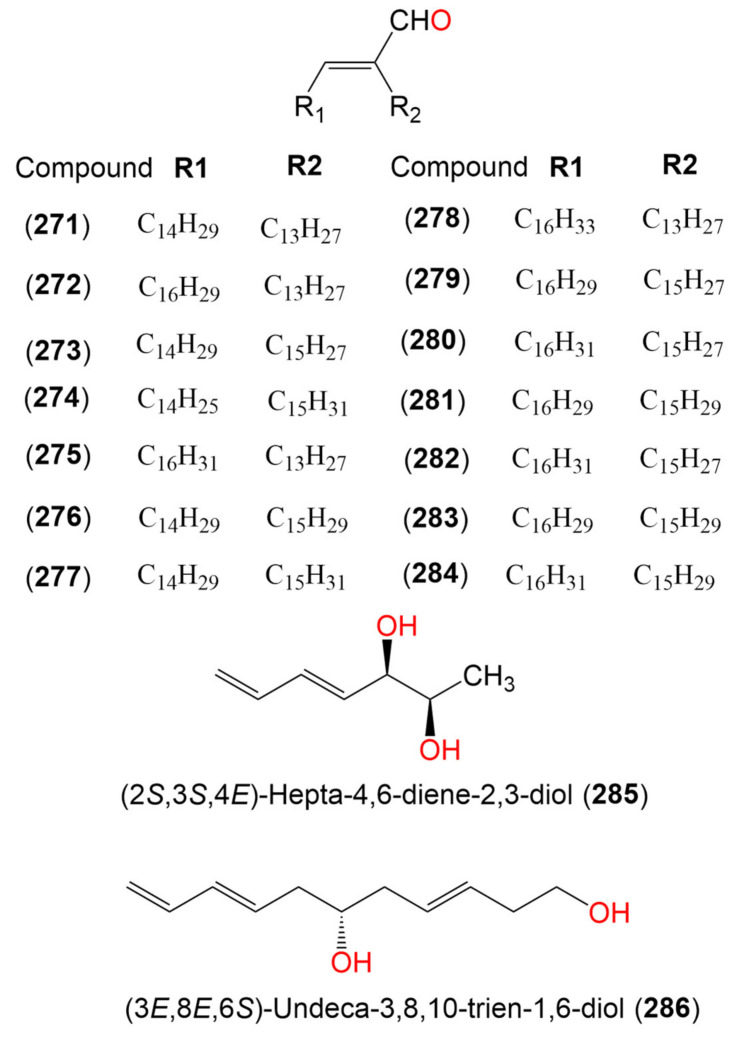
Aldehydes (**271**–**284**) and alcohols (**285** and **286**).

Gallo et al. reported for the first time from fungi the isolation of α,β-unsaturated aldehydes (**271**–**284**) from the culture of *Cladosporium* sp. isolated from intertidal marine sediment [102] (Figure 31). They exerted antimicrobial activity towards *E. coli* ATCC-25922, *B. subtilis* ATCC-6633, and *C. albicans* ATCC-18804 in the agar diffusion method. It is noteworthy that this class of metabolites had been reported formerly from red algae (e.g., *Corallina mediterranea* and *Laurencia papillosa*, *L. spectabilis*, and *L. undulata*) [155,156]. The new aliphatic alcohols, (2*S*,3*S*,4*E*)-hepta-4,6-diene-2,3-diol (**285**) and (3*E*,8*E*,6*S*)-Undeca-3,8,10-trien-1,6-diol (**286**) were assessed for cytotoxic potential versus HeLa, BEL-7402, HL-60, A549, K562, and H1975 cell lines. Compound **286** had a cytotoxic effect versus H1975 cell line (IC_50_ 10.0 µM), compared to ADR (IC_50_ 0.38 µM). While both metabolites revealed no antioxidant and antimicrobial capacities [95].

### 3.18. Bioactivities of Cladosporium Species Extracts

Ding et al. stated that *Cladosporium* sp. isolate N5 associated with *Porphyra yezoensis* red alga did not produce any pathogenic symptoms in the reinfection assay. Further, its EtOAc extract displayed no lethality to *A. salina* and had a moderate antimicrobial activity which indicated that *Cladosporium* sp. had no toxicity to the aquatic ecosystem and could be applied as a biocontrol agent [59]. In the disc diffusion method, *Cladosporium* sp. EIODSF 008 EtOAc extract exhibited significant antibacterial potential towards *E. coli*, *M. luteus*, and *B. subtilis* (conc. 100 µg/disc) [57]. The EtOAc extract of *Cladosporium* sp. EN-S01 isolated from *Sargassum cinereum* brown algae showed anticancer activity towards MCF-7, HeLa, and DU-145 cell lines (IC_50_ 8.46, 9.87, and 98.03 µg/mL, respectively). The extract had greater cytotoxic activity and anti-proliferative towards MCF-7 and HeLa cell lines than towards DU-145 [157]. Moreover, the EtOAc extract of *C*. *cladosporioides* KT384175 isolated from the seaweed *Sargassum wightii* possessed remarkable antioxidant potential that was comparable to ascorbic acid, as well as significant Fe^3+^ reducing power that could be referred to its phenolic contents. Moreover, it revealed anti-angiogenic potential as evidenced by the decrease in the number and length of blood vessel branches on CAM (chick chorioallantoic membrane) in-vivo in the CAM assay. Further, *C. cladosporioides* extract (conc. 1.0 mg/mL) had lower wound healing potential than thalidomide (conc. 1.0 µg/mL) in the in vitro scratch assay using MCF-7 cells [158]. The sea water-derived fungus *Cladosporium* sp. F14 can produce antifouling and antibiotic metabolites in the existence of xylose or glucose. Significantly, it showed higher antibiotic activity towards *M. luteus*, *P. piscida*, *Rhodovulum* sp., *Ruegeria* sp., *V. fluvialis*, and *V. harveyi* in the existence of a sugar carbon source than in its absence in the disc diffusion assay, even though the fungal cells were well-grown under both conditions. Moreover, it possessed antifouling potential as it reduced the attachment of *B. neritina* (bryozoan larvae) in the larval settlement assay [159]. The gold nanoparticles synthesized from *C. cladosporioides* isolated from the seaweed *S. wightii* possessed noticeable antimicrobial potential towards *E. coli* MTCC-118, *B. subtilis* MTCC-441, *S. aureus* MTCC-7443, *P. aeruginosa MTCC-424*, and *A. niger* MTCC-281 with the highest growth inhibition towards *S. aureus* (IZD 12 mm) and least activity against *B. subtilis* (IZD 9.5 mm), compared to ampicillin (IZDs 15 and 12 mm, respectively) in the well diffusion method. Furthermore, they also had significant antioxidant potential comparable to ascorbic acid in the DPPH assay and moderate effectiveness in reducing power assay [160]. Ameen et al. reported that the AgNPs synthesized from *C. halotolerans* biomass isolated from the marine debris collected around Tarout Island showed a significant free radical scavenging effect (%inhibition 78% within 30 min incubation) in the DPPH assay. Moreover, it exhibited cytotoxic potential towards MCF-7 (IC_50_ 34.27 µL/mL), compared to cisplatin (IC_50_ 17.69 µL/mL) in the MTT assay, as well as an antifungal effect against *A. niger* (%inhibition 70 and 45% at conc. 1000 and 500 ppm, respectively) in the broth dilution method [161].

From the comprehensive review of the available literature, it was noticed that *C. phlei* (causal agent of Timothy leaf spot disease) and *C. cucumerinum* (causal agent of scab disease of many Cucurbitaceae plants) were isolated mainly from plant sources [162,163,164,165]. These species produced perylenequinone derivatives as major metabolites which are responsible for pigmentation and discolorations of the leaves [162,165]. Additionally, cotylenins, plant growth regulators were isolated from an unidentified *Cladosporium* species [166,167,168,169,170]. However, tetralones, *seco*-acids, macrolides, diketopiperazines, alkaloids, and tetramic acid derivatives were reported mainly from marine-associated *Cladosporium* species.

## 4. Conclusions

**Figure 32:**
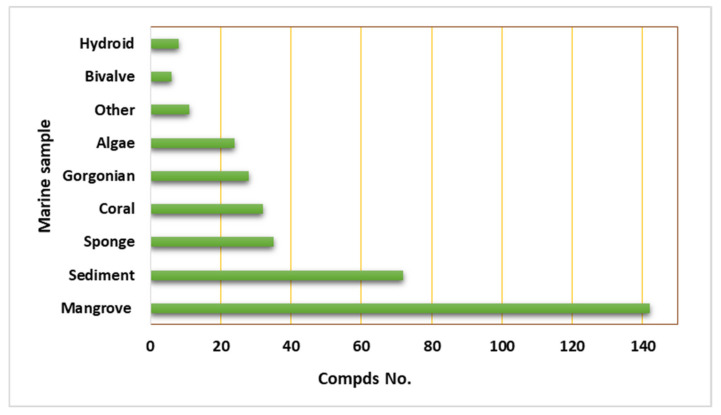
Number of compounds separated from *Cladosporium* species isolated from various marine samples.

Numerous structurally diverse biometabolites are discovered from marine-derived fungi that represent a rich library for the development of drug lead. Marine-associated *Cladosporium* species are of biotechnological and industrial relevance and could be considered as substantial enzyme producers. Their enzymes are active in harsh conditions such as extremely low temperatures and high salinity. Therefore, they can be utilized in various industrial and biotechnological applications. Besides, these species were found to be a wealthy pool covering a wide array of metabolites with various bioactivities. Over the past 22 years, 286 metabolites have been separated from marine-associated *Cladosporium* species isolated from various marine samples, including mangrove, sediment, sponges, corals, gorgonians, algae, bivalves, hydroids, and others (Figure 32).

**Figure 33:**
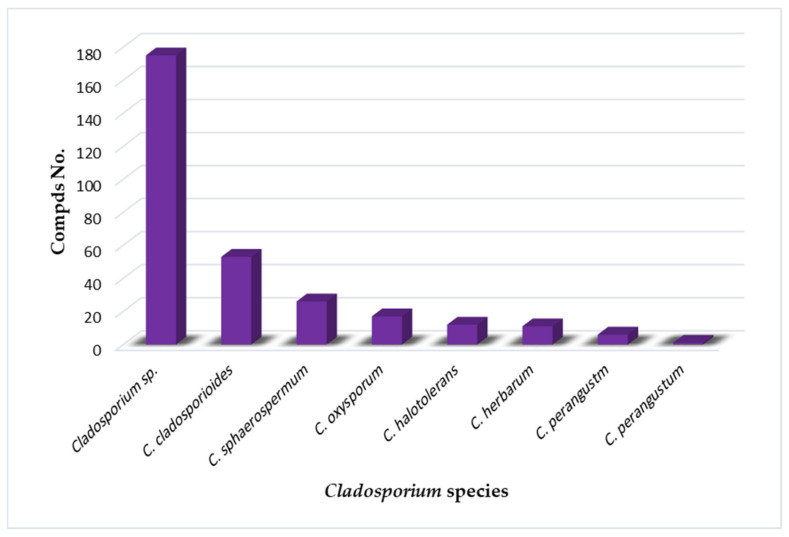
Number of compounds separated from marine-derived *Cladosporium* species.

More than 75% of these metabolites have been reported from unidentified *Cladosporium* sp. (175 metabolites, 61%) and *C. cladosporioides* (53 metabolites, 18.5%) (Figure 33).

**Figure 34:**
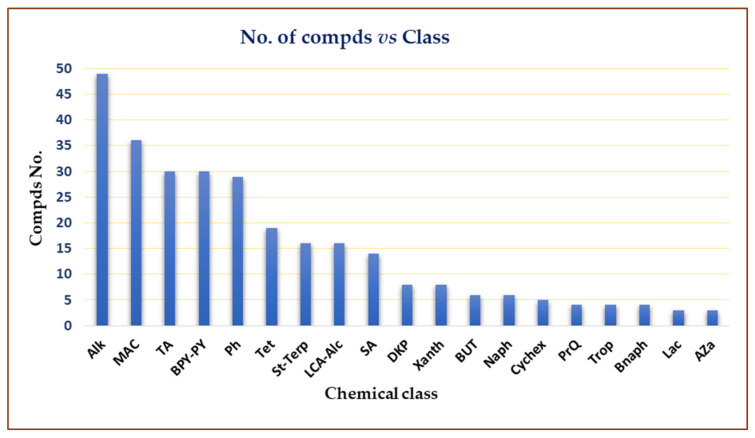
Number of metabolites from each class of natural products.

The results revealed that alkaloids, macrolides, tetramic acid and pyrone derivatives, and phenolics are the major metabolites reported from this marine-associated fungal species (Figure 34). They could be privileged and useful candidates for chemists and biologists to design structurally novel and pharmacologically important compounds for various diseases.

**Figure 35:**
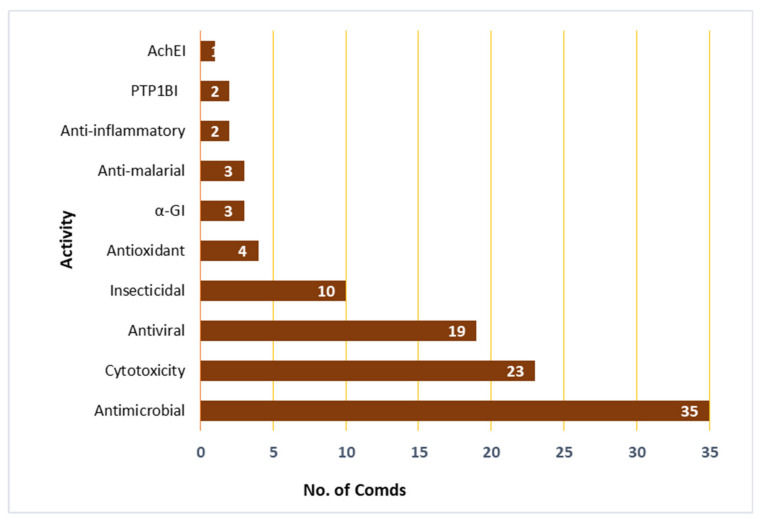
Number of bioactive compounds in each tested activity.

Although the structural diversity of these metabolites, they were insufficiently evaluated for their bioactivities. Most of them had been assessed for their antimicrobial, cytotoxicity, antiviral, and insecticidal activities (Figure 35).

**Figure 36:**
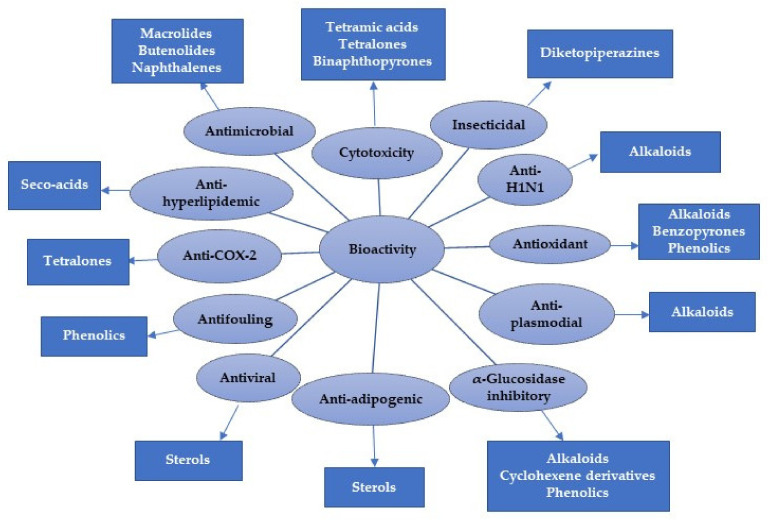
Prominent activities of each class of metabolite from *Cladosporium* species.

Figure 36 illustrated the prominent activities of each class of secondary metabolites.

However, there are limited studies that focus on the mechanism of action of these metabolites. Many of the tested metabolites possessed no noticeable efficacy in some of the tested activities. Therefore, estimation of other potential bioactivities and derivatization of these metabolites, as well as the mechanistic and in vivo studies of the active metabolites should clearly be the target of future research.

## 5. Strategies for Activating Silencing Gene Clusters

Growing evidence has revealed that the activation of silent gene clusters has the potential to significantly enhance the discovery of new natural metabolites of high-therapeutic leads. Different strategies to awake the silent biosynthetic gene clusters of *Cladosporium* species such as co-cultivation of organisms and elicitors epigenetic, as well as, modifiers can be applied [171,172,173,174]. The production of secondary metabolites (SMs) is affected by cultivation media, environment, and conditions [171,175]. Therefore, manipulating the culture conditions can improve the outputs from living organisms. Small changes in the growth media composition can induce not only variation in the amount of SMs, but also the production of a completely different pattern of molecules [171,172,173]. OSMAC (one strain many compounds) is a form of strain improvement that summarized the ability of single strains to produce different compounds when growing under different conditions e.g., aeration rate, media composition, type of culturing vessel, or a combination of these factors [174,175,176]. Challenging the fungi with external cues or chemicals has been shown to enhance the SMs production. Antibiotics have been widely reported as elicitors that can activate a broad spectrum of silent BGCs [171,172,173,174]. The co-cultivation of strains of the same or different species has been shown to represent a promising strategy for the activation of silent BGCs that enhances the production of SMs and discovery of new bioactive SMs [171,172,177]. Activation of silent biosynthetic gene clusters (BGCs) by quorum sensing class of signaling molecule is another strategy that has been shown to dramatically increase SMs production [171,172,173]. Engineering strains to circumvent the regulatory systems has the potential to free silent BGCs from their locked-in state and result in a significantly enhancement of SMs production. This can be done through various ways such as ribosome and polymerase engineering, an awakening of the genes encoding transcriptional regulatory proteins, and deletion or deactivation of the suppressor proteins. Another approach is the insertion of inducible artificial promoters to drive the expression of the silent genes [171,172,178]. Modulating epigenetic control also plays a role in the expression of silent gene clusters linked to natural product expression [173,179].

## Abbreviations

[^3^H]PDBu: [^3^H]Phorbol-12,13-dibutyrate; 22RV1: Human prostatic cancer cell line; 3T3-L1; Mouse adipocyte-like cell line; 786-0: Human renal carcinoma cell line; A2780; Human ovarian cancer cell; A375: Melanoma cell line; A549: Lung adenocarcinoma epithelial cell line; Ab1: Proto-oncogene 1, non-receptor tyrosine kinase; ABPA: Allergic bronchopulmonary aspergillosis; ADR: Adriamycin; ATGL: Adipose triglyceride lipase; ATP: Adenosine triphosphate; Bax/Bcl-2: Apoptosis regulator BAX; Bcap-37: Human breast carcinoma cell line; Bel-7402: Human hepatocellular carcinoma cell line; BGC-823: Human gastric cancer cell line; BT549: Human breast cancer cells line; C4-2B: Human prostatic cancer cell line; C4-2B: Human prostatic cancer cell line; CAM: Chick chorioallantoic membrane assay; CCK8: Cell Counting kit-8; CD25: Cluster of differentiation 25; CD28: Cluster of differentiation 28; CD3: Cluster of differentiation 3; CDK: Cyclin-dependent kinase; CDKI: Cyclin-dependent kinase Inhibitor; CDPSs: Cyclodipeptide synthases; CM: Corrected mortality; COX-2: Cyclooxygenase-2; CPE: Cytopathic effect; DAPI: 4,6-Diamidino-2-phenylindole; DELFIA: Dissociation-enhanced lanthanide fluorescence immunoassay; DKPs: Diketopiperazines; DMSO: Dimethylsulfoxide; DPPH: 1,1-Diphenyl-2-picrylhydrazyl; DU-145: Human prostate cancer cell line; ED_50_: Effective dose 50; EGFR: Epidermal growth factor receptor; ERK: Extracellular signal-regulated protein kinase; EtOAc: Ethyl acetate; EtOH: Ethanol; EV71: Enterovirus 71; FASN: Fatty acid synthase; FRAP: Ferric reducing antioxidant power; FSW: Filtered seawater; Fyn: Proto-oncogene tyrosine-protein kinase; GREs: Glucocorticoid response elements; GW9662: Selective PPAR antagonist for PPARγ; H1974: Human colorectal cancer cell line; H3N2: Influenza A virus subtype H3N2; H446: Human small cell lung carcinoma; HCC70: Human breast cancer cells line; HCT-116: Human colon cancer cell line; HCT-15: Human colon cancer cell line; HCT-8: Human colorectal cancer cell line; HeLa: Human epithelioid cervix carcinoma cell line; HeLa S3: Human cervix carcinoma cell line; HepG-2: Human liver cancer cell line; HL-60: Human promyelocytic leukemia cell; HT-29: Human colorectal adenocarcinoma cell line; HTRF: Homogeneous time-resolved fluorescence assay; Huh-7: Differentiated hepatocyte-derived cellular carcinoma cell line; IC_50_: Half-maximal inhibitory concentration; IFNγ: Interferon γ; IL-2: Interleukin-2; IZD: Inhibition zone diameter; JNK: c-Jun NH2-terminal kinase; Jurkat: Human T lymphoblastic leukemia cell lines; K562: Human kidney cancer cell line; L02: Human hepatic cancer cell line; L1210: Mouse lymphocytic leukemia cell line; L5178Y: Murine lymphoma cell line; Lac: Laccase; LBD: Ligand binding domain; LC3-I, II: Microtubule-associated proteins 1A/1B light chain 3B; LC_50_: Lethal concentration 50; LCK: Lymphocyte-specific protein tyrosine kinase; LiP: Lignin peroxidase; LLC-PK1: Epithelial-like pig kidney cell line; LM3: Human hepatocellular carcinoma cell line; LNCap: Human prostate cancer cell line; LPS: Lipopolysaccharide; MCF-7: Breast cancer cell line; MDA-MB-231: Human breast cancer cell line; MDA-MB-435: Human breast cancer cell line; MDA-MB-468: Human breast cancer cell line; MDCK: Madin–Darby canine kidney; MD-MBA-231: Breast adenocarcinoma cell line; MeOH: Methanol; MGC-803: Human gastric carcinoma cell line; MIC: Minimum inhibitory concentration; MMP: Mitochondrial membrane potential; MnP: Manganese-dependent peroxidase; MOLT-4: T lymphoblast-acute lymphoblastic leukemia; MRCNS: Methicillin-resistant coagulase-negative Staphylococci; mRNA: Messenger ribonucleic acid; MRSA: Methicillin-resistant *Staphylococcus aureus*; MTT: 3-(4,5-Dimethylthiazol-2-yl)-2,5-diphenyltetrazolium bromide; NAC: N-acetylcysteine; NCI-H460: Human lung carcinoma cell line; NF-kB: Nuclear factor Kappa B; NO: Nitric oxide; NRPSs: Non-ribosomal peptide synthetases; NS-398: N-[2-(cyclohexyloxy)-4-nitrophenyl]methanesulfonamide; OA: Oleic acid; OCI-AML3: Acute myeloid leukemia cell line; OSMAC: One strain many compounds; OVCAR-3: Human ovarian Cancer cell line; P0: PAX6 promoter; P1: PAX6 promoter; P21: N-Myc-cyclin-dependent kinase inhibitor 1A; p21waf1/cip1: Cyclin kinase inhibitor p21; P388: Mouse lymphocytic leukemia cell line; PANC-1: Human Pancreatic cancer cell line; PAX6: Paired box gene 6; PBMC: Peripheral blood mononuclear cell; PC-3: Human prostate carcinoma cell line; PG: Polygalacturonase; PGF_2__α_: Prostaglandin F_2__α_; PKA: cAMP-dependent protein kinase; PKC: Protein kinase C; PMA: Phorbol 12-myristate 13-acetate; PME: Pectin methylesterase; PPARγ: Peroxisome proliferator-activated receptor γ; PPRE: Peroxisome proliferator-activated receptor response element; PTP: Protein tyrosine phosphatase; Pα: PAX6 promoter; RHO: Rhodopsin; RSV: Respiratory syncytial virus; RWPE-1: Human normal prostate epithelial cell; SAR: Structure–activity relationship; SF-268: Human glioblastoma cell line; SGC-7901: Human gastric cancer cell line; SH-SY5Y: Human neuroblastoma cell line; SK-BR-3: Human Breast cancer cell line; SMMC-7721: Human hepatoma carcinoma cell line; SPR: Surface plasmon resonance; SRB: Sulforhodamine B; SREBP1: Sterol regulatory element binding transcription factor 1; SW1116: Human colon cancer cell line; SW1990: Human pancreatic adenocarcinoma cell line; T47D: Human breast cancer cell line; TC: Total cholesterol; TC_50_: Half toxic concentration; TCF: T cell factor; TCPTP: T Cell protein tyrosine phosphatase; TG: Total triglyceride; TRF: Time-resolved fluorescence; U937: Human myeloid leukaemia cell line; WERI: Retinoblastoma cell line; WST-1: (4-[3-4-Iodophenyl]-2-(4-nitrophenyl)-2H-5-tetrazolio)-1,3-benzene disulfonate); Y-79: Retinoblastoma cell line.
